# Probenecid Disrupts a Novel Pannexin 1-Collapsin Response Mediator Protein 2 Interaction and Increases Microtubule Stability

**DOI:** 10.3389/fncel.2018.00124

**Published:** 2018-05-11

**Authors:** Xiaoxue Xu, Leigh E. Wicki-Stordeur, Juan C. Sanchez-Arias, Mei Liu, Maria S. Weaver, Catherine S. W. Choi, Leigh A. Swayne

**Affiliations:** ^1^Division of Medical Sciences, University of Victoria, Victoria, BC, Canada; ^2^Key Laboratory of Neuroregeneration of Jiangsu and Ministry of Education, Co-Innovation Center of Neuroregeneration, Nantong University, Nanjing, China; ^3^Department of Biology, University of Victoria, Victoria, BC, Canada; ^4^Island Medical Program and Department of Cellular and Physiological Sciences, University of British Columbia, Vancouver, BC, Canada

**Keywords:** cytoskeleton, Crmp2, microtubules, neurite, neurodevelopment, neuronal polarity, Pannexin 1, probenecid

## Abstract

Neurite formation relies on finely-tuned control of the cytoskeleton. Here we identified a novel protein-protein interaction between the ion and metabolite channel protein Pannexin 1 (Panx1) and collapsin response mediator protein 2 (Crmp2), a positive regulator of microtubule polymerization and stabilization. Panx1 and Crmp2 co-precipitated from both Neuro-2a (N2a) cells and mouse ventricular zone (VZ) tissue. *In vitro* binding assays between purified proteins revealed the interaction occurs directly between the Panx1 C-terminus (Panx1 CT) and Crmp2. Because Crmp2 is a well-established microtubule-stabilizing protein, and we previously observed a marked increase in neurite formation following treatment with the Panx1 blocker, probenecid, in N2a cells and VZ neural precursor cells (NPCs), we investigated the impact of probenecid on the Panx1-Crmp2 interaction. Probenecid treatment significantly disrupted the Panx1-Crmp2 interaction by both immunoprecipitation (IP) and proximity ligation analysis, without altering overall Crmp2 protein expression levels. In the presence of probenecid, Crmp2 was concentrated at the distal ends of growing neurites. Moreover, probenecid treatment increased tubulin polymerization and microtubule stability in N2a cells. These results reveal that probenecid disrupts a novel interaction between Panx1 and the microtubule stabilizer, Crmp2, and also increases microtubule stability.

## Introduction

Pannexin 1 (Panx1) was first discovered about 20 years ago (Panchin et al., 2000) and forms large-pore membrane channels that mediate passage of ions and metabolites (reviewed in Chiu et al., 2018; Whyte-Fagundes and Zoidl, 2018). Panx1 channels facilitate ATP release from cells, implicating them in many physiological and pathophysiological contexts (reviewed in Velasquez and Eugenin, 2014; Dahl, 2015; Lapato and Tiwari-Woodruff, 2018). Although widely expressed throughout the body, Panx1 is enriched in the brain (Penuela et al., 2007) where it is linked to synaptic plasticity, and learning and memory (reviewed in Boyce et al., 2018).

Previous work from our lab identified Panx1 as a new player in the regulation of neurites (Wicki-Stordeur and Swayne, 2013). We discovered the expression of Panx1 in neural precursor cells (NPCs) and developing neurons of the postnatal ventricular zone (VZ; Wicki-Stordeur et al., 2012; Wicki-Stordeur and Swayne, 2013). Using a combination of models, mouse neuroblastoma Neuro-2a (N2a) cells and VZ NPCs, we made several key observations suggesting Panx1 is a negative regulator of neurite formation (Wicki-Stordeur and Swayne, 2013). Firstly, we found that endogenous N2a cell Panx1 expression levels undergo a striking reduction over the course of retinoic acid differentiation, during which these cells also form long neurites. We further observed that direct reduction of Panx1 activity or expression increased neurite formation, while Panx1 overexpression inhibited neurite formation under retinoic acid stimulation. More recently, work from another group supported these findings, demonstrating that disruption of Panx1 (block or KO) increased axonal caliber and growth rate in dorsal root ganglion neurons (Horton et al., 2017). Together these data from others and us suggest that Panx1 is a negative regulator of neurite extension and/or stability, however the underlying mechanism has not yet been resolved.

To shed light on the molecular processes involved, we turned to Panx1 protein interaction partners. We previously conducted a study to identify novel Panx1-interacting proteins and found that many of these were cytoskeleton associated proteins, including components of the Arp2/3 complex (Wicki-Stordeur and Swayne, 2013). Another key regulator of neurite formation identified in this screen, but not previously validated, was collapsin response mediator protein 2 (Crmp2; *Dpysl2)*. Crmp2 is a microtubule-associated protein that plays a key role in neurite formation (Gu and Ihara, 2000; Fukata et al., 2002; Suzuki et al., 2003; Crews et al., 2011; Lin et al., 2011; Higurashi et al., 2012; Wilson et al., 2014; reviewed in Quach et al., 2015; Takano et al., 2015) by enhancing microtubule polymerization and by directly binding to tubulin dimers at the plus-end of microtubules (Niwa et al., 2017). Capitalizing on the striking neurite outgrowth we observed in the presence of probenecid, a Panx1 blocker (Silverman et al., 2008), here we investigated whether probenecid modulates the Panx1-Crmp2 interaction and microtubule stability.

## Materials and Methods

### Animals

All procedures were carried out in accordance with the recommendations of the Canadian Council for Animal Care and the University of Victoria Animal Care Committee. The protocol was approved by the University of Victoria Animal Care Committee. For Western blot time course analyses of Panx1 and Crmp2 expression in the VZ, C57BL/6J mice were sacrificed at P0, P7, P10, P28 and P60. The VZ were removed by dissection and processed for SDS-PAGE/Western blot analysis as indicated below.

### Cell Culture

Wildtype N2a cells (ATCC, CCL-131) and N2a cells stably expressing Panx1-EGFP (described previously in Boyce et al., 2015; Boyce and Swayne, 2017) were cultured in DMEM/F12 (Thermo Fisher Scientific, 11330-032) supplemented with 10% fetal bovine serum (Thermo Fisher Scientific, 12483-020), 100 U/mL penicillin and 100 μg/mL streptomycin (Thermo Fisher Scientific, 15140-122). For probenecid assays, wildtype N2a cells were seeded at 2.1 × 10^4^ cells/cm^2^, and 24 h later the (complete) culture medium was supplemented with 1 mM probenecid (Thermo Fisher Scientific, P36400) or vehicle control for 16 h. Where indicated, wildtype N2a cells were transfected using jetPEI (Polyplus-transfection, 101-10N), according to the manufacturer’s instructions with Panx1EGFP (generously provided by Drs. Dale Laird and Silvia Penuela, University of Western Ontario, London, ON, Canada) or pEGFP-N1 control plasmid to express EGFP.

### Antibodies

Primary antibodies used in this study were mouse anti-Crmp2 monoclonal (1:100 [immunostaining] or 1:6000–1:8000 [Western blotting]; Novus, NBP1-50580), rabbit anti-Crmp2/Toad64 polyclonal (1:4000–1:10,000 [Western blotting]; Bioss Antibodies, BS-1790R), mouse anti-GFP monoclonal (1:1000; MilliporeSigma, 11814460001 ROCHE), rabbit anti-GFP polyclonal (1:10,000; Thermo Fisher Scientific, A6455), rabbit anti-Panx1 CT395 (1:500–1:4000; a generous gift from Drs. Dale Laird and Silvia Penuela, University of Western Ontario, London, ON, Canada), rabbit anti-Panx1 (D9M1C) monoclonal antibody (Cell Signaling Technology, 91137S), rabbit anti-Panx1 EL2 (a generous gift from Drs. Dale Laird and Silvia Penuela), sheep polyclonal anti-alpha/beta tubulin (1:1500; Cytoskeleton Inc., ATN02), mouse anti-acetylated tubulin monoclonal (1:2000; MilliporeSigma, T6793), rat anti-tyrosinated tubulin polyclonal (1:1000; EMD Millipore, MAB1864-I). Secondary antibodies were Alexa Fluor 488-conjugated AffiniPure Donkey Anti-Mouse IgG (1:300, Jackson Immunoresearch, 715-545-151), horseradish peroxidase (HRP)-conjugated AffiniPure donkey anti-rabbit IgG (1:2000–1:4000, Jackson Immunoresearch, 711-035-152), HRP-conjugated AffiniPure donkey anti-mouse IgG (1:2000–1:4000, Jackson Immunoresearch, 715-035-150), HRP-conjugated AffiniPure donkey anti-sheep (1:1500, Jackson Immunoresearch, 713-035-147), and HRP-conjugated donkey anti-rat (1:3000, Jackson Immunoresearch, 712-035-153).

### Western Blot Analysis

Samples were homogenized in either PBS (150 mM NaCl, 9.1 mM Na_2_HPO_4_, 1.7 mM NaH_2_PO_4_) or TBS (50 mM Tris, pH 8.0, 150 mM NaCl) based RIPA buffer (1% IGEPAL, 0.5% sodium deoxycholate, 0.1% SDS) supplemented with protease inhibitor cocktail at 1 μL/10^6^ cells (stock: 104 mM 4-(2-aminoethyl)benzenesulfonyl fluoride hydrochloride, 0.08 mM aprotinin, 4 mM bestatin hydrochloride, 1.4 mM N-(trans-epoxysuccinyl)-L-leucine 4-guanidinobutylamide, 2 mM leupeptin hemisulfate salt, 1.5 mM pepstatin-A; MilliporeSigma, P8340), PMSF at 2 μL/10^6^ cells, 10 μM sodium orthovanadate and 1 mM EDTA for 30 min, and centrifuged at 4°C for 20 min at 12,000 rpm to remove debris. Samples were heated to 95–100°C for 5–20 min in SDS-PAGE loading dye under reducing conditions (dithiothreitol and β-mercaptoethanol) before loading onto gels. Gels were transferred to 0.2 μm polyvinyldene fluoride (PVDF; Bio-rad, 162-0177) membrane for 1 h at 85–95 V, or 16–18 h at 22 V. Transfer was confirmed by Ponceau S (MilliporeSigma, P7170) total protein staining. Blocking and antibody incubations were performed in 5% skim milk, or 5% bovine serum albumin (BSA; MilliporeSigma, A2153), both with 0.1% Tween 20. Immunoreactive bands were visualized via enhanced chemiluminescence imaging using a G:BOX Chemi XR5 (Syngene) and quantified by densitometry measurements using ImageJ^1^.

### Tubulin Polymerization *in Cellulo*

Assessment of tubulin polymerization (stability) in cells was performed as described previously (Wang et al., 2010) with modifications. Following probenecid or vehicle control treatments, N2a cells were rinsed twice at 37°C with pre-warmed PME buffer (100 mM PIPES, pH 6.9, 1 mM MgCl_2_, 2 mM EGTA), then harvested in PME buffer supplemented with 0.05% TritonX-100 and protease inhibitors. The lysates were centrifuged at 13,000× *g* for 20 min at 20°C. The supernatant (S fraction) containing solubilized tubulin and the pellet (P fraction) containing polymerized tubulin were collected separately and processed for analysis by Western blotting. Equal loading of fractions was determined by Ponceau S staining of the PVDF membrane.

### Tubulin Polymerization *in Vitro*

Tubulin polymerization *in vitro* was measured using the Tubulin Polymerization Assay kit (Cytoskeleton, BK011P) according to the manufacturer’s instructions. Concentrations of probenecid used were 200 μM and 1 mM.

### GFP Immunoprecipitations

These experiments were performed as previously described (Wicki-Stordeur and Swayne, 2013). Briefly, Panx1EGFP and EGFP expressing N2a cells were collected 96 h following transfection. Approximately 4.5 × 10^7^ cells per condition were homogenized on ice in RIPA buffer supplemented with protease inhibitor cocktail and PMSF for 30 min, followed by centrifugation at 4°C for 20 min at 12,000 rpm to remove debris. Lysates were pre-cleared for 45–60 min with protein-G agarose beads (MilliporeSigma, 11243233001 ROCHE) at 4°C with shaking, then added to 200 μL protein-G bead suspension cross-linked with 5 μg of GFP monoclonal antibody (MilliporeSigma, 11814460001 ROCHE), and incubated overnight at 4°C with shaking. Beads were washed once with RIPA buffer and twice with PBS, then eluted in two bead volumes of 0.5 M ammonium hydroxide/0.5 mM EDTA for 30 min at room temperature with shaking. Probenecid treatment details are provided in the figure legends and text.

### Endogenous Immunoprecipitations

N2a cells (4.5 × 10^7^ cells/immunoprecipitation (IP)), or VZ tissue dissected from pooled P0–P10 or P60 C57BL/6J mice were homogenized in TBS lysis buffer (10 mM Tris base, pH 7.4, 150 mM NaCl, 1% IGEPAL), supplemented with protease inhibitor cocktail, PMSF, and sodium orthovanadate, for 30 min on ice, followed by centrifugation at 4°C for 20 min at 12,000 rpm to remove debris. The supernatant was pre-cleared for 45–60 min with protein-A agarose beads (MilliporeSigma, 11134515001 ROCHE) cross-linked to ChromPure rabbit IgG (Jackson ImmunoResearch, 011-000-003) at 4°C with shaking. Pre-cleared lysate (1.5–2.5 mg) was added to 200 μL protein-A bead suspension cross-linked with 5 μg of Panx1-EL2 antibody (generously provided by Drs. Dale Laird and Silvia Penuela, University of Western Ontario, Canada), Crmp2 polyclonal antibody (Bioss Antibodies, BS-1790R), or ChromPure rabbit IgG control, and incubated 1.5 h at 4°C with shaking. Beads were washed 2–3 times with TBS/0.5% IGEPAL and four times with TBS, then eluted in two bead volumes of 0.5 M ammonium hydroxide/0.5 mM EDTA for 30 min at room temperature with shaking. The eluent was dried and rehydrated in TBS lysis buffer with SDS-PAGE loading dye under reducing conditions to analyze by Western blotting.

### *In Vitro* Binding Assays

To generate Panx1 C-terminus (Panx1CT)-GST plasmid, Panx1CT sequence (amino acids 299–426) was cloned from the Panx1-EGFP plasmid (forward primer: ACTTTGGAATTCTCGGCAGAAAACGGAC, reverse primer: TGCTATCTCGAGTTAGCAGGACGGAT). The Panx1CT sequence and pGEX-4T-3 plasmid (GE Healthcare Lifesciences, 27-4583) were digested with EcoRI and XhoI, gel purified (Qiagen, 28704), and ligated overnight. All recombinant proteins for *in vitro* binding assays were generated in BL21 *Escherichia coli* (*E. coli*; New England Biolabs, C2527I). BL21 *E. coli* were transformed with plasmids encoding Panx1CT-GST, Crmp2-GST (a generous gift from Dr. Rajesh Khanna, University of Arizona, Tucson, AZ, USA), or GST control plasmid (pGEX-4T-3) according to the manufacturer’s instructions, and grown up overnight on LB agar at 37°C. The following day, single colonies were picked and grown in 50 mL LB broth at 37°C overnight. LB broth was added (200 mL) and the cultures were grown 2 h at 37°C, then induced with IPTG (1 mM) at 37°C for 4 h. The bacteria were pelleted and re-suspended in 10 mL cold re-suspension buffer (PBS, 0.05% Tween 20, 2 mM EDTA, 0.1% β-mercaptoethanol) before being lysed by 2–3 passages through a French press at ~1100 psi. GST fusion proteins were recovered by binding with glutathione-sepharose beads (Thermo Fisher Scientific, G4510) overnight at 4°C, then washed three times with 0.5 mL RIPA, twice with 0.5 mL RIPA without the SDS, three times with 0.5 mL PBS, and then re-suspended to 50% with PBS. To estimate protein concentration, the final products were compared with BSA standards of known microgram amounts on Colloidal Blue-stained (Thermo Fisher Scientific, LC6025) SDS-PAGE gels. Purified Panx1CT and Crmp2 were removed from the beads by cleavage with thrombin (GE Healthcare Lifesciences, 27-0846-01), or Factor Xa (New England Biolabs, P8010L), respectively. The beads were washed twice in thrombin cleavage buffer (50 mM Tris-HCl pH 8.0, 150 mM NaCl, 2.5 mM CaCl_2_, 0.1% β-mercaptoethanol), or Factor Xa cleavage buffer (20 mM Tris-HCl, pH 8.0, 100 mM NaCl, 2 mM CaCl_2_) then incubated in 250 μL buffer with 10 μL thrombin or Factor Xa at room temperature for 2 h (thrombin) or 4 h (Factor Xa). PMSF (15 μL) was added to the beads, incubated for 15 min, and repeated once more. The supernatants were removed and analyzed by SDS-PAGE for concentration (against BSA standards) and confirmation of cleavage. For the *in vitro* binding assay, purified Crmp2 (0.03 nmol) or Panx1CT (0.3 nmol) was incubated with 0.034 nmol Panx1CT-GST or Crmp2-GST coupled to glutathione agarose beads at 4°C for 1 h with shaking. Beads were washed 2–3 times with RIPA buffer, two times with TBS/1% IGEPAL and three times with TBS, eluted by boiling at 100°C in SDS-PAGE loading dye under reducing conditions, and analyzed by Western blotting.

### PNGase F Deglycosylation

Panx1EGFP transfected N2a cells were lysed in TBS- or PBS-RIPA. 10–15 μg total protein underwent PNGase F (New England Biolabs, P0704) reaction, according to the manufacturer’s manual. Samples were analyzed by Western Blot probed with anti-Panx1 (D9M1C) rabbit monoclonal antibody (Cell Signaling Technology, 91137S) and rabbit anti-GFP polyclonal antibody (Thermo Fisher Scientific, A6455).

### Confocal Immunofluorescence Microscopy

N2a cells plated on poly-D-lysine (PDL; MilliporeSigma, P6407)-coated coverslips were fixed with 4% paraformaldehyde for 10 min, then washed three times with PBS before immunostaining. Antibodies were diluted in 10 mM PBS supplemented with 0.3% Triton-X-100 and 3% normal donkey serum (Jackson Immunoresearch, 017-000-121). Confocal imaging and analysis were performed blind to the treatment conditions using a Leica SP8 confocal microscope and Leica Application Suite Software version 3.1.3.16308. Images were imported to ImageJ (Schneider et al., 2012,^2^), and the background was subtracted uniformly from each channel prior to analysis. Representative images were uniformly adjusted using Adobe Photoshop CS5 Extended software (Adobe Systems Incorporated). Neurites were defined as extensions from the cell body equal to or greater than one corresponding cell body diameter in length (Wicki-Stordeur and Swayne, 2013), and traced from their place of origin at the soma to their tips; only the longest neurite was included for subsequent analysis. In order to quantify the localization of Crmp2 following probenecid treatment, line scans were performed on each traced neurite, and mean fluorescence intensities were grouped in four equal neurite segments: the first three segments, closest to the soma, were defined as the neurite “shaft”, while the last segment, farthest from the soma, was defined as the neurite “tip”. A ratio was then calculated for each neurite between the average intensity in the neurite tip and the average intensity in the corresponding neurite shaft. These ratio data were grouped by total neurite length (10–30 μm, 30–50 μm, >50 μm) and graphed against a hypothetical value of 1. Data were obtained from 126 neurites (n), obtained from 75 cells from three independent cultures, for a total of 75 traced cells.

### Proximity Ligation Assay

Proximity ligation assay (PLA) was carried out with the Duolink PLA kit (MilliporeSigma, DUO92013) according to the manufacturer’s instructions. N2a cells stably expressing Panx1-EGFP (described previously in Boyce and Swayne, 2017) were plated on PDL-coated coverslips with selection antibiotic geneticin 418, grown for 24 h, and treated with 1 mM probenecid or vehicle control for 16 h. Cells were fixed for 10 min in 4% paraformaldehyde. After several rinses in PBS, coverslips were incubated with a mixture of rabbit polyclonal anti-GFP (1:500, Thermo Fisher Scientific, A6455) and monoclonal anti-Crmp2 (1:100, Novus Biologicals, NBP1-50580) antibodies, rabbit polyclonal anti-GFP and mouse anti-GFP monoclonal (1:50, MilliporeSigma, 11814460001 ROCHE) antibodies (positive control), or in antibody buffer alone (negative control) at 4°C overnight. The coverslips were incubated with PLA probe anti-rabbit PLUS and PLA probe anti-mouse MINUS (each at 1:5 dilution) for 60 min at 37°C in a humid chamber. Image acquisition and analysis were performed blind to the treatment conditions. Confocal z-stacks of randomly selected ROIs were acquired on a Leica TCS SP8 confocal microscope using the “Mark and Find” and “Autofocus” tools of the Leica Application Suite Software, version 3.1.3.16308. Image files were imported into Fiji for subsequent analysis (Schindelin et al., 2012,^3^). We used a semi-automated protocol to generate regions of interest (ROIs) consisting of cell somas together with their respective neurites. In brief ROI masks were obtained using, maximum intensity projections of the EGFP signal that were thresholded and binarized. These were subjected to automatic detection of particles with size = 10,000–300,000 pixels (1 pixel = 0.076 μm) and a circularity of 0.1–1.0. These parameters identified individual cellular ROIs for the majority of cells and were further curated for inaccuracies (e.g., large cell clumps were excluded; while still blinded to the treatment conditions). Neurites traced manually and were combined to their respective soma(s) to create complete individual cellular ROIs (neurite + soma). The fluorescence intensity (mean gray value, arbitrary unit, arb.u.) within each of these ROIs was calculated and normalized to its respective ROI area. This value was then multiplied by 100 to obtain the PLA fluorescence intensity per 100 μm^2^. All cells included in the analysis were evaluated for their wellness status and signs of stress (e.g., blebbing, large vacuoles; Frigault et al., 2009) using transmitted light microscopy (while blinded to the treatment conditions). Data were obtained from *n* = 77 cells (vehicle group) and *n* = 70 cells (probenecid group) from three independent passages.

### Statistical Analysis

Statistical analyses were performed using Prism Windows v5.01 and Mac OS X v5.0d software (GraphPad Software^4^), and SPSS Statistics for Windows (v25, IBM) For statistical analysis, variables and their residuals were tested for normality using the Shapiro-Wilk test (null hypothesis: data follow a normal distribution; alternative hypothesis: data do not follow a normal distribution). For variables that fit a normal distribution, parametric tests were used for comparisons, while non-parametric tests were performed for variables that rejected the null hypothesis (i.e., data are not normally distributed). The complete description of the normality tests are found in the Supplementary Material. Statistical tests are reported in each figure legend. All variances are reported as standard error of the mean. Significance is denoted as *P* < 0.05 (*), *P* < 0.01 (**), *P* < 0.001 (***), *P* < 0.0001 (****). Exact *P* values and sample sizes are provided in the Figure legends.

## Results

### Panx1 Interacts Directly With the Microtubule Stabilizer Crmp2 Via the Panx1CT

**Figure 1:**
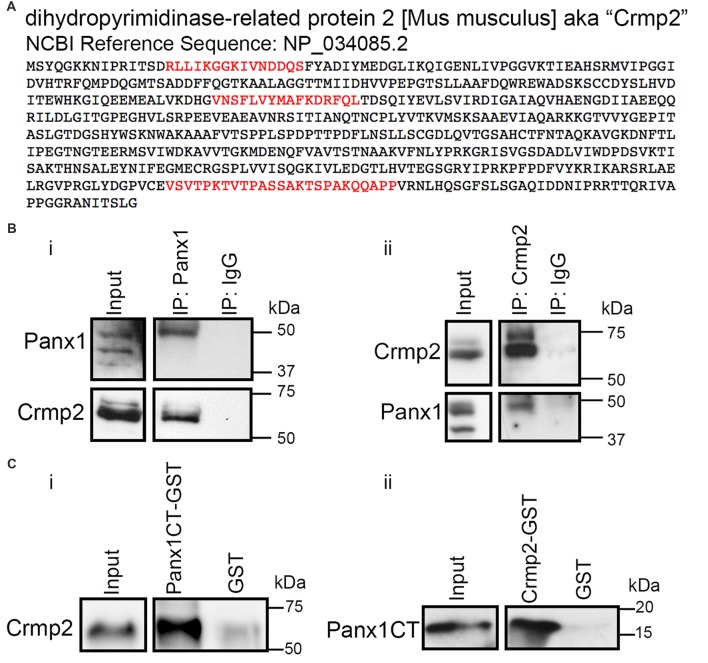
Pannexin 1 (Panx1) and collapsin response mediator protein 2 (Crmp2) interact.** (A)** Crmp2 peptides (red) identified previously by immunoprecipitation (IP) coupled to LC-MS/MS in Wicki-Stordeur and Swayne (2013). Peptides were identified specifically in IPs from Panx1-EGFP expressing Neuro-2a (N2a) cells, and not EGFP controls. **(B)** Western blot of endogenous IPs from N2a cell lysates. **(i)** Crmp2 precipitates specifically with Panx1 compared with IgG control. **(ii)** Panx1 precipitates specifically with Crmp2 compared with IgG control. **(C)** Western blots of an *in vitro* binding assay using purified Crmp2 and Panx1 C-terminus (Panx1CT). **(i)** The Panx1CT enriches with immobilized Crmp2-GST compared with GST control. **(ii)** Crmp2 enriches with Panx1CT-GST compared with GST control. These results are representative of three independent replicates. For the complete blots of parts **(B,C)**, see Supplementary Material. This data was previously included in the PhD thesis of LWS (link: https://dspace.library.uvic.ca/handle/1828/6938).

We previously identified Panx1 as a negative regulator of neurite outgrowth in N2a cells and primary VZ NPCs (Wicki-Stordeur and Swayne, 2013); however, the underlying mechanisms remained unknown. To gain further insight, we mined our previously described Panx1 interactome (Wicki-Stordeur and Swayne, 2013) for proteins with established role(s) in neurite outgrowth or stabilization. Based on a substantial body of evidence that has established it as a microtubule-associated protein that plays a critical role in neurite stability (reviewed in Ip et al., 2014; Quach et al., 2015), we decided to focus our attention on Crmp2 (*Dpysl2*; Figure 1A).

Reciprocal IPs with antibodies for Panx1 and Crmp2 confirmed the endogenous co-precipitation of these proteins (Figure 1B). Crmp2 co-enriched with antibodies to Panx1 but not control IgG. Similarly, Panx1 co-precipitated specifically with antibodies to Crmp2. In order to determine whether the Panx1-Crmp2 interaction was direct, we performed *in vitro* binding assays using purified epitope-tagged proteins (Figure 1C). We found that the Panx1CT enriched specifically with purified Crmp2-GST bound to glutathione-agarose beads, but not to control GST. Similarly, Crmp2 was enriched by Panx1CT-GST but did not interact with control GST. Taken together, these data confirm that Panx1 and Crmp2 interact directly through the Panx1CT.

### The Panx1/Crmp2 Interaction Occurs in the VZ *in Vivo*

**Figure 2:**
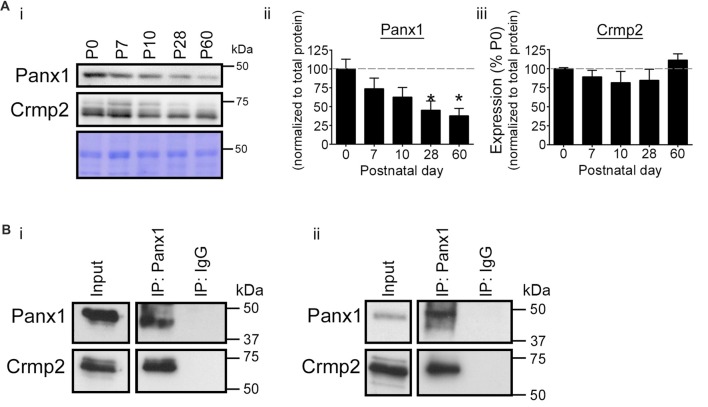
Validation of the Panx1-Crmp2 interaction in the mouse ventricular zone (VZ). **(Ai)** Western blot of a time course of Panx1 and Crmp2 expression in the VZ. **(ii)** Panx1 expression peaked within the early postnatal period (P0–P7), and dramatically decreased with age (P28–P60; *p* = 0.0365 by one-way analyses of variance (ANOVA); *F*_(4,10)_ = 3.9; Dunnett’s multiple comparison test *post hoc*, P28 *p* = 0.0352, and P60, *p* = 0.0177; *n* = 3). Coomassie Blue was employed as a loading control. **(iii)** Crmp2 expression remained relatively constant across this time course (*F*_(4,10)_ = 1.3; *p* = 0.3333, one-way ANOVA; *n* = 3). **(B)** Western blot of endogenous Panx1 IPs from mouse brain VZ tissue. Crmp2 co-precipitated specifically with Panx1 from young (P10; **i**) and adult (P60; **ii**) mouse VZ. For the complete blots of part **(B)**, see Supplementary Material. This data was previously included in the PhD thesis of LWS (link: https://dspace.library.uvic.ca/handle/1828/6938).

We next investigated postnatal co-expression of Panx1 and Crmp2 in the mouse VZ *in vivo*. Western blotting demonstrated the two proteins were present in VZ tissue across a range of ages (P0–P60; Figure 2A). While Crmp2 expression remained relatively constant across the time range analyzed, Panx1 expression levels peaked close to birth, then dramatically decreased with age. Reciprocal IP experiments in VZ tissues from both young and old mice (Figure 2B) confirmed the Panx1-Crmp2 interaction also occurred* in vivo*.

### Probenecid Treatment Decreases Crmp2 Association With Panx1, Without Altering Crmp2 Expression Levels

**Figure 3:**
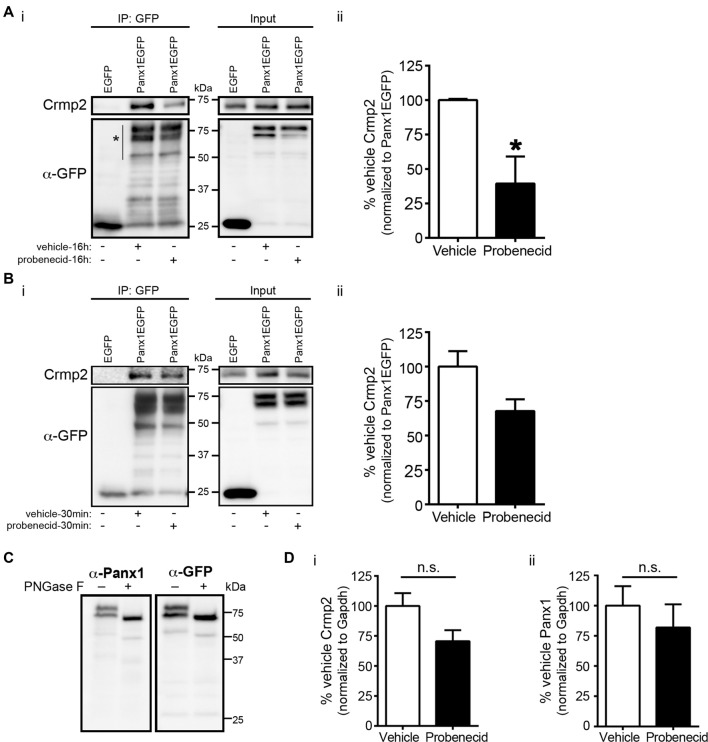
Probenecid treatment decreases Crmp2 co-precipitation with Panx1. **(Ai)** Western blot of a GFP IP from N2a cells expressing Panx1-EGFP, or EGFP control, treated (16 h) with probenecid or vehicle control. **(ii)** Probenecid treatment (16 h) significantly decreased the amount of Crmp2 that precipitated with Panx1-EGFP. Crmp2 signal in the IP fraction was normalized to GFP signal intensity from the same lane (Panx1-EGFP [vehicle] vs. Panx1-EGFP [probenecid]: 100.0 ± 0.8 vs. 39.4 ± 19.6, *p* = 0.0368, *n* = 3, unpaired *t*-test). **(B)** The same assay was performed with a shorter 30 min treatment **(i)**. **(ii)** Short probenecid treatment (30 min) revealed a ~30% decrease in Crmp2 co-precipitation (Panx1-EGFP [vehicle] vs. Panx1-EGFP [probenecid]: 100.0 ± 11.1 vs. 67.7 ± 19.6, *p* = 0.0826, *n* = 3, unpaired *t*-test). **(C)** PNGase F treatment was performed to confirm the upper Panx1EGFP immunoreactive band was indeed glycosylated Panx1 and assessed by Western blotting. Note that both immunoreactive bands shifted in response to N-glycosidase F treatment, suggesting that both major bands are mature glycosylated Panx1EGFP of differing molecular weights. **(D)** Analysis of the input intensities revealed that probenecid treatment did not change the overall expression of **(i)** Crmp2 (*p* = 0.2000, Mann-Whitney test, *n* = 3) or **(ii)** Panx1 (*p* = 0.578, unpaired *t*-test, *n* = 3). Normalized to Gapdh (not shown), n.s., non-significant.

Our previous work demonstrated that Panx1 negatively regulates neurite outgrowth (Wicki-Stordeur and Swayne, 2013). Knockdown of Panx1 expression with siRNA and Panx1 block with probenecid (Silverman et al., 2008) increased neurite outgrowth; conversely, Panx1 overexpression decreased neurite outgrowth. Because Crmp2 is a positive regulator of microtubule stabilization and neurite extension, we hypothesized that probenecid promotes neurite outgrowth by releasing Crmp2 from its direct interaction with Panx1. In support of this hypothesis, we found that 16 h probenecid treatment (the treatment time for which we observed marked increased neurite outgrowth in our previous report) significantly (~60%) decreased the relative amount of Crmp2 co-precipitating with Panx1 compared to vehicle control treatment (Figure 3A). Importantly, we observed no change in overall Crmp2 expression levels as demonstrated by intensity analysis of the inputs. Further, short term probenecid treatment (30 min) was sufficient to produce a ~30% decrease that approached statistical significance (*p* = 0.085).

**Figure 4:**
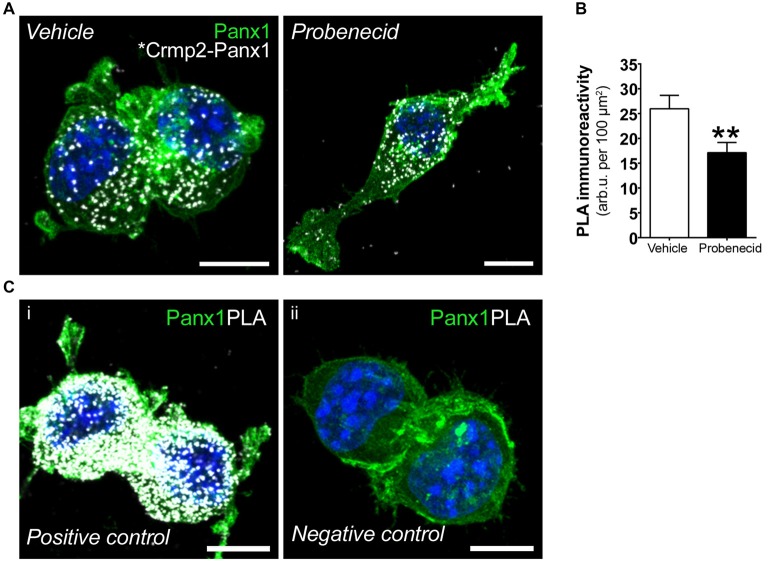
Probenecid decreases proximity ligation assay (PLA) between Panx1EGFP and Crmp2.** (A)** Representative confocal maximum intensity projections of vehicle- (left) and probenecid-treated (right) Panx1-EGFP-expressing N2a cells demonstrating PLA-positive Crmp2-Panx1 clusters (white). **(B)** Probenecid treatment significantly decreased mean fluorescence intensity of the PLA signal (vehicle: 26.0 ± 2.7 arb.u. per 100 μm^2^ vs. probenecid: 17.1 ± 2.1 arb.u. per 100 μm^2^, *p* = 0.0037, *n* = 77 cells and *n* = 70 cells, respectively, Mann-Whitney test). **(C)** Controls for PLA included **(i)** cells were incubated with α-GFP anti-mouse and α-GFP anti-rabbit to serve as a positive control. **(ii)** No primary antibodies were included to serve as a negative control. Hoechst 33342 was used as a nuclear counterstain. Scale bars, 10 μm.

**Figure 5:**
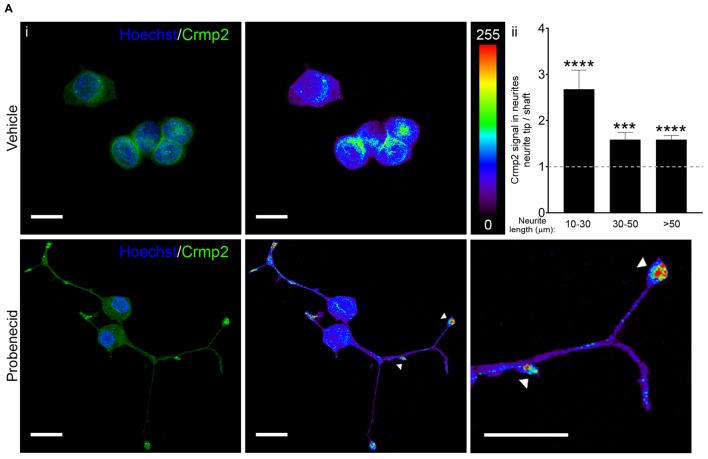
Probenecid treatment results in Crmp2 concentration at neurite tips. **(Ai)** Representative confocal images from N2a cells treated with vehicle (upper panels) or probenecid (lower panels) and immunostained for Crmp2. Crmp2 signal intensity is indicated in the heat maps. Hoechst 33342 was used as a nuclear counterstain. Scale bars: 20 μm. **(ii)** Crmp2 signal in probenecid-induced neurites was more abundant in neurite tips compared to the shaft, regardless of neurite length (10–30 μm, *p* < 0.0001, *n* = 38 neurites; 30–50 μm, *p* = 0.0003, *n* = 40 neurites; >50 μm, *p* < 0.0001, *n* = 48 neurites; Wilcoxon signed-rank test against a hypothetical median value of 1.0).

We next used PLA to confirm the effects of probenecid treatment on the Panx1-Crmp2 interaction (Figure 4). We employed N2a cells stably expressing low levels of Panx1-EGFP (Boyce et al., 2015; Boyce and Swayne, 2017) in order to use anti-GFP and anti-Crmp2 antibodies for the assessment of proximity between the two proteins. The PLA signal (bright white dots) is generated where Panx1EGFP and Crmp2 are within 40 nm (Söderberg et al., 2006), and these dots can therefore be interpreted as the subcellular loci of their interaction. The distribution pattern of Panx1EGFP was consistent with our previous reports (Boyce et al., 2015; Boyce and Swayne, 2017), with enrichment at the plasma membrane, but also an intracellular punctate population that we recently demonstrated is due to constitutive ATP release and ATP-induced internalization of Panx1 to endosomal compartments (note that these are maximum intensity projections shown in contrast to confocal z-sections shown in our previous reports). Cells treated with probenecid (16 h) extended long neurites, much longer than those spontaneously arising in vehicle-treated cells, as previously reported and quantified in our original report (Wicki-Stordeur and Swayne, 2013). In vehicle-treated cells, the PLA signal was observed throughout the cell, from the plasma membrane, where Panx1 is relatively enriched (as per the green EGFP signal), to the peri-nuclear region, where Crmp2 is relatively enriched (as demonstrated by confocal immunocytochemistry in Figure 5). In probenecid-treated cells, PLA signal was observed in both cell body and neurites. Probenecid treatment (16 h) significantly reduced the intensity of the PLA signal compared with vehicle controls, in agreement with our IP results.

### Probenecid Treatment Leads to Concentration of Crmp2 at Microtubule Plus Ends and Increases Microtubule Polymerization and Stability

**Figure 6:**
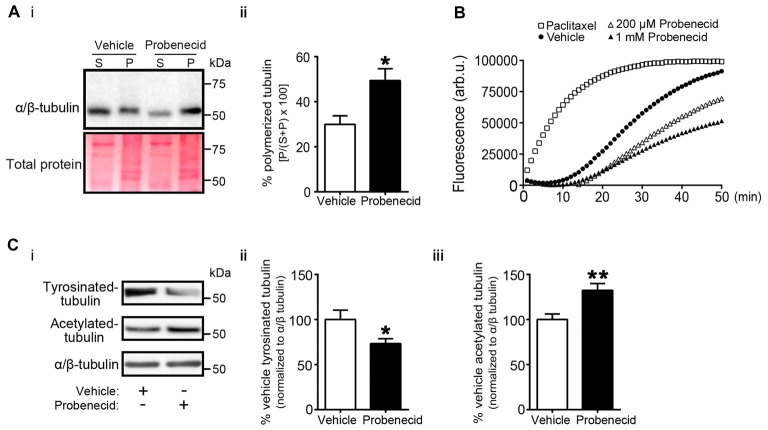
Probenecid treatment increases microtubule stability in cells. **(Ai)** N2a cells were treated with vehicle control or probenecid and then fractionated to obtain polymerized and soluble microtubule fractions. P (pellet) contains the polymerized microtubule fraction and S (supernatant) contains the soluble tubulin fraction. Ponceau S staining was employed as a loading control. **(ii)** Probenecid enhanced the percentage of polymerized microtubules to soluble tubulin (vehicle: 29.9 ± 3.8% vs. probenecid: 49.4 ± 5.2%, *p* = 0.0107 by unpaired* t*-test; *n* = 7). **(B)** Tubulin polymerization assays *in vitro*. Paclitaxel (3 μM) and vehicle were employed as positive and negative controls, respectively. The experiment was performed three times, with the replicates as follows: paclitaxel *n* = 5; Vehicle *n* = 5; 200 μM Probenecid *n* = 5; 1 mM Probenecid *n* = 7. **(Ci)** Western blot of post-translationally modified tubulin species from N2a cells treated with probenecid or vehicle control. Tyrosinated-tubulin represents the dynamic population, and acetylated-tubulin represents the stable population. Total α/β-tubulin was used for normalization. Probenecid treatment **(ii)** decreased tyrosinated-tubulin (100.0 ± 10.5% vs. 73.3 ± 5.5%, *p* = 0.0377 by unpaired *t*-test; *n* = 9) and **(iii)** increased acetylated-tubulin (100.0 ± 6.1% vs. 132.0 ± 7.6%, *p* = 0.0043 by unpaired *t*-test; *n* = 9).

We then examined the distribution of Crmp2 in vehicle-treated and probenecid-treated N2a cells using confocal immunocytochemistry (Figure 5A). In control, vehicle-treated cells, Crmp2 was enriched in the peri-nuclear region. In the presence of probenecid, Crmp2 immunoreactivity was concentrated at the distal tips of the probenecid-induced neurites. Next, based on the well-documented role of Crmp2 as a microtubule stabilizing protein, we predicted that release of Crmp2 from Panx1 and its observed concentration at distal neurite tips would likely lead to increased microtubule stability. To first test this hypothesis, we treated N2a cells with probenecid or vehicle control, then analyzed the amount of polymerized and soluble tubulin within each sample (Figure 6A). Probenecid treatment significantly increased the ratio of polymerized:soluble tubulin within N2a cells, suggesting that probenecid increased microtubule stability. In order to determine whether this was due to a direct impact of probenecid on tubulin polymerization, we carried out an *in vitro* tubulin polymerization assay. Our results (Figure 6B) showed that probenecid did not directly promote the assembly of tubulin to MTs* in vitro*, supporting the idea that probenecid’s impact on MT stability in cells occurred through an indirect mechanism. To further validate the impact of probenecid on microtubule stability, we examined two tubulin post-translational modifications (Figure 6C): tyrosination, an indicator of dynamic tubulin, and acetylation, an indicator of stable tubulin (Kollins et al., 2009). Probenecid treatment decreased tyrosinated tubulin, and increased acetylated tubulin compared to vehicle control. Together these results suggest that probenecid enhances microtubule stability through an indirect mechanism.

## Discussion

Our previous work (Wicki-Stordeur and Swayne, 2013) demonstrated that Panx1 acts as a negative regulator of neurite outgrowth in N2a cells and primary VZ NPCs. Here we expand on that work, focusing on our discovery of an interaction between Panx1 and a microtubule-associated protein, Crmp2, and the robust induction of neurite outgrowth in the presence of the Panx1 blocker, probenecid. Crmp2 is largely restricted to the nervous system (Goshima et al., 1995; Wang and Strittmatter, 1996; Charrier et al., 2003), and acts as a microtubule-stabilizer (Gu and Ihara, 2000; Fukata et al., 2002; Lin et al., 2011) in part via promoting α/β-tubulin dimer assembly onto growing microtubule plus-ends (Niwa et al., 2017), thereby playing a key role in promoting neurite outgrowth (e.g., Suzuki et al., 2003; Crews et al., 2011; Higurashi et al., 2012; Wilson et al., 2014; reviewed in Ip et al., 2014; Quach et al., 2015).

We observed a postnatal decline in Panx1 expression in the VZ, reminiscent of the decrease in Panx1 levels across postnatal neuronal differentiation and neuritogenesis that we previously observed *in vitro* (Wicki-Stordeur and Swayne, 2013). This time course of reduction in Panx1 expression levels corresponds with critical periods of increased neuritogenesis, neurite stabilization, and network development, which roughly occur across similar postnatal time-courses in the developing cortex, cerebellum and eye (Heng et al., 2010; Kato et al., 2012). Given our recent findings demonstrating a key role for Panx1 in NPC maintenance *in vivo* (Wicki-Stordeur et al., 2016), this decrease in expression could also be associated with the age-dependent decrease in the number of VZ NPCs observed in mice and humans (Sanai et al., 2011; Wang et al., 2011; Shook et al., 2012; Daynac et al., 2016; reviewed in Apple et al., 2017; Conover and Todd, 2017).

In our earlier study using probenecid, we observed a remarkable induction of neurite outgrowth in both N2a cells and primary VZ NPC cultures (Wicki-Stordeur and Swayne, 2013). Because Crmp2 is a microtubule-stabilizer, we wondered whether this effect of probenecid could be due to modulation of the Panx1-Crmp2 interaction. Since probenecid is a small molecule with a hydrophobic core, it is reasonable for us to suspect that it readily crosses cell membranes disrupting both plasma membrane and intracellular Panx1-Crmp2 interactions. Remarkably, probenecid exposure indeed decreased the amount of Crmp2 co-precipitating with Panx1, without affecting total Crmp2 expression (Figure 3). Taken together with our data demonstrating a direct interaction between the purified Panx1CT and Crmp2 (Figure 1), these results suggest that binding of probenecid at the first extracellular loop (Michalski and Kawate, 2016) allosterically disrupts the Crmp2 interaction at the Panx1CT. Notably, the Panx1CT has been reported to be involved in blocking the Panx1 pore (Chekeni et al., 2010; Sandilos et al., 2012; Dourado et al., 2014; Chiu et al., 2017), therefore it is tempting to speculate based on our findings, that its stimulation of allosteric communication between the EL1 and the C-terminus (CT) could underlie probenecid-mediated Panx1 inhibition.

Complementary PLA confirmed a decrease in the Panx1-Crmp2 association in the presence of probenecid. It is important to note that the intensity of the PLA signal does not reflect the actual number of interacting molecules, and a large signal can be generated by a very small number of interacting molecules. This is because the PLA signal is generated by a process called rolling circle DNA synthesis, which occurs when oligonucleotide probes connected to the secondary antibodies towards each primary antibody are in close proximity or part of a protein complex; the reaction results in several hundred-fold amplification of the DNA circle.

Immunocytochemistry further revealed that Crmp2 was concentrated at the tips of neurites in probenecid treated cells. This is consistent with another study demonstrating concentration of Crmp2 on the distal region of growing axons in primary hippocampal neurons (Inagaki et al., 2001). Interestingly, we noted that Crmp2 signal in the neurite tip vs. shaft was significantly higher in short neurites (10–30 μm) compared to the longer ones (30–50 μm and >50 μm). Assuming that the shorter neurites are at an earlier stage of formation, this finding suggests that Crmp2 may be more heavily involved in microtubule assembly during the initial stages of neurite formation.

**Figure 7:**
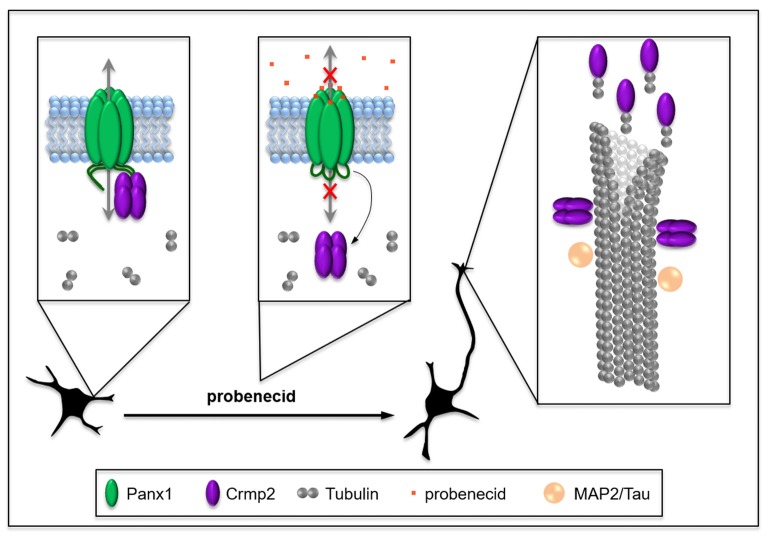
Working model: probenecid promotes neurite outgrowth by releasing Crmp2 thereby stabilizing microtubules. Panx1, which is developmentally regulated at a cellular and tissue level in neural precursor cells (NPCs), physically sequesters Crmp2, thereby inhibiting neuritogenesis until appropriate connections can be made. Probenecid binds to the first extracellular loop causing allosteric disruption of Crmp2 binding to the CT and resulting in a “release” of Crmp2 from the Panx1/Crmp2 complex. Free Crmp2 facilitates tubulin polymerization, and microtubule stabilization and bundling, thereby promoting neurite extension. MAP2 and Tau are microtubule-associated proteins that also contribute to microtubule stabilization in different types of neurites in the CNS and are included for context.

It is reasonable to hypothesize that this increase in free Crmp2 would impact on microtubule stability. Supporting this prediction, probenecid also significantly increased microtubule polymerization, as well as post-translational modifications associated with microtubule stability. Because of Crmp2’s well-documented role in stabilizing microtubules through binding to microtubule-plus ends (Niwa et al., 2017), these novel findings by our group suggest that probenecid-mediated neurite outgrowth could result from release of Crmp2 from Panx1. Importantly, our *in vitro* microtubule polymerization assay results support the idea that probenecid’s enhancement of microtubule stability in cells occurs though an indirect mechanism. Figure 7 demonstrates our working model, in which probenecid binding to Panx1 results in release of Crmp2, which then concentrates at microtubule plus-ends to promote neurite stability and elongation.

Our discovery of the interaction between Panx1 and Crmp2 expands our understanding of Panx1 interactions with the cytoskeleton. Bhalla-Gehi et al. (2010) first reported an interaction between actin and ectopically expressed Panx1, and demonstrated that actin filament (not microtubule) dynamics were critical for Panx1 trafficking and stability at the plasma membrane. We also previously identified several cytoskeletal proteins as potential Panx1 interaction partners, and validated an interaction between endogenous Panx1, actin and Arp3, a component of the actin-associated Arp2/3 complex, in N2a cells (Wicki-Stordeur and Swayne, 2013). Our novel data presented here additionally links Panx1 to the microtubule cytoskeleton. Notably, in addition to regulating microtubule stability, Crmp2 also acts as a bridge between microtubule and actin networks (Tan et al., 2015). Therefore, the potential role of Panx1 in regulating microtubule and actin filament crosstalk during neuritogenesis will be the focus of future work.

Overall this work identifies a new protein-protein interaction implicated in regulation of the cytoskeleton. These data are especially relevant in the context of neuroplasticity, with important implications for crosstalk between ion and metabolite channels like Panx1 and the cytoskeleton.

## Author Contributions

LEW-S, XX, JCS-A and LAS designed research and wrote the manuscript. LEW-S, XX, JCS-A, ML, MSW and CC performed research. LEW-S, XX, JCS-A, ML, MSW, CSWC and LAS analyzed data.

## Conflict of Interest Statement

The authors declare that the research was conducted in the absence of any commercial or financial relationships that could be construed as a potential conflict of interest.

## Footnotes

^1^http://imagej.nih.gov/ij/

^2^https://imagej.nih.gov/ij/

^3^https://imagej.net/Fiji

^4^http://www.graphpad.com
