# Supplementary material for: Probenecid Disrupts a Novel Pannexin 1-Collapsin Response Mediator Protein 2 Interaction and Increases Microtubule Stability
Source: Front Cell Neurosci. 2018 May 11;12:124. doi: 10.3389/fncel.2018.00124 (PMC5958195; doi:10.3389/fncel.2018.00124)

Western Blots – Panx1 and CRMP2 expression levels in VZ

CRMP2 Immunoreactivity (IR)

```
GET DATA
  /TYPE=XLSX
  /FILE='Z:\Swayne Lab\Manuscripts\2018 Frontiers Panx1-Crmp2
paper(dropbox)\Deep statistical analysis\SVZ Expression\CRMP2_SVZ_IR.xlsx'
  /SHEET=name 'CRMP2_SVZ_IR'
  /CELLRANGE=FULL
  /READNAMES=ON
  /DATATYPEMIN PERCENTAGE=95.0
  /HIDDEN IGNORE=YES.
EXECUTE.
DATASET NAME DataSet3 WINDOW=FRONT.
EXAMINE VARIABLES=CRMP2_IR
  /PLOT HISTOGRAM NPPLOT
  /STATISTICS DESCRIPTIVES
  /CINTERVAL 95
  /MISSING LISTWISE
  /NOTOTAL.
```

Explore

Notes

Output Created		14-MAR-2018 10:25:54
Comments		
Input	Active Dataset	DataSet3
	Filter	<none>
	Weight	<none>
	Split File	<none>
	N of Rows in Working Data File	15
Missing Value Handling	Definition of Missing	User-defined missing values for dependent variables are treated as missing.

	Cases Used	Statistics are based on cases with no missing values for any dependent variable or factor used.
Syntax		EXAMINE VARIABLES=CRMP2_IR<br>/PLOT HISTOGRAM NPPLOT<br>/STATISTICS DESCRIPTIVES<br>/CINTERVAL 95<br>/MISSING LISTWISE<br>/NOTOTAL.
Resources	Processor Time	00:00:00.33
	Elapsed Time	00:00:00.48

[DataSet3]

Case Processing Summary

	Cases					
	Valid		Missing		Total	
	N	Percent	N	Percent	N	Percent
CRMP2_IR	15	100.0%	0	0.0%	15	100.0%

Descriptives

			Statistic	Std. Error
CRMP2_IR	Mean		93.3938308	4.97066009
	95% Confidence Interval for Mean	Lower Bound	82.7328252	
		Upper Bound	104.0548364	
	5% Trimmed Mean		93.7074298	
	Median		97.3076208	
	Variance		370.612	
	Std. Deviation		19.25128375	
	Minimum		60.42418	
	Maximum		120.71870	

	Range	60.29452	
	Interquartile Range	37.65426	
	Skewness	-.311	.580
	Kurtosis	-1.145	1.121

Tests of Normality

	Kolmogorov-Smirnov[a]			Shapiro-Wilk		
	Statistic	df	Sig.	Statistic	df	Sig.
CRMP2_IR	.195	15	.129	.922	15	.209

a. Lilliefors Significance Correction

CRMP2_IR

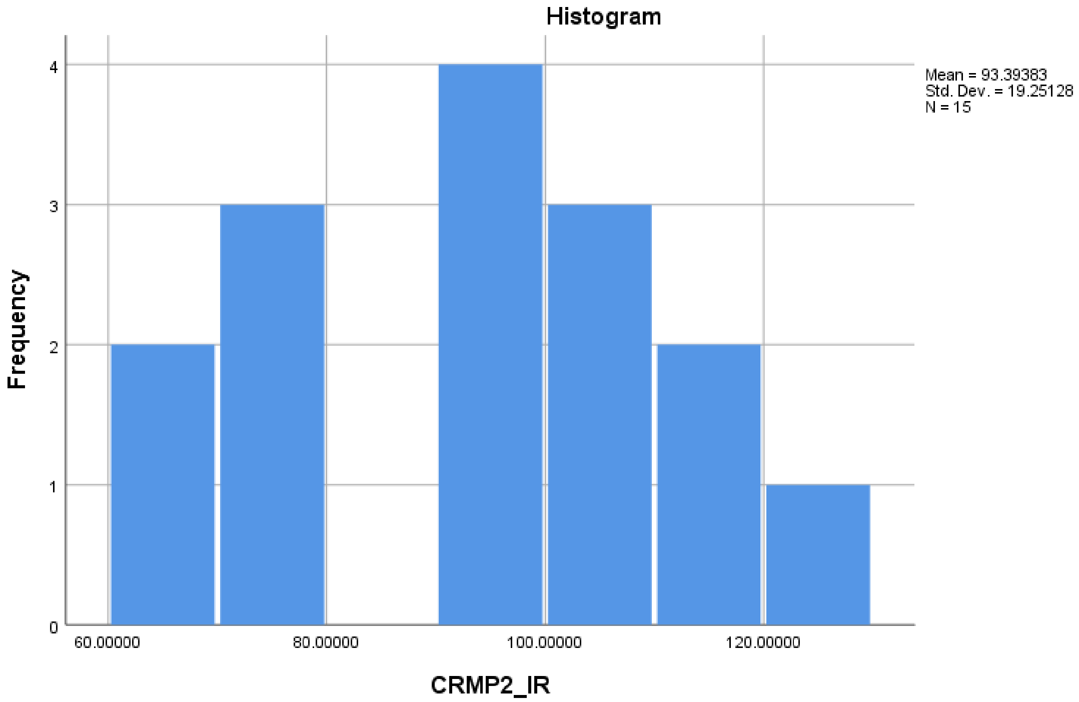

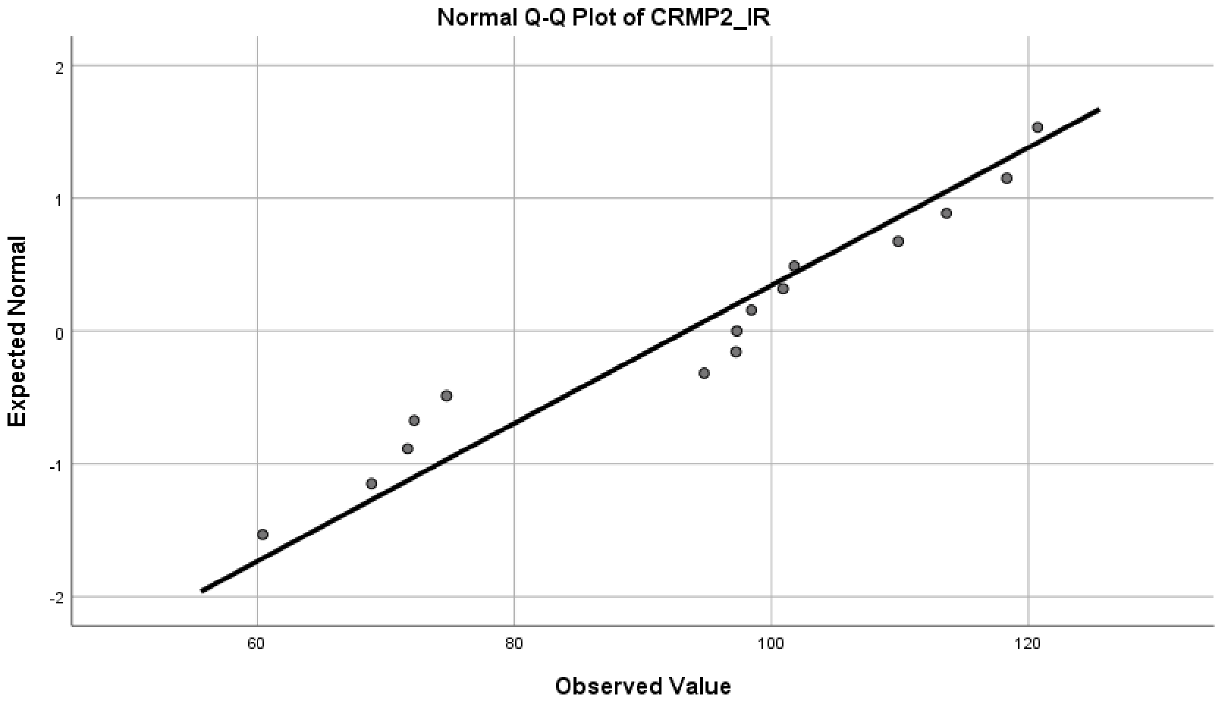

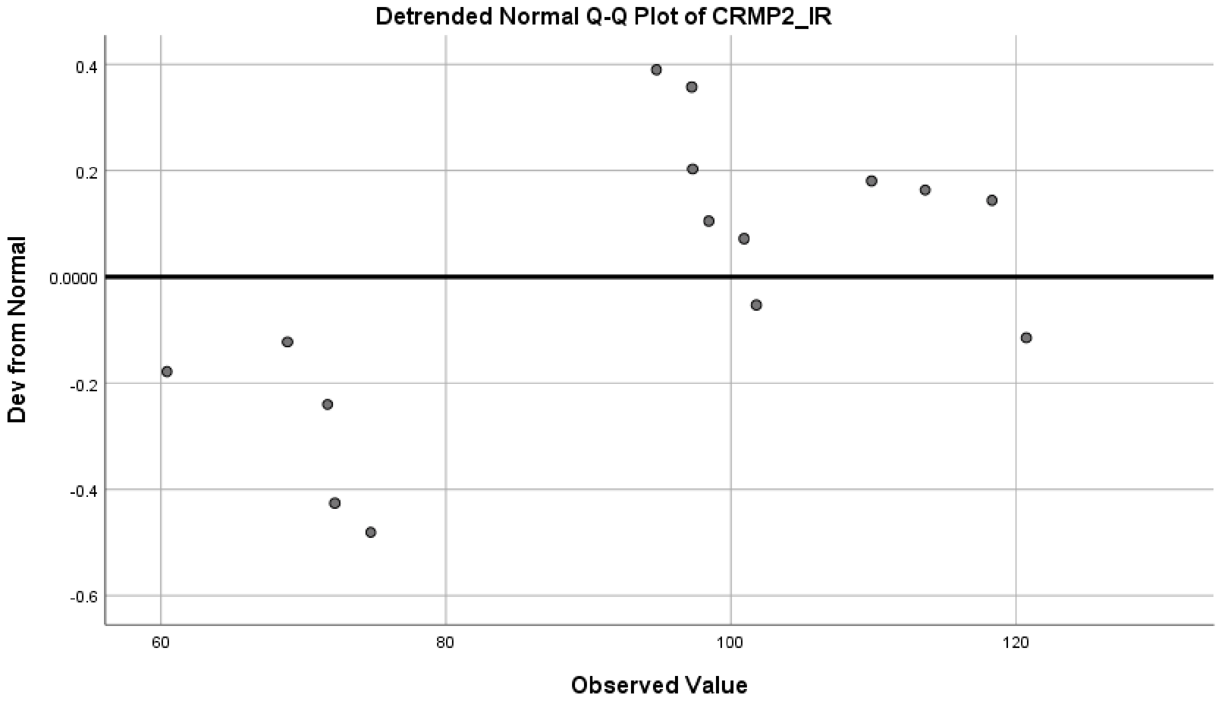


```
ONEWAY CRMP2_IR BY Age
  /MISSING ANALYSIS
```

```
/POSTHOC=DUNNETT (1) ALPHA(0.05).
```

Oneway

Notes

Output Created		14-MAR-2018 10:26:16
Comments		
Input	Active Dataset	DataSet3
	Filter	<none>
	Weight	<none>
	Split File	<none>
	N of Rows in Working Data File	15
Missing Value Handling	Definition of Missing	User-defined missing values are treated as missing.
	Cases Used	Statistics for each analysis are based on cases with no missing data for any variable in the analysis.
Syntax		ONEWAY CRMP2_IR BY Age /MISSING ANALYSIS /POSTHOC=DUNNETT (1) ALPHA(0.05).
Resources	Processor Time	00:00:00.09
	Elapsed Time	00:00:00.10

ANOVA

CRMP2_IR

	Sum of Squares	df	Mean Square	F	Sig.
Between Groups	1777.036	4	444.259	1.302	.333
Within Groups	3411.531	10	341.153		

Total	5188.567	14			

Post Hoc Tests

Multiple Comparisons

Dependent Variable: CRMP2_IR

Dunnett t (2-sided)[a]

(I) Age	(J) Age	Mean Difference (I-J)	Std. Error	Sig.	95% Confidence Interval Lower Bound	Upper Bound
2	1	-10.70087787	15.08096083	.884	-54.2920967	32.8903410
3	1	-18.33161681	15.08096083	.581	-61.9228356	25.2596020
4	1	-15.26572044	15.08096083	.711	-58.8569393	28.3254984
5	1	11.26736889	15.08096083	.866	-32.3238499	54.8585877

a. Dunnett t-tests treat one group as a control, and compare all other groups against it.

```
UNIANOVA CRMP2_IR BY Age
  /METHOD=SSTYPE(3)
  /INTERCEPT=INCLUDE
  /SAVE=ZRESID
  /PRINT DESCRIPTIVE HOMOGENEITY
  /CRITERIA=ALPHA(.05)
  /DESIGN=Age.
```

Univariate Analysis of Variance

Notes

Output Created		14-MAR-2018 10:26:37
Comments		
Input	Active Dataset	DataSet3
	Filter	<none>

	Weight	<none>
	Split File	<none>
	N of Rows in Working Data File	15
Missing Value Handling	Definition of Missing	User-defined missing values are treated as missing.
	Cases Used	Statistics are based on all cases with valid data for all variables in the model.
Syntax		UNIANOVA CRMP2_IR BY Age /METHOD=SSTYPE(3) /INTERCEPT=INCLUDE /SAVE=ZRESID /PRINT DESCRIPTIVE HOMOGENEITY /CRITERIA=ALPHA(.05) /DESIGN=Age.
Resources	Processor Time	00:00:00.00
	Elapsed Time	00:00:00.07
Variables Created or Modified	ZRE_1	Standardized Residual for CRMP2_IR

Between-Subjects Factors

		N
Age	1	3
	2	3
	3	3
	4	3
	5	3

Descriptive Statistics

Dependent Variable: CRMP2_IR

Age	Mean	Std. Deviation	N
1	100.0000000	2.37186602	3

2	89.2991222	14.81596298	3
3	81.6683832	25.43923710	3
4	84.7342796	25.05824072	3
5	111.2673689	14.33724613	3
Total	93.3938308	19.25128375	15

Levene's Test of Equality of Error Variances[a,b]

		Levene Statistic	df1	df2	Sig.
CRMP2_IR	Based on Mean	3.198	4	10	.062
	Based on Median	.432	4	10	.783
	Based on Median and with adjusted df	.432	4	6.500	.782
	Based on trimmed mean	2.766	4	10	.087

Tests the null hypothesis that the error variance of the dependent variable is equal across groups.[a,b]

a. Dependent variable: CRMP2_IR

b. Design: Intercept + Age

Tests of Between-Subjects Effects

Dependent Variable: CRMP2_IR

Source	Type III Sum of Squares	df	Mean Square	F	Sig.
Corrected Model	1777.036[a]	4	444.259	1.302	.333
Intercept	130836.114	1	130836.114	383.511	.000
Age	1777.036	4	444.259	1.302	.333
Error	3411.531	10	341.153		
Total	136024.681	15			
Corrected Total	5188.567	14			

a. R Squared = .342 (Adjusted R Squared = .079)

```
EXAMINE VARIABLES=ZRE_1
  /PLOT HISTOGRAM NPPLOT
  /STATISTICS DESCRIPTIVES
  /CINTERVAL 95
  /MISSING LISTWISE
  /NOTOTAL.
```

Explore

Notes

Output Created		14-MAR-2018 10:26:55
Comments		
Input	Active Dataset	DataSet3
	Filter	<none>
	Weight	<none>
	Split File	<none>
	N of Rows in Working Data File	15
Missing Value Handling	Definition of Missing	User-defined missing values for dependent variables are treated as missing.
	Cases Used	Statistics are based on cases with no missing values for any dependent variable or factor used.
Syntax		EXAMINE VARIABLES=ZRE_1<br>/PLOT HISTOGRAM NPPLOT<br>/STATISTICS DESCRIPTIVES<br>/CINTERVAL 95<br>/MISSING LISTWISE<br>/NOTOTAL.
Resources	Processor Time	00:00:00.37
	Elapsed Time	00:00:00.29

Case Processing Summary

Cases

	Valid		Missing		Total	
	N	Percent	N	Percent	N	Percent
Standardized Residual for CRMP2_IR	15	100.0%	0	0.0%	15	100.0%

Descriptives

			Statistic	Std. Error
Standardized Residual for CRMP2_IR	Mean		.0000	.21822
	95% Confidence Interval for Mean	Lower Bound	-.4680	
		Upper Bound	.4680	
	5% Trimmed Mean		-.0230	
	Median		.0493	
	Variance		.714	
	Std. Deviation		.84515	
	Minimum		-1.15	
	Maximum		1.56	
	Range		2.71	
	Interquartile Range		1.35	
	Skewness		.506	.580
	Kurtosis		-.425	1.121

Tests of Normality

	Kolmogorov-Smirnov[a]			Shapiro-Wilk		
	Statistic	df	Sig.	Statistic	df	Sig.
Standardized Residual for CRMP2_IR	.139	15	.200*	.927	15	.245

*. This is a lower bound of the true significance.

a. Lilliefors Significance Correction

Standardized Residual for CRMP2_IR

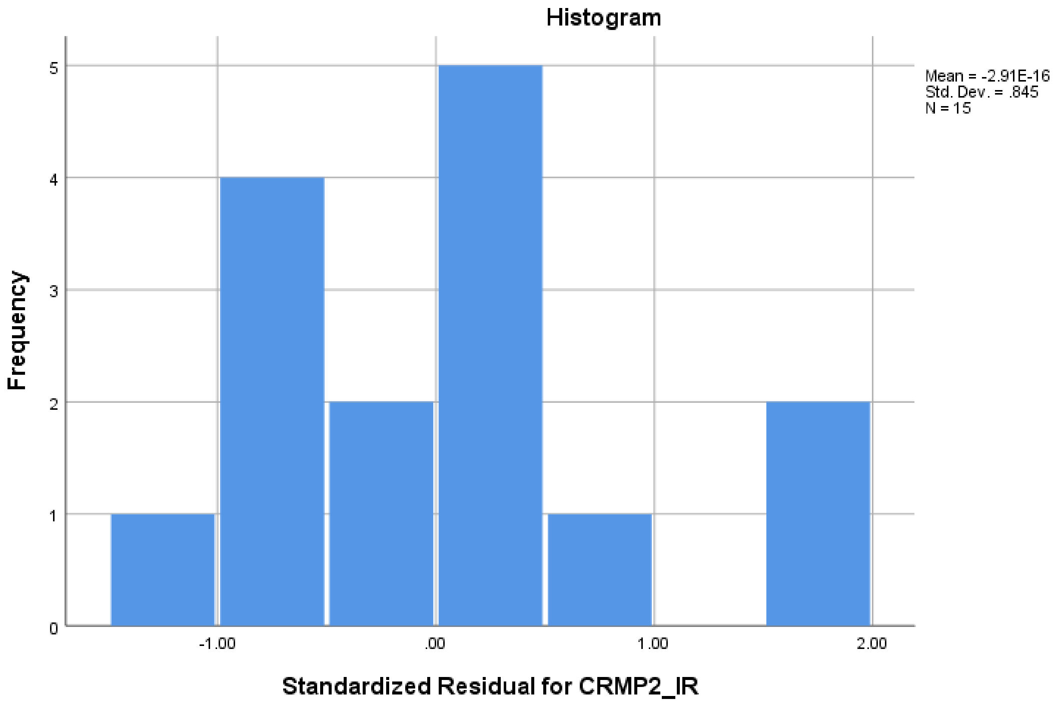
Histogram
Mean = -2.91E-16
Std. Dev. = .845
N = 15
Frequency
Standardized Residual for CRMP2_IR

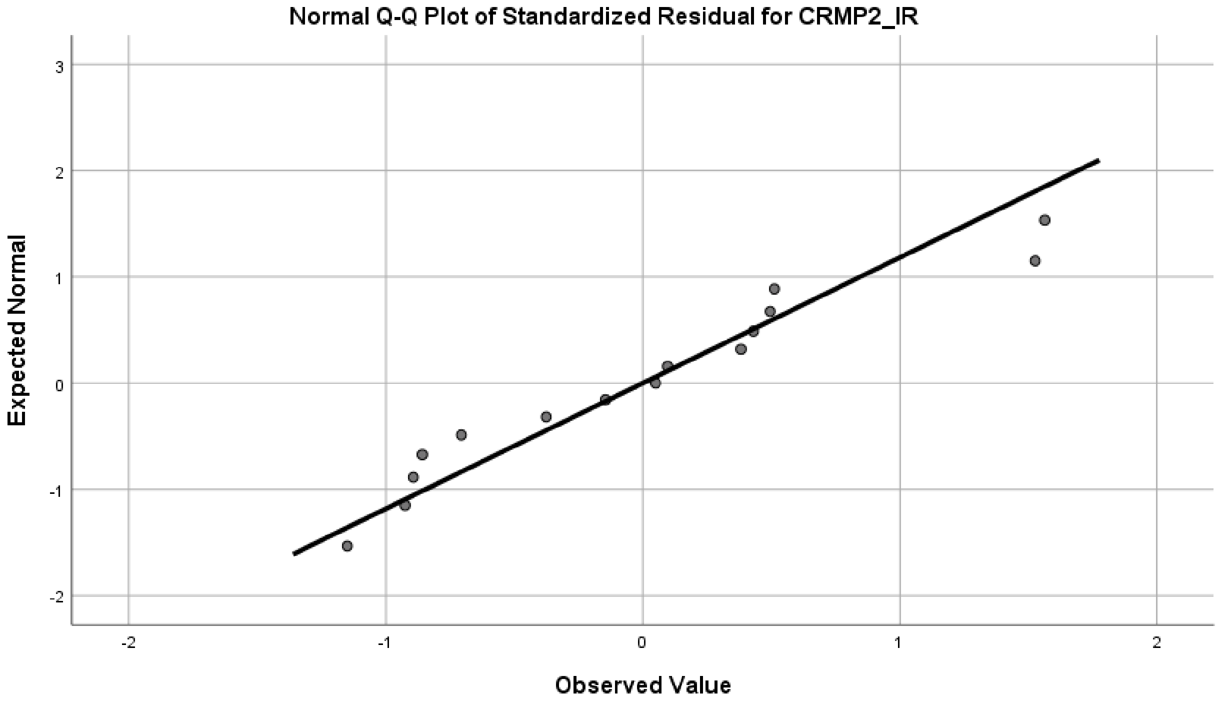
Normal Q-Q Plot of Standardized Residual for CRMP2_IR
Expected Normal
Observed Value

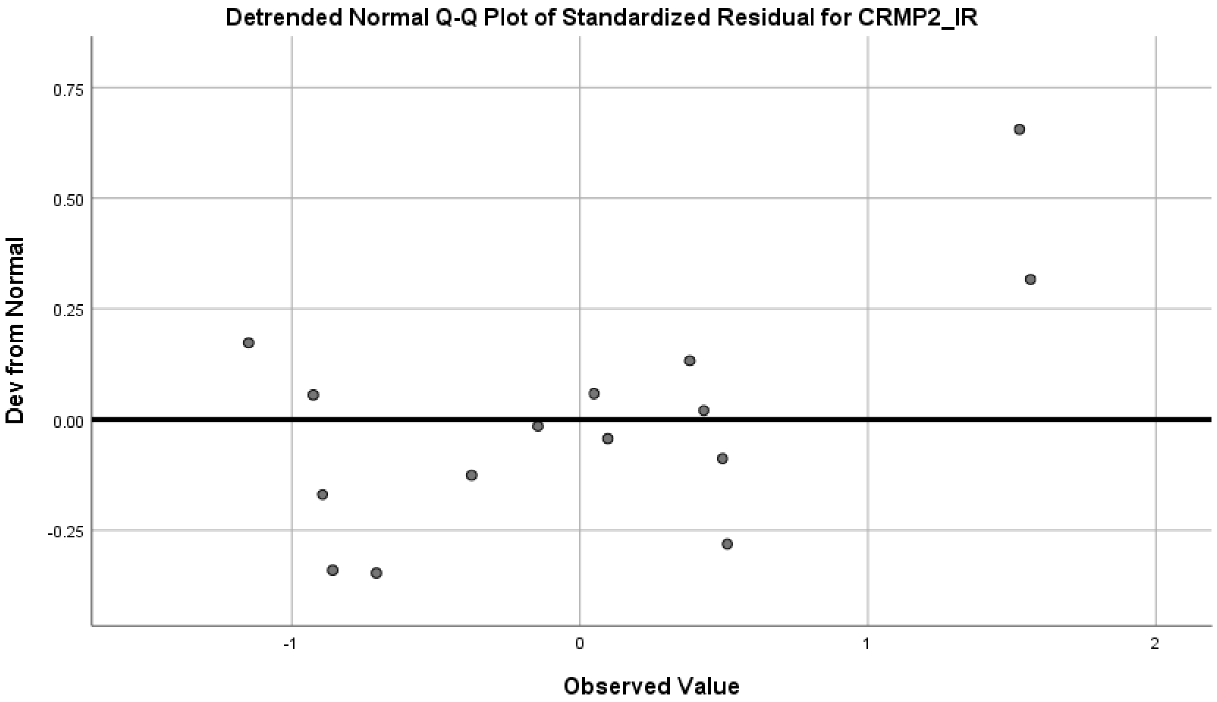

Panx1 IR

```
GET DATA
  /TYPE=XLSX
  /FILE='Z:\Swayne Lab\Manuscripts\2018 Frontiers Panx1-Crmp2
paper(dropbox)\Deep statistical analysis\SVZ Expression\Panx1_SVZ_IR.xlsx'
  /SHEET=name 'Panx1_SVZ_IR'
  /CELLRANGE=FULL
  /READNAMES=ON
  /DATATYPEMIN PERCENTAGE=95.0
  /HIDDEN IGNORE=YES.
EXECUTE.
DATASET NAME DataSet1 WINDOW=FRONT.
EXAMINE VARIABLES=Panx1_IR
  /PLOT HISTOGRAM NPPLOT
  /STATISTICS DESCRIPTIVES
  /CINTERVAL 95
  /MISSING LISTWISE
  /NOTOTAL.
```

Explore

Notes

Output Created		14-MAR-2018 10:21:59
Comments		
Input	Active Dataset	DataSet1
	Filter	<none>
	Weight	<none>
	Split File	<none>
	N of Rows in Working Data File	15
Missing Value Handling	Definition of Missing	User-defined missing values for dependent variables are treated as missing.
	Cases Used	Statistics are based on cases with no missing values for any dependent variable or factor used.
Syntax		EXAMINE VARIABLES=Panx1_IR<br>/PLOT HISTOGRAM NPPLOT<br>/STATISTICS DESCRIPTIVES<br>/CINTERVAL 95<br>/MISSING LISTWISE<br>/NOTOTAL.
Resources	Processor Time	00:00:01.03
	Elapsed Time	00:00:01.40

```
[DataSet1]
```

Case Processing Summary

Cases					
Valid		Missing		Total	
N	Percent	N	Percent	N	Percent

Panx1_IR	15	100.0%	0	0.0%	15	100.0%

Descriptives

			Statistic	Std. Error
Panx1_IR	Mean		63.8592121	7.54641665
	95% Confidence Interval for Mean	Lower Bound	47.6737581	
		Upper Bound	80.0446661	
	5% Trimmed Mean		63.0100300	
	Median		57.2457974	
	Variance		854.226	
	Std. Deviation		29.22714601	
	Minimum		26.61599	
	Maximum		116.38771	
	Range		89.77173	
	Interquartile Range		47.23517	
	Skewness		.470	.580
	Kurtosis		-.783	1.121

Tests of Normality

	Kolmogorov-Smirnov[a]			Shapiro-Wilk		
	Statistic	df	Sig.	Statistic	df	Sig.
Panx1_IR	.123	15	.200*	.934	15	.318

*. This is a lower bound of the true significance.

a. Lilliefors Significance Correction

Panx1_IR

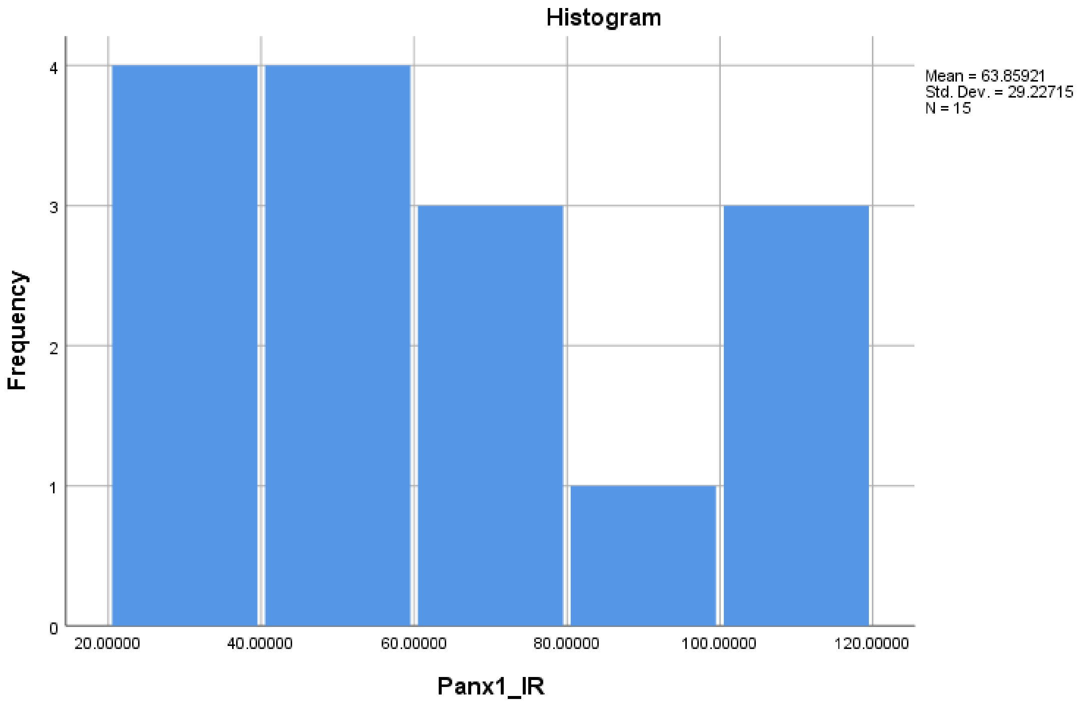
Histogram
Mean = 63.85921
Std. Dev. = 29.22715
N = 15
Frequency
0
1
2
3
4
20.00000
40.00000
60.00000
80.00000
100.00000
120.00000
Panx1_IR

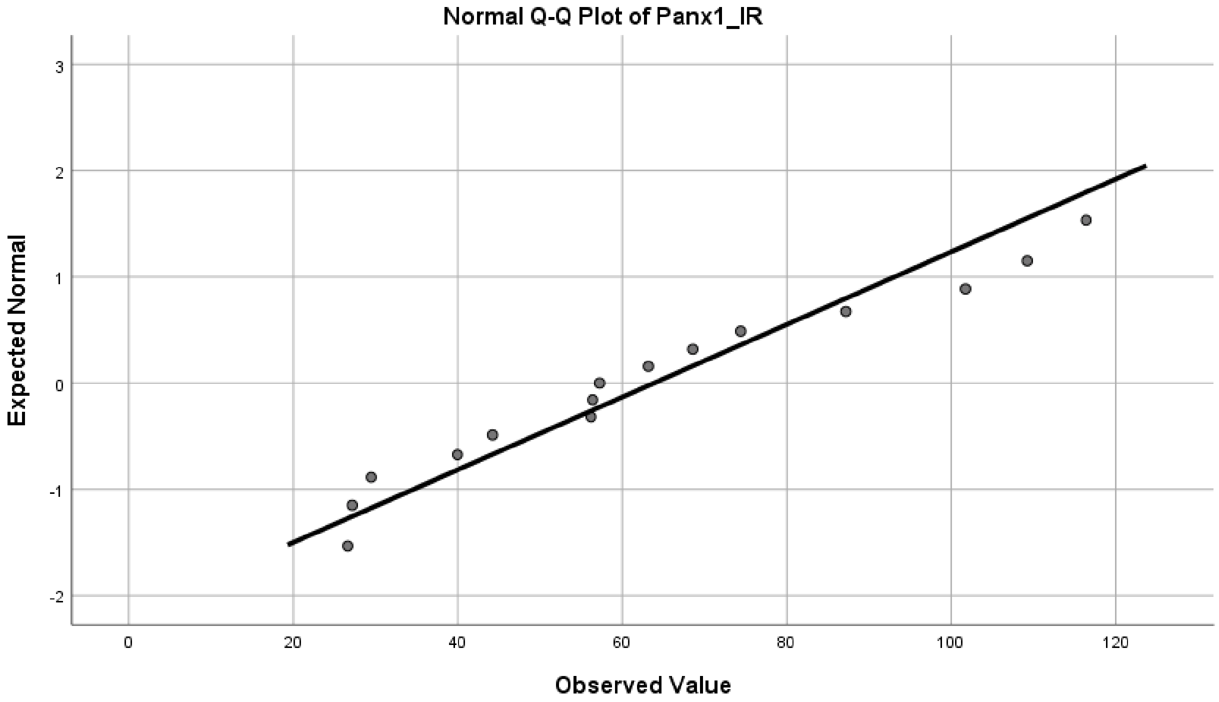
Normal Q-Q Plot of Panx1_IR
Expected Normal
3
2
1
0
-1
-2
0
20
40
60
80
100
120
Observed Value

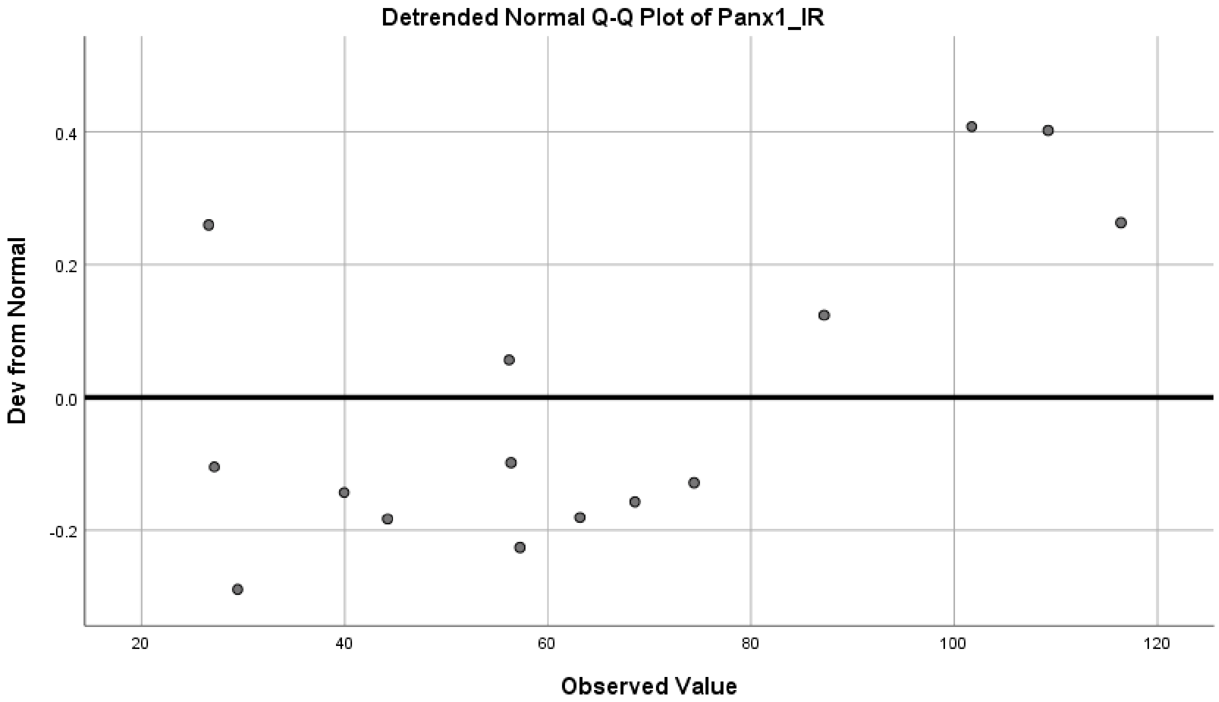


```
ONEWAY Panx1_IR BY Age
  /STATISTICS DESCRIPTIVES HOMOGENEITY
  /MISSING ANALYSIS
  /POSTHOC=DUNNETT (1) ALPHA(0.05).
```

Oneway

Notes

Output Created		14-MAR-2018 10:22:37
Comments		
Input	Active Dataset	DataSet1
	Filter	<none>
	Weight	<none>
	Split File	<none>
	N of Rows in Working Data File	15

Missing Value Handling	Definition of Missing	User-defined missing values are treated as missing.
	Cases Used	Statistics for each analysis are based on cases with no missing data for any variable in the analysis.
Syntax		ONEWAY Panx1_IR BY Age<br>/STATISTICS DESCRIPTIVES HOMOGENEITY<br>/MISSING ANALYSIS<br>/POSTHOC=DUNNETT (1) ALPHA(0.05).
Resources	Processor Time	00:00:00.11
	Elapsed Time	00:00:00.15

Descriptives

Panx1_IR

	N	Mean	Std. Deviation	Std. Error	95% Confidence Interval for Mean	
					Lower Bound	Upper Bound
1	3	100.0000000	22.47170487	12.97404486	44.1771905	155.8228095
2	3	73.6894386	24.51624980	14.15446342	12.7876979	134.5911793
3	3	62.5974477	22.14846166	12.78742030	7.5776188	117.6172766
4	3	45.2318960	21.19577817	12.23738823	-7.4213359	97.8851278
5	3	37.7772782	16.92051571	9.76906430	-4.2556130	79.8101693
Total	15	63.8592121	29.22714601	7.54641665	47.6737581	80.0446661

Descriptives

Panx1_IR

	Minimum	Maximum
1	74.38318	116.38771
2	56.19179	101.71054
3	44.21935	87.18905
4	27.17386	68.56796
5	26.61599	57.24580
Total	26.61599	116.38771

Test of Homogeneity of Variances

		Levene Statistic	df1	df2	Sig.
Panx1_IR	Based on Mean	.189	4	10	.939
	Based on Median	.038	4	10	.997
	Based on Median and with adjusted df	.038	4	9.273	.997
	Based on trimmed mean	.168	4	10	.950

ANOVA

Panx1_IR

	Sum of Squares	df	Mean Square	F	Sig.
Between Groups	7294.878	4	1823.720	3.910	.037
Within Groups	4664.286	10	466.429		
Total	11959.165	14			

Post Hoc Tests

Multiple Comparisons

Dependent Variable: Panx1_IR

Dunnett t (2-sided)[a]

					95% Confidence Interval	
(I) Age	(J) Age	Mean Difference (I-J)	Std. Error	Sig.	Lower Bound	Upper Bound
2	1	-26.31056139	17.63384338	.419	-77.2808367	24.6597139
3	1	-37.40255231	17.63384338	.171	-88.3728276	13.5677230
4	1	-54.76810405*	17.63384338	.035	-105.7383794	-3.7978287
5	1	-62.22272184*	17.63384338	.018	-113.1929972	-11.2524465

*. The mean difference is significant at the 0.05 level.

a. Dunnett t-tests treat one group as a control, and compare all other groups against it.

```
UNIANOVA Panx1_IR BY Age
  /METHOD=SSTYPE(3)
  /INTERCEPT=INCLUDE
```

```
/SAVE=ZRESID
/PRINT DESCRIPTIVE HOMOGENEITY
/CRITERIA=ALPHA(.05)
/DESIGN=Age.
```

Univariate Analysis of Variance

Notes

Output Created		14-MAR-2018 10:23:21
Comments		
Input	Active Dataset	DataSet1
	Filter	<none>
	Weight	<none>
	Split File	<none>
	N of Rows in Working Data File	15
Missing Value Handling	Definition of Missing	User-defined missing values are treated as missing.
	Cases Used	Statistics are based on all cases with valid data for all variables in the model.
Syntax		UNIANOVA Panx1_IR BY Age /METHOD=SSTYPE(3) /INTERCEPT=INCLUDE /SAVE=ZRESID /PRINT DESCRIPTIVE HOMOGENEITY /CRITERIA=ALPHA(.05) /DESIGN=Age.
Resources	Processor Time	00:00:00.00
	Elapsed Time	00:00:00.21
Variables Created or Modified	ZRE_1	Standardized Residual for Panx1_IR

Between-Subjects Factors

		N
Age	1	3
	2	3
	3	3
	4	3
	5	3

Descriptive Statistics

Dependent Variable: Panx1_IR

Age	Mean	Std. Deviation	N
1	100.0000000	22.47170487	3
2	73.6894386	24.51624980	3
3	62.5974477	22.14846166	3
4	45.2318960	21.19577817	3
5	37.7772782	16.92051571	3
Total	63.8592121	29.22714601	15

Levene's Test of Equality of Error Variances[a,b]

		Levene Statistic	df1	df2	Sig.
Panx1_IR	Based on Mean	.189	4	10	.939
	Based on Median	.038	4	10	.997
	Based on Median and with adjusted df	.038	4	9.273	.997
	Based on trimmed mean	.168	4	10	.950

Tests the null hypothesis that the error variance of the dependent variable is equal across groups.[a,b]

a. Dependent variable: Panx1_IR

b. Design: Intercept + Age

Tests of Between-Subjects Effects

Dependent Variable: Panx1_IR

Source	Type III Sum of Squares	df	Mean Square	F	Sig.
Corrected Model	7294.878[a]	4	1823.720	3.910	.037
Intercept	61169.985	1	61169.985	131.145	.000
Age	7294.878	4	1823.720	3.910	.037
Error	4664.286	10	466.429		
Total	73129.149	15			
Corrected Total	11959.165	14			

a. R Squared = .610 (Adjusted R Squared = .454)

```
EXAMINE VARIABLES=ZRE_1
  /PLOT HISTOGRAM NPPLOT
  /STATISTICS DESCRIPTIVES
  /CINTERVAL 95
  /MISSING LISTWISE
  /NOTOTAL.
```

Explore

Notes

Output Created		14-MAR-2018 10:23:34
Comments		
Input	Active Dataset	DataSet1
	Filter	<none>
	Weight	<none>
	Split File	<none>
	N of Rows in Working Data File	15
Missing Value Handling	Definition of Missing	User-defined missing values for dependent variables are treated as missing.

	Cases Used	Statistics are based on cases with no missing values for any dependent variable or factor used.
Syntax		EXAMINE VARIABLES=ZRE_1<br>/PLOT HISTOGRAM NPPLOT<br>/STATISTICS DESCRIPTIVES<br>/CINTERVAL 95<br>/MISSING LISTWISE<br>/NOTOTAL.
Resources	Processor Time	00:00:00.36
	Elapsed Time	00:00:00.29

Case Processing Summary

	Cases					
	Valid		Missing		Total	
	N	Percent	N	Percent	N	Percent
Standardized Residual for Panx1_IR	15	100.0%	0	0.0%	15	100.0%

Descriptives

			Statistic	Std. Error
Standardized Residual for Panx1_IR	Mean		.0000	.21822
	95% Confidence Interval for Mean	Lower Bound	-.4680	
		Upper Bound	.4680	
	5% Trimmed Mean		-.0062	
	Median		-.2877	
	Variance		.714	
	Std. Deviation		.84515	
	Minimum		-1.19	
	Maximum		1.30	
	Range		2.48	
	Interquartile Range		1.71	

	Skewness	.330	.580
	Kurtosis	-1.511	1.121

Tests of Normality

	Kolmogorov-Smirnov[a]			Shapiro-Wilk		
	Statistic	df	Sig.	Statistic	df	Sig.
Standardized Residual for Panx1_IR	.214	15	.064	.897	15	.087

a. Lilliefors Significance Correction

Standardized Residual for Panx1_IR

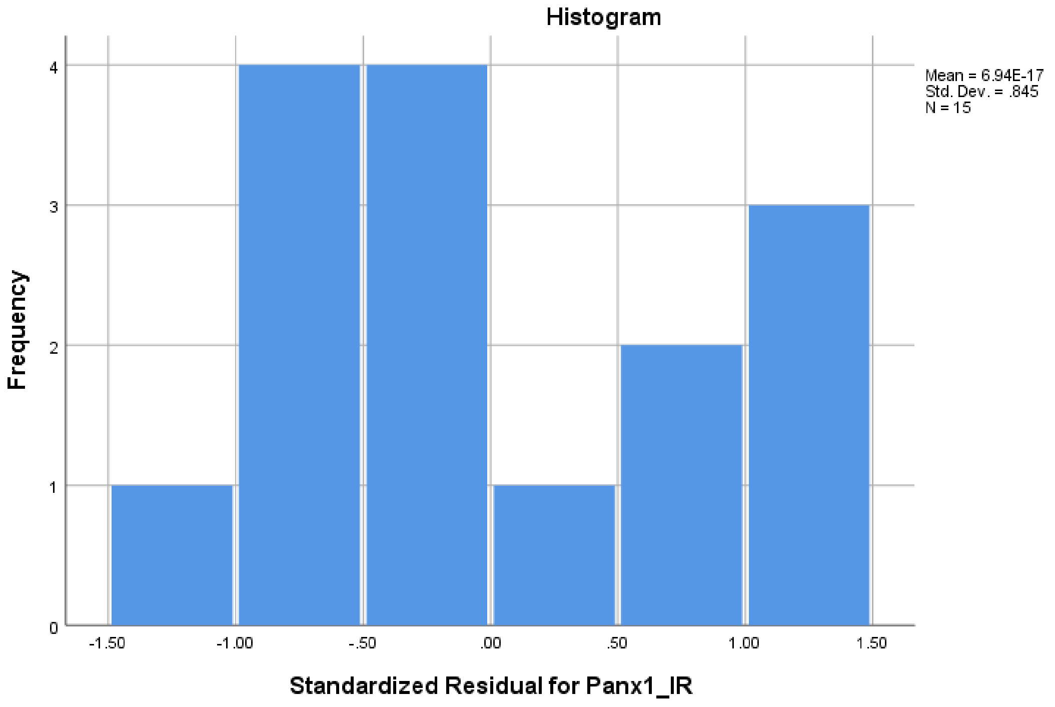

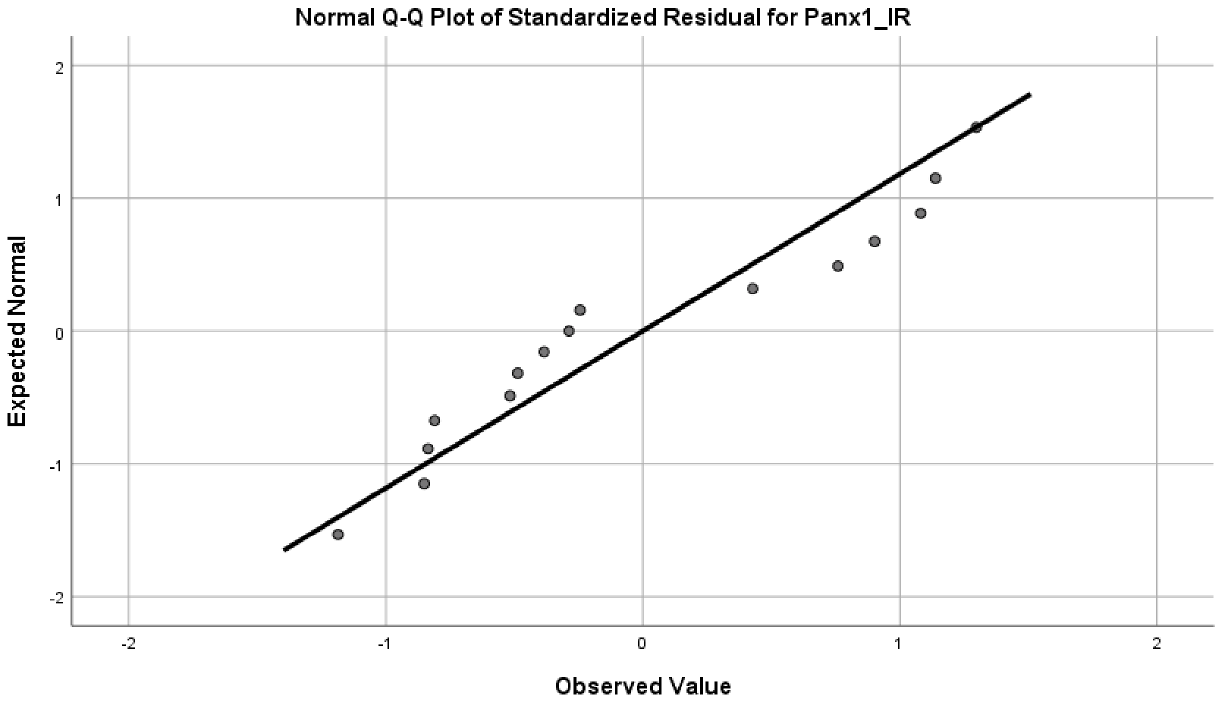

Normal Q-Q Plot of Standardized Residual for Panx1_IR
Expected Normal
Observed Value
2
1
0
-1
-2

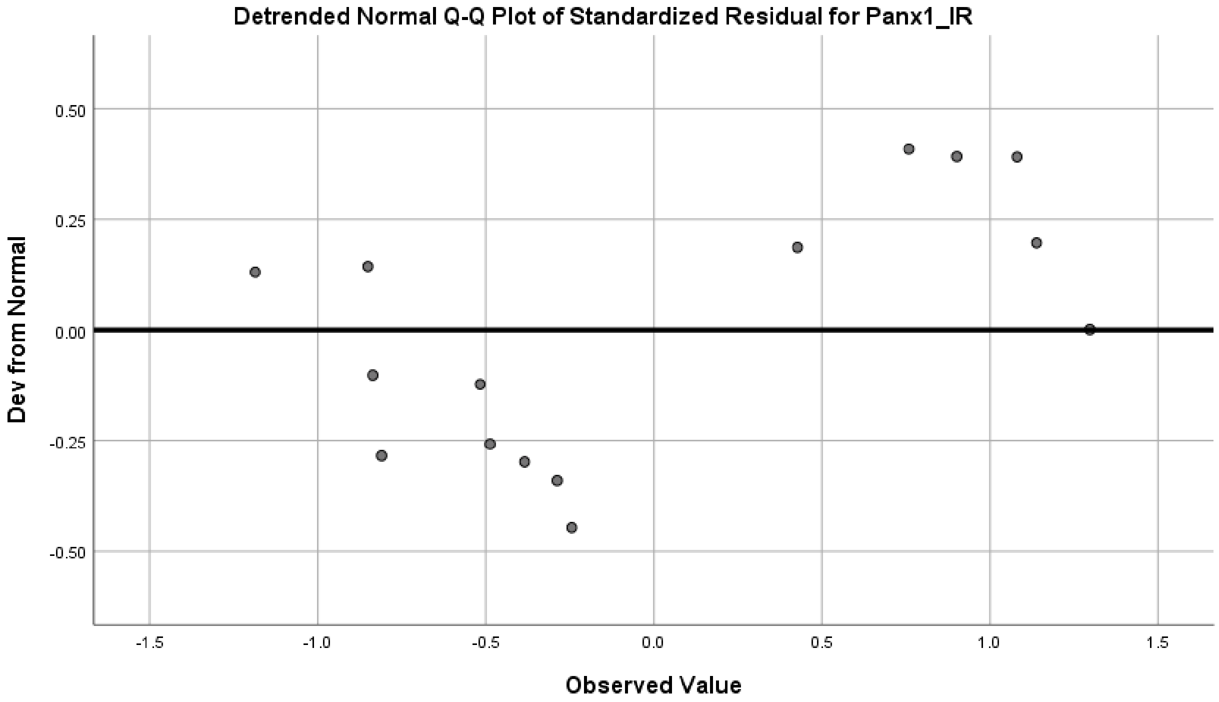

Detrended Normal Q-Q Plot of Standardized Residual for Panx1_IR
Dev from Normal
Observed Value
0.50
0.25
0.00
-0.25
-0.50
-1.5
-1.0
-0.5
0.0
0.5
1.0
1.5

Co-immunoprecipitations Experiments 16 hours

```
NEW FILE.
DATASET NAME DataSet4 WINDOW=FRONT.
DATASET ACTIVATE DataSet4.
DATASET CLOSE DataSet3.
EXAMINE VARIABLES=CRMP2_IP
  /PLOT HISTOGRAM NPPLOT
  /STATISTICS DESCRIPTIVES
  /CINTERVAL 95
  /MISSING LISTWISE
  /NOTOTAL.
```

Explore

Notes

Output Created		13-MAR-2018 19:46:23
Comments		
Input	Active Dataset	DataSet4
	Filter	<none>
	Weight	<none>
	Split File	<none>
	N of Rows in Working Data File	6
Missing Value Handling	Definition of Missing	User-defined missing values for dependent variables are treated as missing.
	Cases Used	Statistics are based on cases with no missing values for any dependent variable or factor used.

Syntax		EXAMINE VARIABLES=CRMP2_IP<br>/PLOT HISTOGRAM NPPLOT<br>/STATISTICS DESCRIPTIVES<br>/CINTERVAL 95<br>/MISSING LISTWISE<br>/NOTOTAL.
Resources	Processor Time	00:00:00.28
	Elapsed Time	00:00:00.28

Case Processing Summary

	Cases					
	Valid		Missing		Total	
	N	Percent	N	Percent	N	Percent
CRMP2_IP	6	100.0%	0	0.0%	6	100.0%

Descriptives

			Statistic	Std. Error
CRMP2_IP	Mean		69.6922	16.15637
	95% Confidence Interval for Mean	Lower Bound	28.1609	
		Upper Bound	111.2235	
	5% Trimmed Mean		71.6631	
	Median		84.0286	
	Variance		1566.170	
	Std. Deviation		39.57486	
	Minimum		2.43	
	Maximum		101.48	
	Range		99.06	
	Interquartile Range		64.93	
	Skewness		-1.129	.845
	Kurtosis		.407	1.741

Tests of Normality

	Kolmogorov-Smirnov[a]			Shapiro-Wilk		
	Statistic	df	Sig.	Statistic	df	Sig.
CRMP2_IP	.268	6	.200*	.844	6	.141

*. This is a lower bound of the true significance.

a. Lilliefors Significance Correction

CRMP2_IP

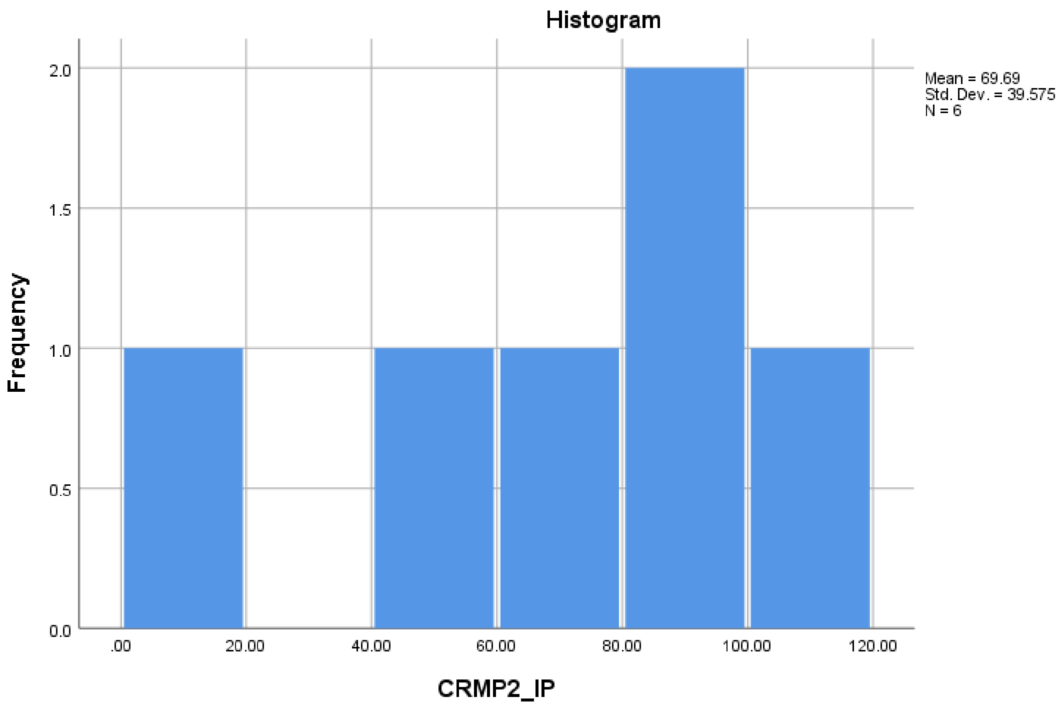

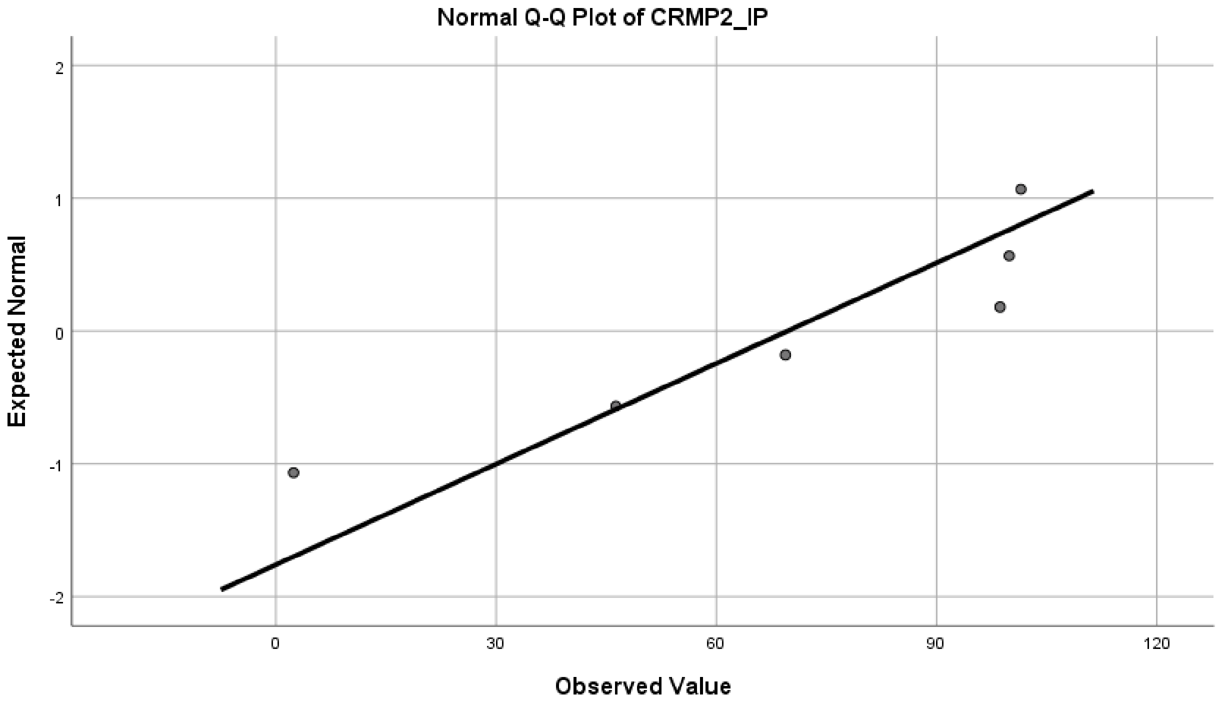

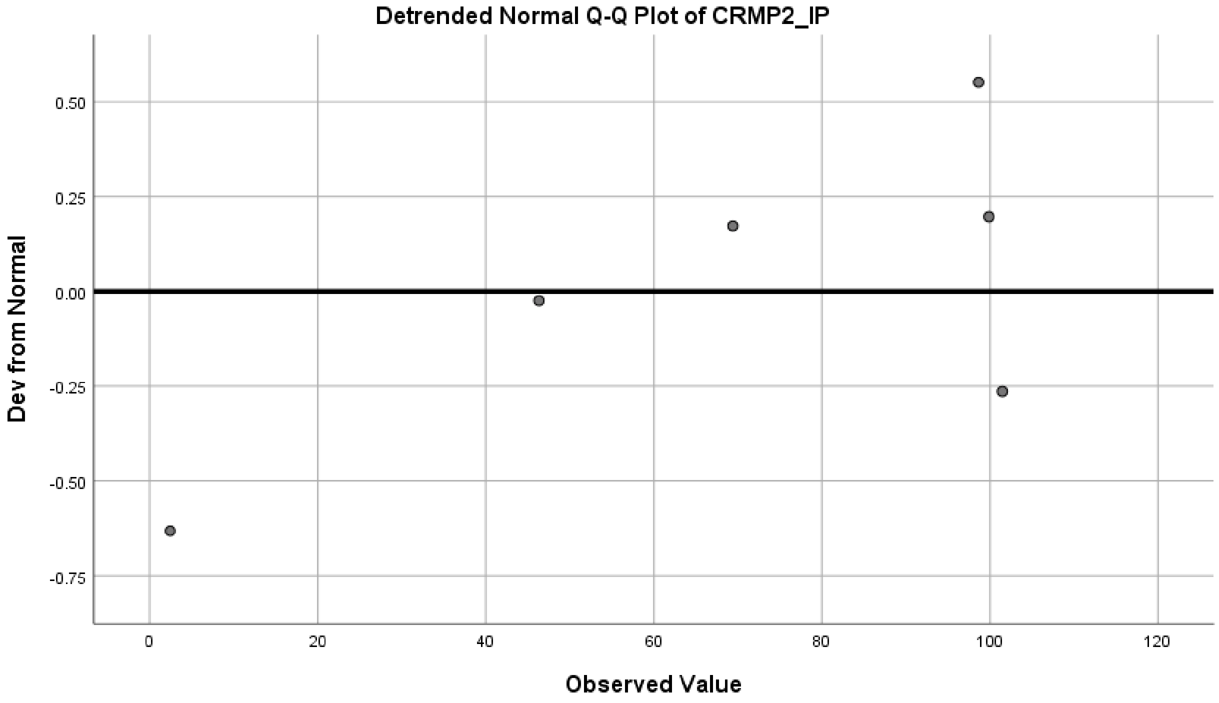


```
T-TEST GROUPS=Treatment(1 2)
  /MISSING=ANALYSIS
```

```
/VARIABLES=CRMP2_IP
/CRITERIA=CI(.95).
```

T-Test

Notes

Output Created		13-MAR-2018 19:46:41
Comments		
Input	Active Dataset	DataSet4
	Filter	<none>
	Weight	<none>
	Split File	<none>
	N of Rows in Working Data File	6
Missing Value Handling	Definition of Missing	User defined missing values are treated as missing.
	Cases Used	Statistics for each analysis are based on the cases with no missing or out-of-range data for any variable in the analysis.
Syntax		T-TEST GROUPS=Treatment(1 2)<br>/MISSING=ANALYSIS<br>/VARIABLES=CRMP2_IP<br>/CRITERIA=CI(.95).
Resources	Processor Time	00:00:00.00
	Elapsed Time	00:00:00.00

Group Statistics

	Treatment	N	Mean	Std. Deviation	Std. Error Mean
CRMP2_IP	Vehicle	3	100.0000	1.42129	.82058
	Probenecid	3	39.3844	34.02515	19.64443

Independent Samples Test

		Levene's Test for Equality of Variances		t-test for Equality of Means	
		F	Sig.	t	df
CRMP2_IP	Equal variances assumed	6.776	.060	3.083	4
	Equal variances not assumed			3.083	2.007

Independent Samples Test

		t-test for Equality of Means			
		Sig. (2-tailed)	Mean Difference	Std. Error Difference	95% Confidence Interval of the Difference Lower
CRMP2_IP	Equal variances assumed	.037	60.61565	19.66156	6.02640
	Equal variances not assumed	.091	60.61565	19.66156	-23.69992

Independent Samples Test

		t-test for Equality of Means 95% Confidence Interval of the Difference Upper
CRMP2_IP	Equal variances assumed	115.20490
	Equal variances not assumed	144.93122

```
UNIANOVA CRMP2_IP BY Treatment
  /METHOD=SSTYPE(3)
  /INTERCEPT=INCLUDE
  /SAVE=ZRESID
  /PRINT DESCRIPTIVE HOMOGENEITY
  /CRITERIA=ALPHA(.05)
  /DESIGN=Treatment.
```

Univariate Analysis of Variance

Notes

Output Created		13-MAR-2018 19:47:21
Comments		
Input	Active Dataset	DataSet4
	Filter	<none>
	Weight	<none>
	Split File	<none>
	N of Rows in Working Data File	6
Missing Value Handling	Definition of Missing	User-defined missing values are treated as missing.
	Cases Used	Statistics are based on all cases with valid data for all variables in the model.
Syntax		UNIANOVA CRMP2_IP BY Treatment<br>/METHOD=SSTYPE(3)<br>/INTERCEPT=INCLUDE<br>/SAVE=ZRESID<br>/PRINT DESCRIPTIVE HOMOGENEITY<br>/CRITERIA=ALPHA(.05)<br>/DESIGN=Treatment.
Resources	Processor Time	00:00:00.02
	Elapsed Time	00:00:00.01
Variables Created or Modified	ZRE_1	Standardized Residual for CRMP2_IP

Between-Subjects Factors

		Value Label	N
Treatment	1.00	Vehicle	3
	2.00	Probenecid	3

Descriptive Statistics

Dependent Variable: CRMP2_IP

Treatment	Mean	Std. Deviation	N
Vehicle	100.0000	1.42129	3
Probenecid	39.3844	34.02515	3
Total	69.6922	39.57486	6

Levene's Test of Equality of Error Variances[a,b]

		Levene Statistic	df1	df2	Sig.
CRMP2_IP	Based on Mean	6.776	1	4	.060
	Based on Median	2.841	1	4	.167
	Based on Median and with adjusted df	2.841	1	2.006	.234
	Based on trimmed mean	6.451	1	4	.064

Tests the null hypothesis that the error variance of the dependent variable is equal across groups.[a,b]

a. Dependent variable: CRMP2_IP

b. Design: Intercept + Treatment

Tests of Between-Subjects Effects

Dependent Variable: CRMP2_IP

Source	Type III Sum of Squares	df	Mean Square	F	Sig.
Corrected Model	5511.386[a]	1	5511.386	9.505	.037
Intercept	29142.007	1	29142.007	50.256	.002
Treatment	5511.386	1	5511.386	9.505	.037
Error	2319.462	4	579.866		
Total	36972.854	6			
Corrected Total	7830.848	5			

a. R Squared = .704 (Adjusted R Squared = .630)

```
EXAMINE VARIABLES=ZRE_1
  /PLOT HISTOGRAM NPPLOT
  /STATISTICS DESCRIPTIVES
  /CINTERVAL 95
  /MISSING LISTWISE
  /NOTOTAL.
```

Explore

Notes

Output Created		13-MAR-2018 19:47:32
Comments		
Input	Active Dataset	DataSet4
	Filter	<none>
	Weight	<none>
	Split File	<none>
	N of Rows in Working Data File	6
Missing Value Handling	Definition of Missing	User-defined missing values for dependent variables are treated as missing.
	Cases Used	Statistics are based on cases with no missing values for any dependent variable or factor used.
Syntax		EXAMINE VARIABLES=ZRE_1 /PLOT HISTOGRAM NPPLOT /STATISTICS DESCRIPTIVES /CINTERVAL 95 /MISSING LISTWISE /NOTOTAL.
Resources	Processor Time	00:00:00.30
	Elapsed Time	00:00:00.27

Case Processing Summary

Cases

	Valid		Missing		Total	
	N	Percent	N	Percent	N	Percent
Standardized Residual for CRMP2_IP	6	100.0%	0	0.0%	6	100.0%

Descriptives

			Statistic	Std. Error
Standardized Residual for CRMP2_IP	Mean		.0000	.36515
	95% Confidence Interval for Mean	Lower Bound	-.9386	
		Upper Bound	.9386	
	5% Trimmed Mean		.0160	
	Median		.0280	
	Variance		.800	
	Std. Deviation		.89443	
	Minimum		-1.53	
	Maximum		1.25	
	Range		2.78	
	Interquartile Range		.95	
	Skewness		-.693	.845
	Kurtosis		2.470	1.741

Tests of Normality

	Kolmogorov-Smirnov[a]			Shapiro-Wilk		
	Statistic	df	Sig.	Statistic	df	Sig.
Standardized Residual for CRMP2_IP	.308	6	.077	.894	6	.339

a. Lilliefors Significance Correction

Standardized Residual for CRMP2_IP

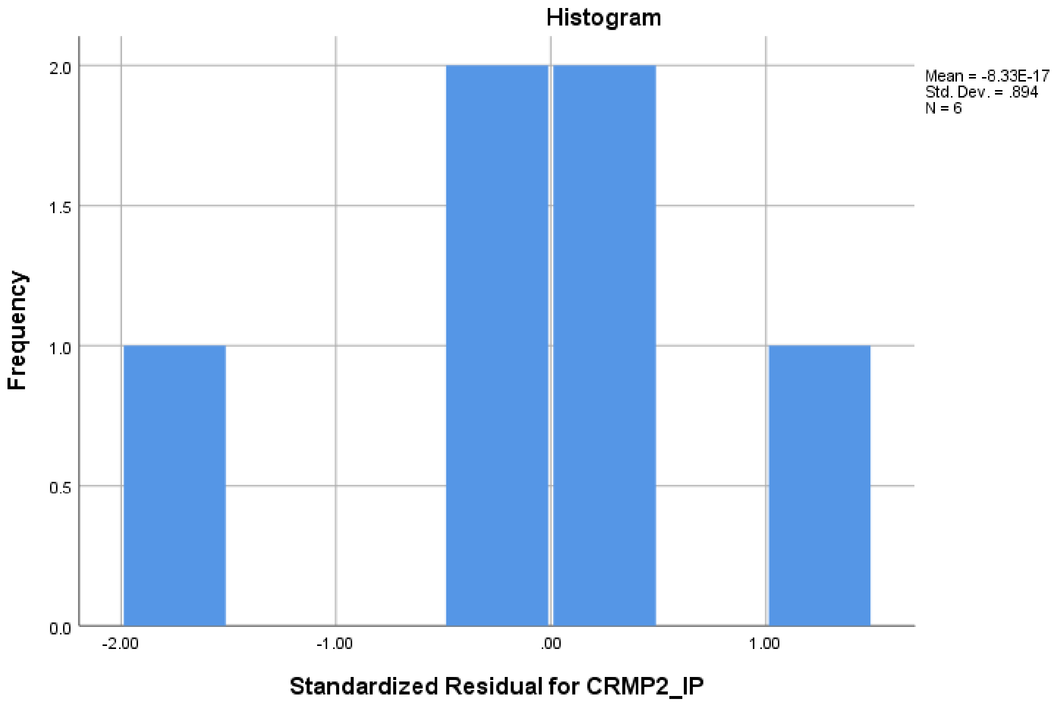
Histogram
Mean = -8.33E-17
Std. Dev. = .894
N = 6
Frequency
2.0
1.5
1.0
0.5
0.0
-2.00
-1.00
.00
1.00
Standardized Residual for CRMP2_IP

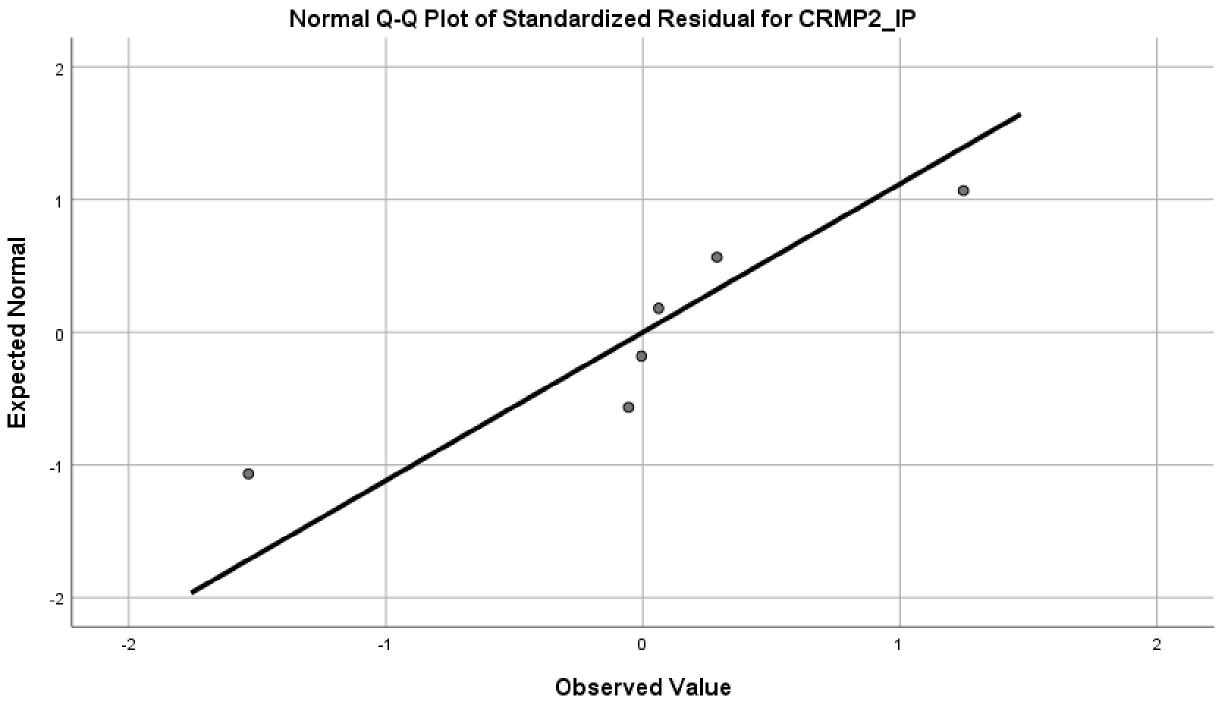
Normal Q-Q Plot of Standardized Residual for CRMP2_IP
Expected Normal
2
1
0
-1
-2
Observed Value

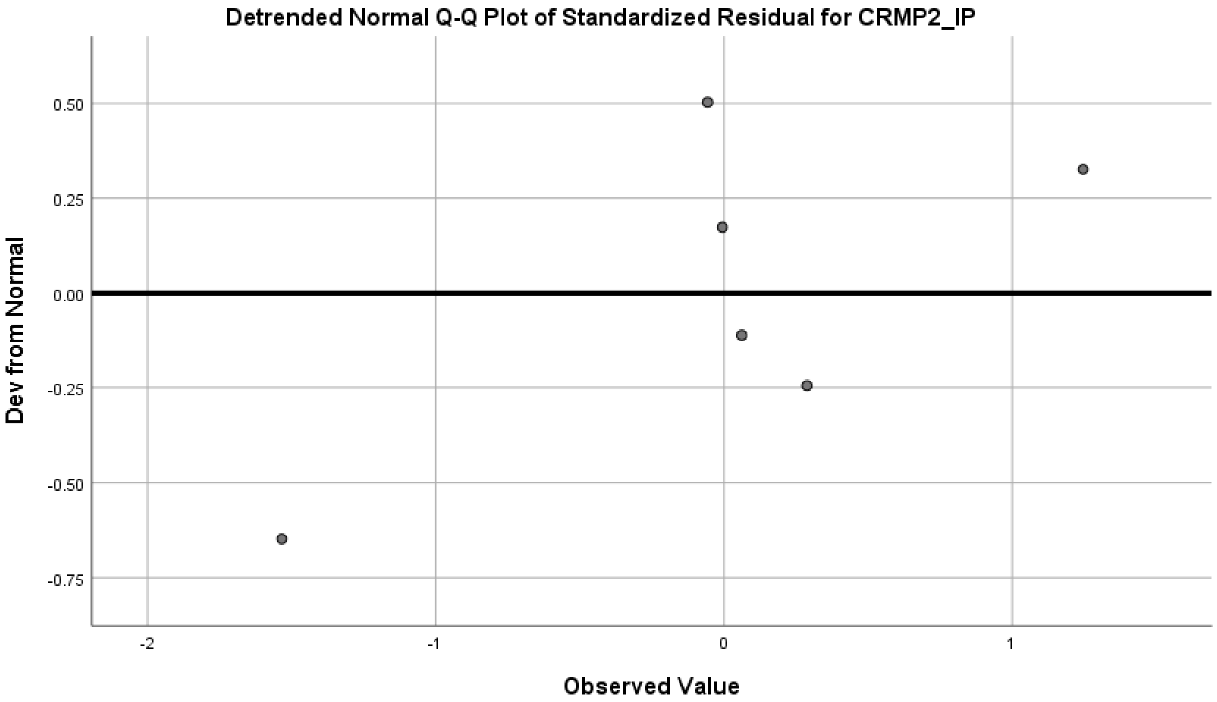
Detrended Normal Q-Q Plot of Standardized Residual for CRMP2_IP
Dev from Normal
0.50
0.25
0.00
-0.25
-0.50
-0.75
-2
-1
0
1
Observed Value

Co-immunoprecipitations Experiments – 30 min

```
NEW FILE.
DATASET NAME DataSet5 WINDOW=FRONT.
DATASET ACTIVATE DataSet5.
DATASET CLOSE DataSet4.
EXAMINE VARIABLES=CRMP2_IP
  /PLOT HISTOGRAM NPPLOT
  /STATISTICS DESCRIPTIVES
  /CINTERVAL 95
  /MISSING LISTWISE
  /NOTOTAL.
```

Explore

Notes

Output Created		13-MAR-2018 19:53:34
Comments		
Input	Active Dataset	DataSet5
	Filter	<none>
	Weight	<none>
	Split File	<none>
	N of Rows in Working Data File	6
Missing Value Handling	Definition of Missing	User-defined missing values for dependent variables are treated as missing.
	Cases Used	Statistics are based on cases with no missing values for any dependent variable or factor used.

Syntax		EXAMINE VARIABLES=CRMP2_IP<br>/PLOT HISTOGRAM NPPLOT<br>/STATISTICS DESCRIPTIVES<br>/CINTERVAL 95<br>/MISSING LISTWISE<br>/NOTOTAL.
Resources	Processor Time	00:00:00.33
	Elapsed Time	00:00:00.28

Case Processing Summary

	Cases					
	Valid		Missing		Total	
	N	Percent	N	Percent	N	Percent
CRMP2_IP	6	100.0%	0	0.0%	6	100.0%

Descriptives

			Statistic	Std. Error
CRMP2_IP	Mean		83.8639	9.55569
	95% Confidence Interval for Mean	Lower Bound	59.3002	
		Upper Bound	108.4276	
	5% Trimmed Mean		83.9662	
	Median		78.0122	
	Variance		547.868	
	Std. Deviation		23.40658	
	Minimum		50.89	
	Maximum		115.00	
	Range		64.11	
	Interquartile Range		40.27	
	Skewness		.121	.845
	Kurtosis		-.634	1.741

Tests of Normality

	Kolmogorov-Smirnov[a]			Shapiro-Wilk		
	Statistic	df	Sig.	Statistic	df	Sig.
CRMP2_IP	.262	6	.200*	.925	6	.539

*. This is a lower bound of the true significance.

a. Lilliefors Significance Correction

CRMP2_IP

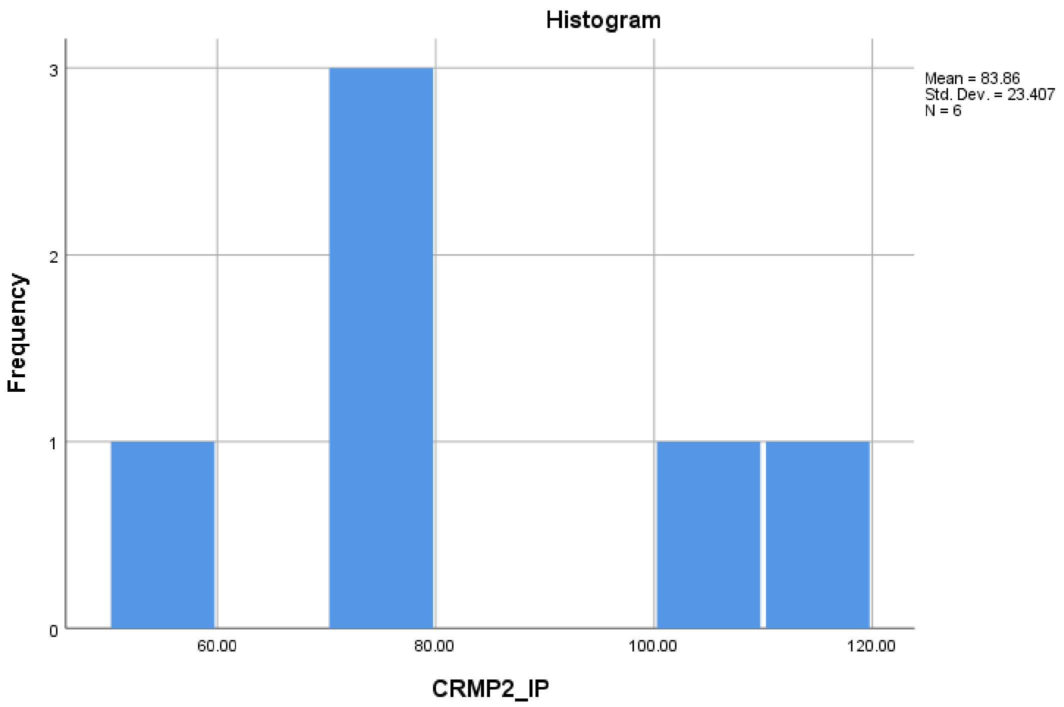

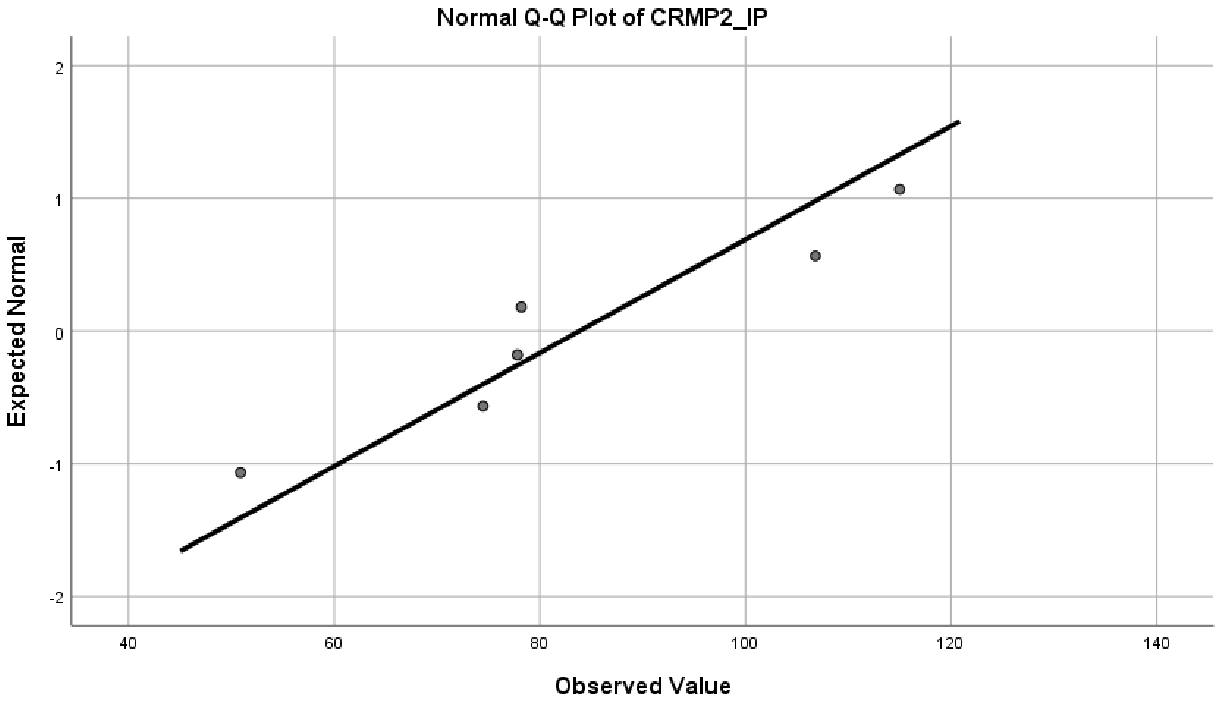

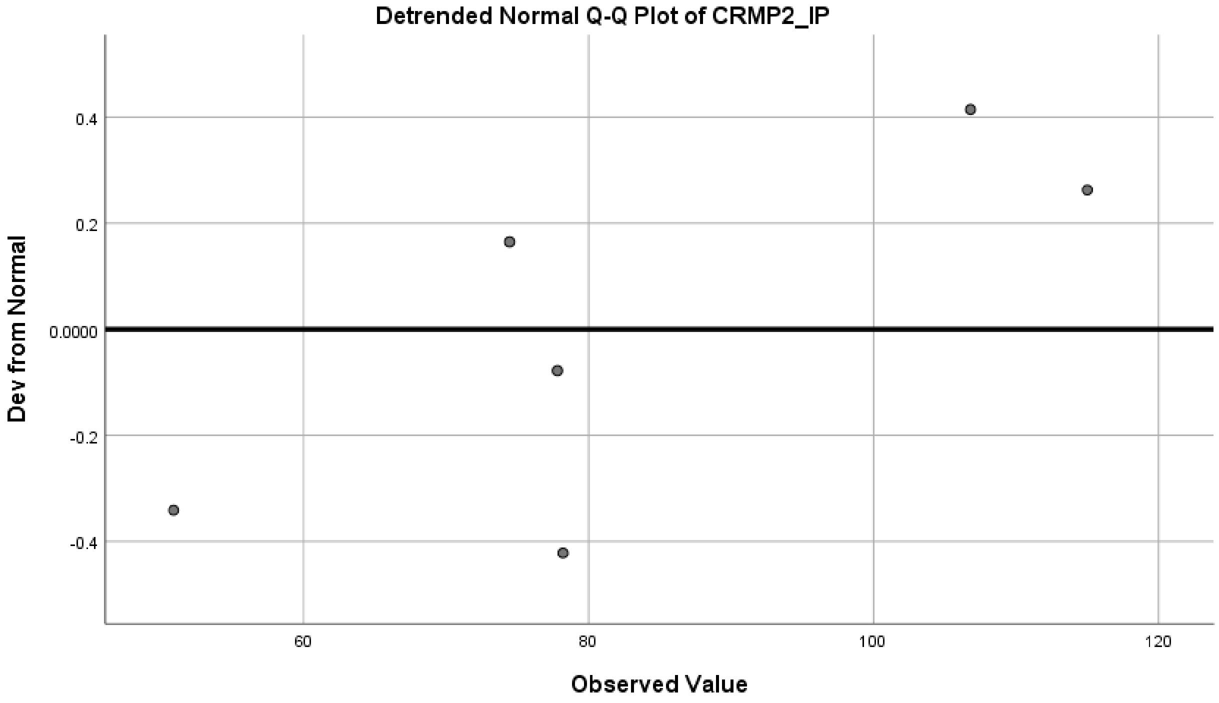


```
T-TEST GROUPS=Treatment(1 2)
  /MISSING=ANALYSIS
```

```
/VARIABLES=CRMP2_IP
/CRITERIA=CI(.95).
```

T-Test

Notes

Output Created		13-MAR-2018 19:53:52
Comments		
Input	Active Dataset	DataSet5
	Filter	<none>
	Weight	<none>
	Split File	<none>
	N of Rows in Working Data File	6
Missing Value Handling	Definition of Missing	User defined missing values are treated as missing.
	Cases Used	Statistics for each analysis are based on the cases with no missing or out-of-range data for any variable in the analysis.
Syntax		T-TEST GROUPS=Treatment(1 2) /MISSING=ANALYSIS /VARIABLES=CRMP2_IP /CRITERIA=CI(.95).
Resources	Processor Time	00:00:00.00
	Elapsed Time	00:00:00.00

Group Statistics

	Treatment	N	Mean	Std. Deviation	Std. Error Mean
CRMP2_IP	Vehicle	3	100.0000	19.31676	11.15254
	Probenecid	3	67.7278	14.67697	8.47375

Independent Samples Test

		Levene's Test for Equality of Variances		t-test for Equality of Means	
		F	Sig.	t	df
CRMP2_IP	Equal variances assumed	.396	.563	2.304	4
	Equal variances not assumed			2.304	3.732

Independent Samples Test

		t-test for Equality of Means			
					95% Confidence Interval of the Difference
		Sig. (2-tailed)	Mean Difference	Std. Error Difference	Lower
CRMP2_IP	Equal variances assumed	.083	32.27216	14.00655	-6.61627
	Equal variances not assumed	.087	32.27216	14.00655	-7.74286

Independent Samples Test

		t-test for Equality of Means
		95% Confidence Interval of the Difference
		Upper
CRMP2_IP	Equal variances assumed	71.16058
	Equal variances not assumed	72.28718

```
UNIANOVA CRMP2_IP BY Treatment
  /METHOD=SSTYPE(3)
  /INTERCEPT=INCLUDE
  /SAVE=ZRESID
  /PRINT DESCRIPTIVE HOMOGENEITY
  /CRITERIA=ALPHA(.05)
  /DESIGN=Treatment.
```

Univariate Analysis of Variance

Notes

Output Created		13-MAR-2018 19:54:18
Comments		
Input	Active Dataset	DataSet5
	Filter	<none>
	Weight	<none>
	Split File	<none>
	N of Rows in Working Data File	6
Missing Value Handling	Definition of Missing	User-defined missing values are treated as missing.
	Cases Used	Statistics are based on all cases with valid data for all variables in the model.
Syntax		UNIANOVA CRMP2_IP BY Treatment<br>/METHOD=SSTYPE(3)<br>/INTERCEPT=INCLUDE<br>/SAVE=ZRESID<br>/PRINT DESCRIPTIVE HOMOGENEITY<br>/CRITERIA=ALPHA(.05)<br>/DESIGN=Treatment.
Resources	Processor Time	00:00:00.02
	Elapsed Time	00:00:00.01
Variables Created or Modified	ZRE_1	Standardized Residual for CRMP2_IP

Between-Subjects Factors

		Value Label	N
Treatment	1.00	Vehicle	3
	2.00	Probenecid	3

Descriptive Statistics

Dependent Variable: CRMP2_IP

Treatment	Mean	Std. Deviation	N
Vehicle	100.0000	19.31676	3
Probenecid	67.7278	14.67697	3
Total	83.8639	23.40658	6

Levene's Test of Equality of Error Variances[a,b]

		Levene Statistic	df1	df2	Sig.
CRMP2_IP	Based on Mean	.396	1	4	.563
	Based on Median	.085	1	4	.785
	Based on Median and with adjusted df	.085	1	3.920	.785
	Based on trimmed mean	.353	1	4	.584

Tests the null hypothesis that the error variance of the dependent variable is equal across groups.[a,b]

a. Dependent variable: CRMP2_IP

b. Design: Intercept + Treatment

Tests of Between-Subjects Effects

Dependent Variable: CRMP2_IP

Source	Type III Sum of Squares	df	Mean Square	F	Sig.
Corrected Model	1562.238[a]	1	1562.238	5.309	.083
Intercept	42198.947	1	42198.947	143.400	.000
Treatment	1562.238	1	1562.238	5.309	.083
Error	1177.101	4	294.275		
Total	44938.287	6			
Corrected Total	2739.339	5			

a. R Squared = .570 (Adjusted R Squared = .463)

```
EXAMINE VARIABLES=ZRE_1
  /PLOT HISTOGRAM NPPLOT
  /STATISTICS DESCRIPTIVES
  /CINTERVAL 95
  /MISSING LISTWISE
  /NOTOTAL.
```

Explore

Notes

Output Created		13-MAR-2018 19:54:31
Comments		
Input	Active Dataset	DataSet5
	Filter	<none>
	Weight	<none>
	Split File	<none>
	N of Rows in Working Data File	6
Missing Value Handling	Definition of Missing	User-defined missing values for dependent variables are treated as missing.
	Cases Used	Statistics are based on cases with no missing values for any dependent variable or factor used.
Syntax		EXAMINE VARIABLES=ZRE_1<br>/PLOT HISTOGRAM NPPLOT<br>/STATISTICS DESCRIPTIVES<br>/CINTERVAL 95<br>/MISSING LISTWISE<br>/NOTOTAL.
Resources	Processor Time	00:00:00.27
	Elapsed Time	00:00:00.28

Case Processing Summary

Cases

	Valid		Missing		Total	
	N	Percent	N	Percent	N	Percent
Standardized Residual for CRMP2_IP	6	100.0%	0	0.0%	6	100.0%

Descriptives

			Statistic	Std. Error
Standardized Residual for CRMP2_IP	Mean		.0000	.36515
	95% Confidence Interval for Mean	Lower Bound	-.9386	
		Upper Bound	.9386	
	5% Trimmed Mean		.0220	
	Median		.3948	
	Variance		.800	
	Std. Deviation		.89443	
	Minimum		-1.27	
	Maximum		.87	
	Range		2.14	
	Interquartile Range		1.71	
	Skewness		-.839	.845
	Kurtosis		-1.561	1.741

Tests of Normality

	Kolmogorov-Smirnov[a]			Shapiro-Wilk		
	Statistic	df	Sig.	Statistic	df	Sig.
Standardized Residual for CRMP2_IP	.336	6	.032	.828	6	.104

a. Lilliefors Significance Correction

Standardized Residual for CRMP2_IP

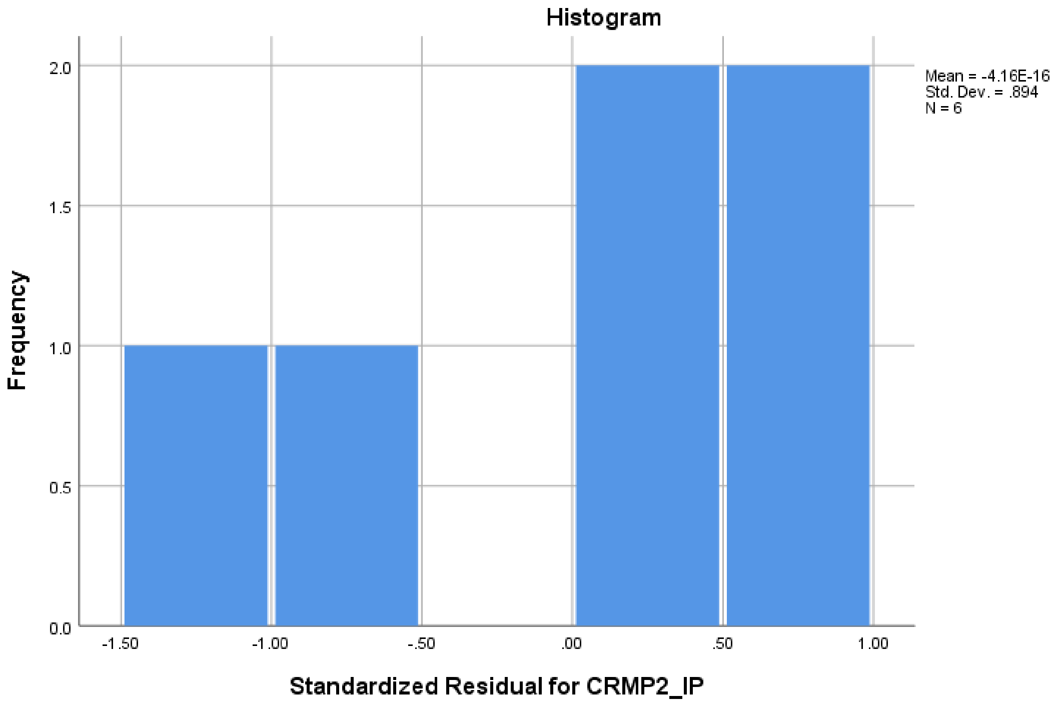

Histogram
Mean = -4.16E-16
Std. Dev. = .894
N = 6
Frequency
0.0
0.5
1.0
1.5
2.0
-1.50
-1.00
-.50
.00
.50
1.00
Standardized Residual for CRMP2_IP

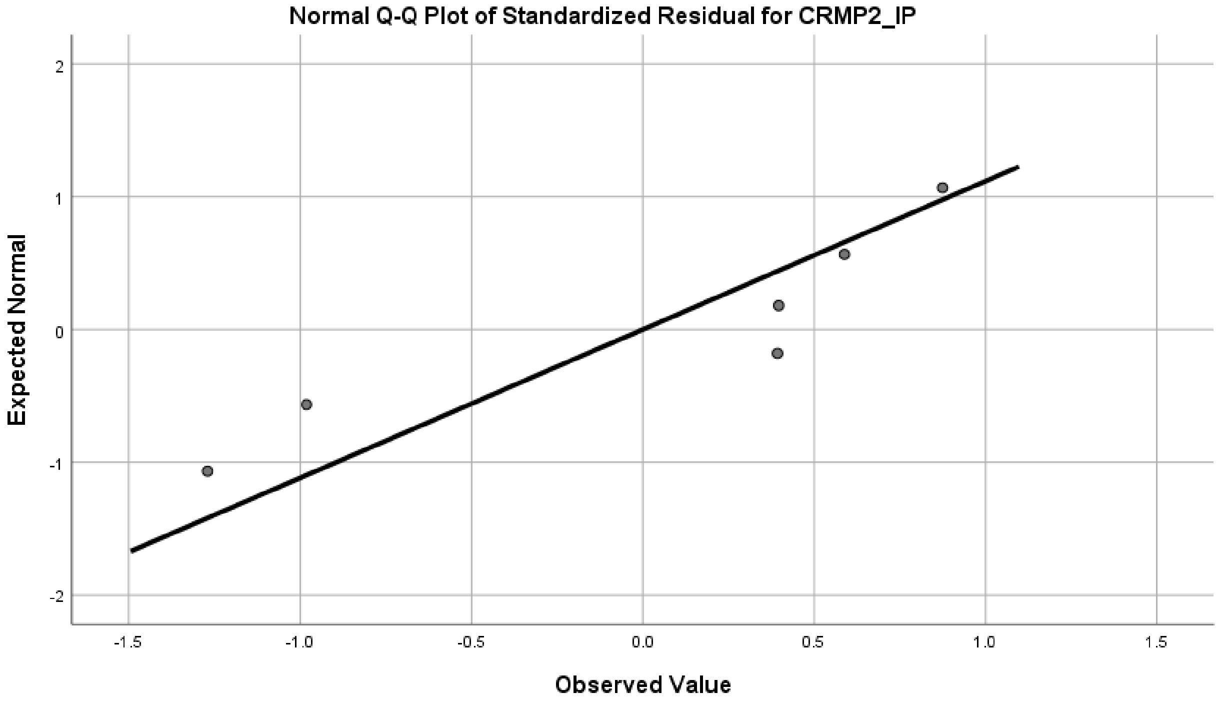

Normal Q-Q Plot of Standardized Residual for CRMP2_IP
Expected Normal
2
1
0
-1
-2
-1.5
-1.0
-0.5
0.0
0.5
1.0
1.5
Observed Value

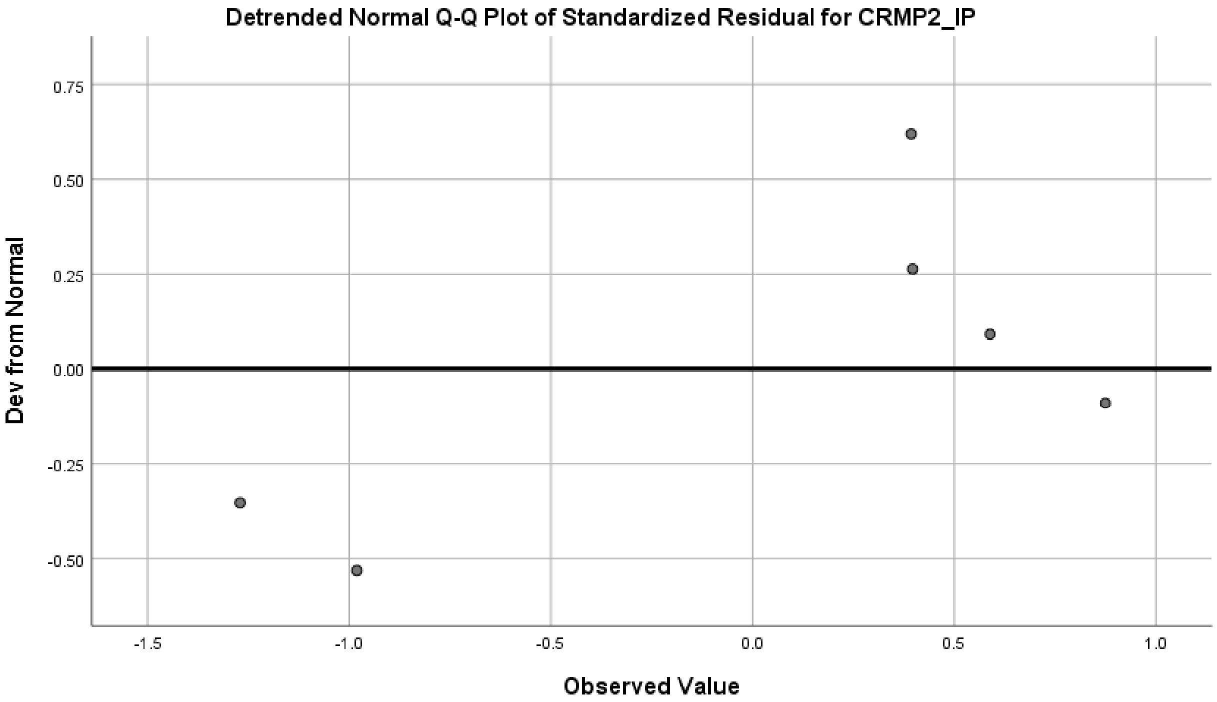
Detrended Normal Q-Q Plot of Standardized Residual for CRMP2_IP
Dev from Normal
Observed Value
0.75
0.50
0.25
0.00
-0.25
-0.50
-1.5
-1.0
-0.5
0.0
0.5
1.0

Western Blots – Panx1 & CRMP2 % control Immunoreactivity (IR) from Inputs normalized to GAPDH IR

```
NEW FILE.
DATASET NAME DataSet3 WINDOW=FRONT.
DATASET ACTIVATE DataSet3.
DATASET CLOSE DataSet2.
EXAMINE VARIABLES=CRMP2_IR Panx1_IR
  /PLOT HISTOGRAM NPPLOT
  /STATISTICS DESCRIPTIVES
  /CINTERVAL 95
  /MISSING LISTWISE
  /NOTOTAL.
```

Explore

Notes

Output Created		13-MAR-2018 18:24:18
Comments		
Input	Active Dataset	DataSet3
	Filter	<none>
	Weight	<none>
	Split File	<none>
	N of Rows in Working Data File	6
Missing Value Handling	Definition of Missing	User-defined missing values for dependent variables are treated as missing.
	Cases Used	Statistics are based on cases with no missing values for any dependent variable or factor used.

Syntax		EXAMINE VARIABLES=CRMP2_IR Panx1_IR<br>/PLOT HISTOGRAM NPPLOT<br>/STATISTICS DESCRIPTIVES<br>/CINTERVAL 95<br>/MISSING LISTWISE<br>/NOTOTAL.
Resources	Processor Time	00:00:00.64
	Elapsed Time	00:00:00.58

Case Processing Summary

	Cases					
	Valid		Missing		Total	
	N	Percent	N	Percent	N	Percent
CRMP2_IR	6	100.0%	0	0.0%	6	100.0%
Panx1_IR	6	100.0%	0	0.0%	6	100.0%

Descriptives

			Statistic	Std. Error
CRMP2_IR	Mean		85.3197	9.08993
	95% Confidence Interval for Mean	Lower Bound	61.9533	
		Upper Bound	108.6861	
	5% Trimmed Mean		84.6946	
	Median		87.5208	
	Variance		495.761	
	Std. Deviation		22.26569	
	Minimum		60.89	
	Maximum		121.00	
	Range		60.10	
	Interquartile Range		38.10	
	Skewness		.511	.845
	Kurtosis		.214	1.741
Panx1_IR	Mean		90.9172	11.89889

	95% Confidence Interval for Mean	Lower Bound	60.3301	
		Upper Bound	121.5042	
	5% Trimmed Mean		91.2349	
	Median		89.7075	
	Variance		849.502	
	Std. Deviation		29.14622	
	Minimum		44.12	
	Maximum		132.00	
	Range		87.88	
	Interquartile Range		40.07	
	Skewness		-.359	.845
	Kurtosis		1.236	1.741

Tests of Normality

	Kolmogorov-Smirnov[a]			Shapiro-Wilk		
	Statistic	df	Sig.	Statistic	df	Sig.
CRMP2_IR	.200	6	.200*	.911	6	.445
Panx1_IR	.225	6	.200*	.967	6	.873

*. This is a lower bound of the true significance.

a. Lilliefors Significance Correction

CRMP2_IR

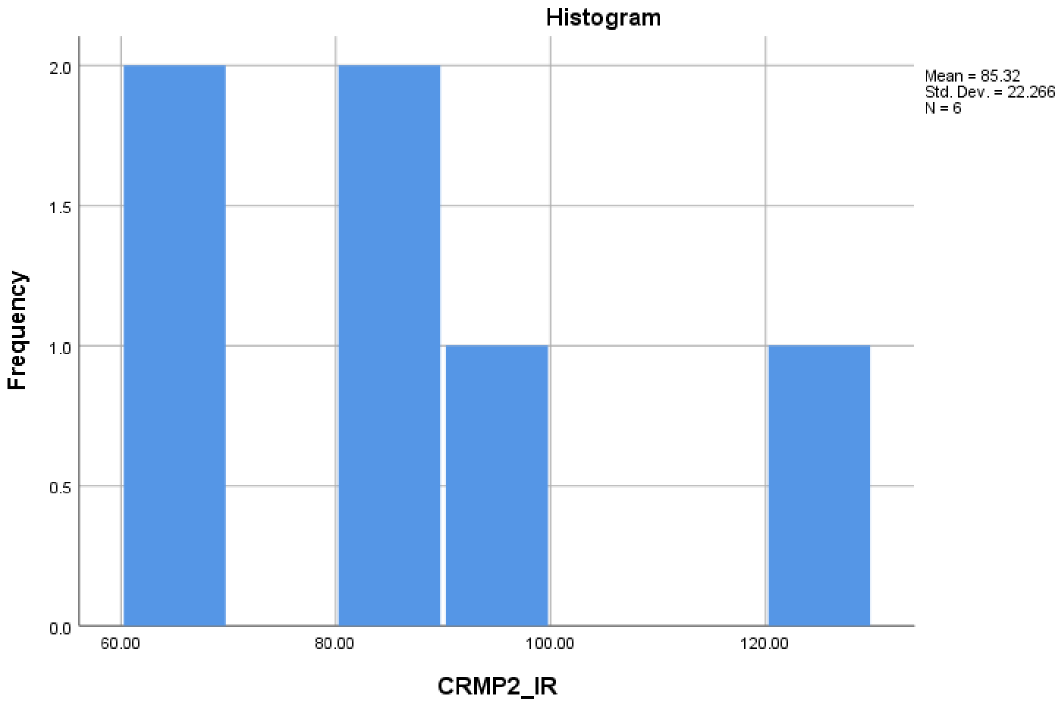
Histogram
Mean = 85.32
Std. Dev. = 22.266
N = 6
Frequency
2.0
1.5
1.0
0.5
0.0
60.00
80.00
100.00
120.00
CRMP2_IR

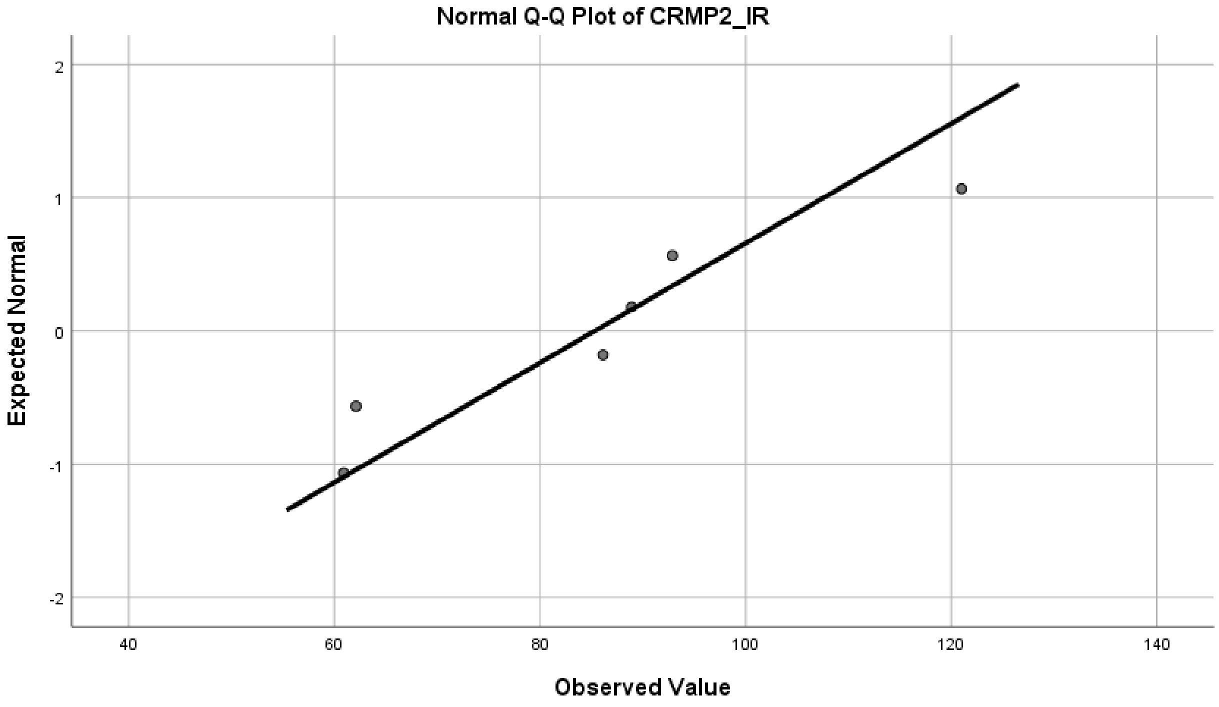
Normal Q-Q Plot of CRMP2_IR
Expected Normal
2
1
0
-1
-2
40
60
80
100
120
140
Observed Value

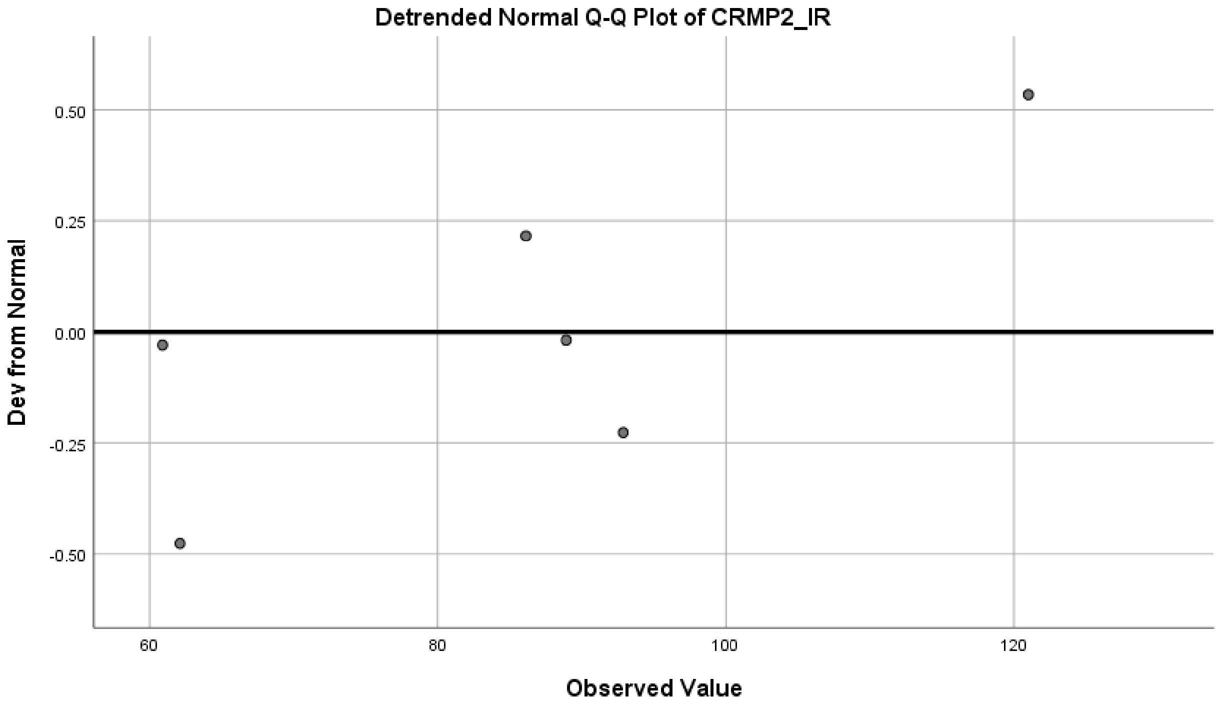
Detrended Normal Q-Q Plot of CRMP2_IR
Dev from Normal
0.50
0.25
0.00
-0.25
-0.50
60
80
100
120
Observed Value

Panx1_IR

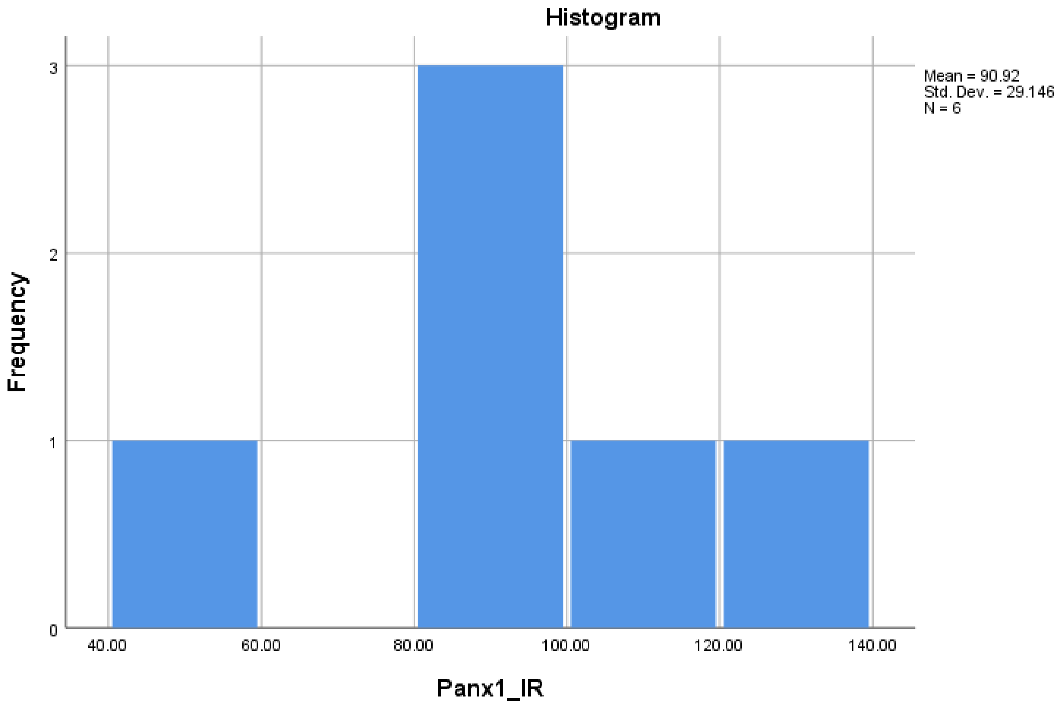
Histogram
Mean = 90.92
Std. Dev. = 29.146
N = 6
Frequency
0
1
2
3
40.00
60.00
80.00
100.00
120.00
140.00
Panx1_IR

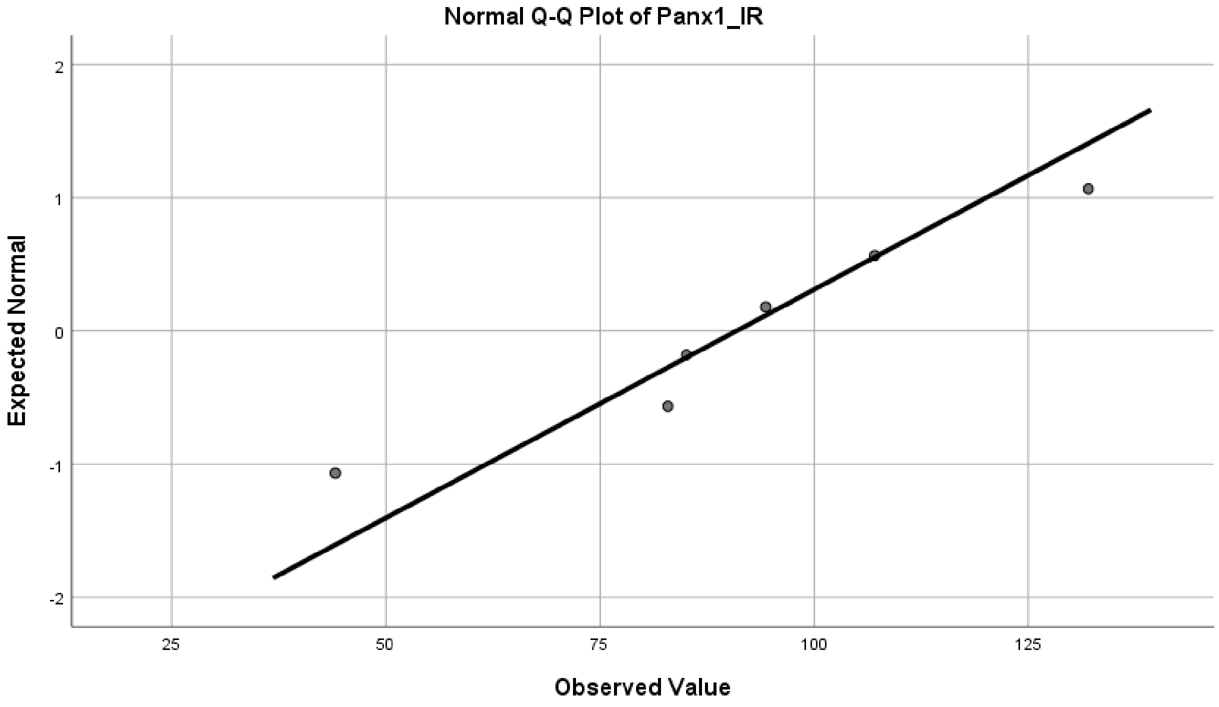
Normal Q-Q Plot of Panx1_IR
Expected Normal
2
1
0
-1
-2
25
50
75
100
125
Observed Value

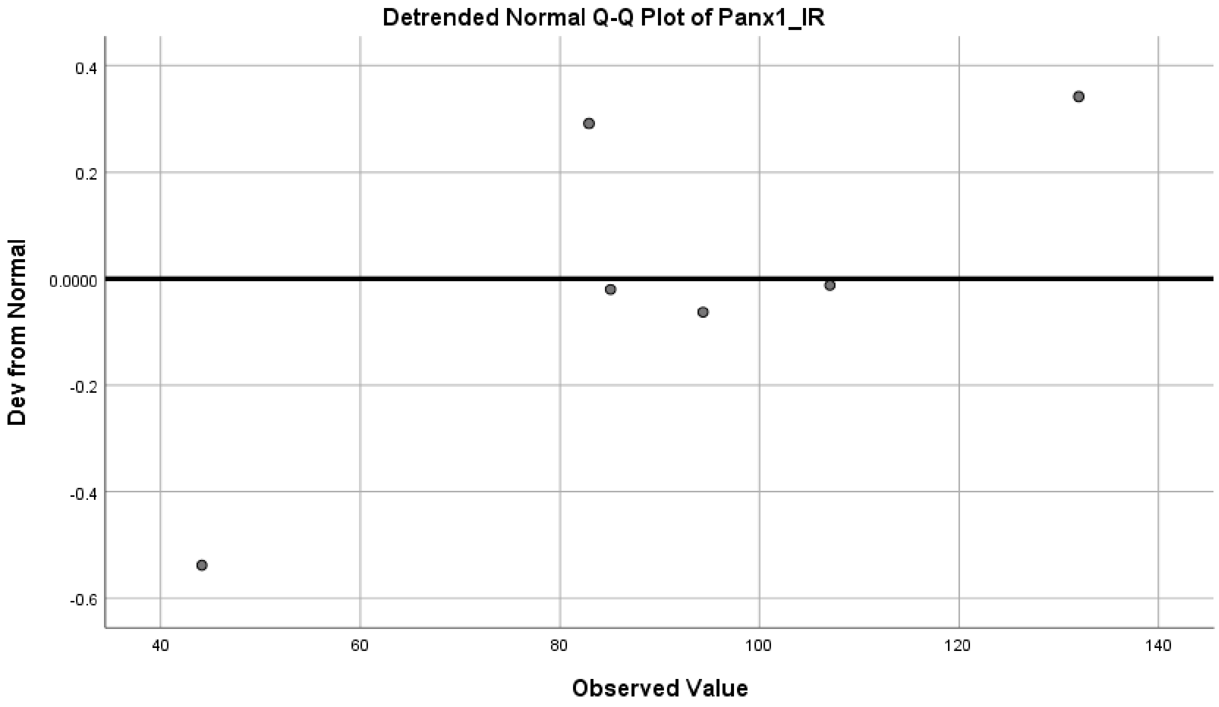


```
T-TEST GROUPS=Treatment(1 2)
  /MISSING=ANALYSIS
  /VARIABLES=CRMP2_IR
  /CRITERIA=CI(.95).
```

T-Test

Notes

Output Created		13-MAR-2018 18:24:47
Comments		
Input	Active Dataset	DataSet3
	Filter	<none>
	Weight	<none>
	Split File	<none>
	N of Rows in Working Data File	6

Missing Value Handling	Definition of Missing	User defined missing values are treated as missing.
	Cases Used	Statistics for each analysis are based on the cases with no missing or out-of-range data for any variable in the analysis.
Syntax		T-TEST GROUPS=Treatment(1 2) /MISSING=ANALYSIS /VARIABLES=CRMP2_IR /CRITERIA=CI(.95).
Resources	Processor Time	00:00:00.00
	Elapsed Time	00:00:00.01

Group Statistics

	Treatment	N	Mean	Std. Deviation	Std. Error Mean
CRMP2_IR	Vehicle	3	100.0000	18.49343	10.67719
	Probenecid	3	70.6394	15.83856	9.14440

Independent Samples Test

		Levene's Test for Equality of Variances		t-test for Equality of Means	
		F	Sig.	t	df
CRMP2_IR	Equal variances assumed	.129	.737	2.089	4
	Equal variances not assumed			2.089	3.908

Independent Samples Test

		t-test for Equality of Means		
		Sig. (2-tailed)	Mean Difference	Std. Error Difference
CRMP2_IR	Equal variances assumed	.105	29.36064	14.05782
	Equal variances not assumed	.107	29.36064	14.05782

Independent Samples Test

		t-test for Equality of Means	
		95% Confidence Interval of the Difference	
		Lower	Upper
CRMP2_IR	Equal variances assumed	-9.67013	68.39141
	Equal variances not assumed	-10.03666	68.75794

```
T-TEST GROUPS=Treatment(1 2)
  /MISSING=ANALYSIS
  /VARIABLES=Panx1_IR
  /CRITERIA=CI(.95).
```

T-Test

Notes

Output Created		13-MAR-2018 18:24:57
Comments		
Input	Active Dataset	DataSet3
	Filter	<none>
	Weight	<none>
	Split File	<none>
	N of Rows in Working Data File	6
Missing Value Handling	Definition of Missing	User defined missing values are treated as missing.
	Cases Used	Statistics for each analysis are based on the cases with no missing or out-of-range data for any variable in the analysis.
Syntax		T-TEST GROUPS=Treatment(1 2) /MISSING=ANALYSIS /VARIABLES=Panx1_IR /CRITERIA=CI(.95).
Resources	Processor Time	00:00:00.02

Elapsed Time	00:00:00.00

Group Statistics

	Treatment	N	Mean	Std. Deviation	Std. Error Mean
Panx1_IR	Vehicle	3	100.0000	27.73057	16.01025
	Probenecid	3	81.8343	33.27576	19.21177

Independent Samples Test

		Levene's Test for Equality of Variances		t-test for Equality of Means	
		F	Sig.	t	df
Panx1_IR	Equal variances assumed	.178	.695	.726	4
	Equal variances not assumed			.726	3.874

Independent Samples Test

		t-test for Equality of Means			
		Sig. (2-tailed)	Mean Difference	Std. Error Difference	95% Confidence Interval of the Difference Lower
Panx1_IR	Equal variances assumed	.508	18.16570	25.00840	-51.26876
	Equal variances not assumed	.509	18.16570	25.00840	-52.16808

Independent Samples Test

		t-test for Equality of Means 95% Confidence Interval of the Difference Upper
Panx1_IR	Equal variances assumed	87.60015
	Equal variances not assumed	88.49947

```
UNIANOVA CRMP2_IR BY Treatment
  /METHOD=SSTYPE(3)
  /INTERCEPT=INCLUDE
  /SAVE=ZRESID
  /PRINT DESCRIPTIVE HOMOGENEITY
```

```
/CRITERIA=ALPHA(.05)
/DESIGN=Treatment.
```

Univariate Analysis of Variance

Notes

Output Created		13-MAR-2018 18:25:42
Comments		
Input	Active Dataset	DataSet3
	Filter	<none>
	Weight	<none>
	Split File	<none>
	N of Rows in Working Data File	6
Missing Value Handling	Definition of Missing	User-defined missing values are treated as missing.
	Cases Used	Statistics are based on all cases with valid data for all variables in the model.
Syntax		UNIANOVA CRMP2_IR BY Treatment<br>/METHOD=SSTYPE(3)<br>/INTERCEPT=INCLUDE<br>/SAVE=ZRESID<br>/PRINT DESCRIPTIVE HOMOGENEITY<br>/CRITERIA=ALPHA(.05)<br>/DESIGN=Treatment.
Resources	Processor Time	00:00:00.00
	Elapsed Time	00:00:00.01
Variables Created or Modified	ZRE_1	Standardized Residual for CRMP2_IR

Between-Subjects Factors

		Value Label	N
Treatment	1.00	Vehicle	3
	2.00	Probenecid	3

Descriptive Statistics

Dependent Variable: CRMP2_IR

Treatment	Mean	Std. Deviation	N
Vehicle	100.0000	18.49343	3
Probenecid	70.6394	15.83856	3
Total	85.3197	22.26569	6

Levene's Test of Equality of Error Variances[a,b]

		Levene Statistic	df1	df2	Sig.
CRMP2_IR	Based on Mean	.129	1	4	.737
	Based on Median	.035	1	4	.860
	Based on Median and with adjusted df	.035	1	3.996	.860
	Based on trimmed mean	.115	1	4	.751

Tests the null hypothesis that the error variance of the dependent variable is equal across groups.[a,b]

a. Dependent variable: CRMP2_IR

b. Design: Intercept + Treatment

Tests of Between-Subjects Effects

Dependent Variable: CRMP2_IR

Source	Type III Sum of Squares	df	Mean Square	F	Sig.
Corrected Model	1293.070[a]	1	1293.070	4.362	.105
Intercept	43676.682	1	43676.682	147.341	.000
Treatment	1293.070	1	1293.070	4.362	.105
Error	1185.734	4	296.434		
Total	46155.486	6			
Corrected Total	2478.805	5			

a. R Squared = .522 (Adjusted R Squared = .402)

```
EXAMINE VARIABLES=ZRE_1
  /PLOT HISTOGRAM NPPLOT
  /STATISTICS DESCRIPTIVES
  /CINTERVAL 95
  /MISSING LISTWISE
  /NOTOTAL.
```

Explore

Notes

Output Created		13-MAR-2018 18:26:06
Comments		
Input	Active Dataset	DataSet3
	Filter	<none>
	Weight	<none>
	Split File	<none>
	N of Rows in Working Data File	6
Missing Value Handling	Definition of Missing	User-defined missing values for dependent variables are treated as missing.
	Cases Used	Statistics are based on cases with no missing values for any dependent variable or factor used.

Syntax		EXAMINE VARIABLES=ZRE_1<br>/PLOT HISTOGRAM NPPLOT<br>/STATISTICS DESCRIPTIVES<br>/CINTERVAL 95<br>/MISSING LISTWISE<br>/NOTOTAL.
Resources	Processor Time	00:00:00.31
	Elapsed Time	00:00:00.29

Case Processing Summary

	Cases					
	Valid		Missing		Total	
	N	Percent	N	Percent	N	Percent
Standardized Residual for CRMP2_IR	6	100.0%	0	0.0%	6	100.0%

Descriptives

			Statistic	Std. Error
Standardized Residual for CRMP2_IR	Mean		.0000	.36515
	95% Confidence Interval for Mean	Lower Bound	-.9386	
		Upper Bound	.9386	
	5% Trimmed Mean		-.0230	
	Median		-.4546	
	Variance		.800	
	Std. Deviation		.89443	
	Minimum		-.81	
	Maximum		1.22	
	Range		2.03	
	Interquartile Range		1.73	
	Skewness		.886	.845
	Kurtosis		-1.772	1.741

Tests of Normality

	Kolmogorov-Smirnov[a]			Shapiro-Wilk		
	Statistic	df	Sig.	Statistic	df	Sig.
Standardized Residual for CRMP2_IR	.345	6	.025	.780	6	.038

a. Lilliefors Significance Correction

Standardized Residual for CRMP2_IR

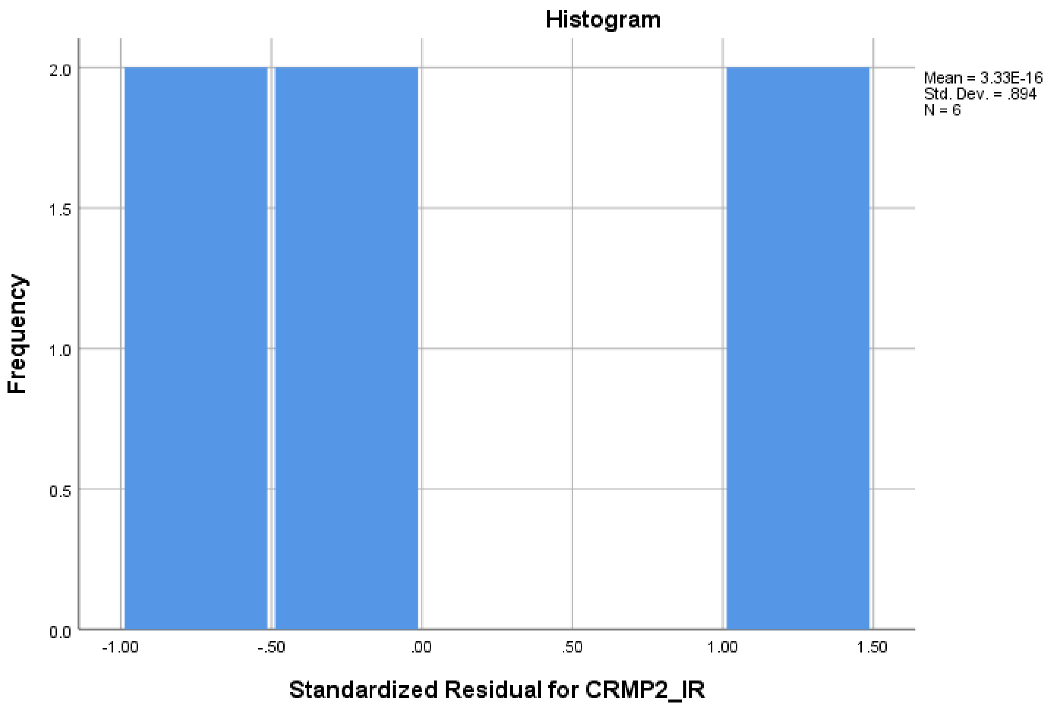

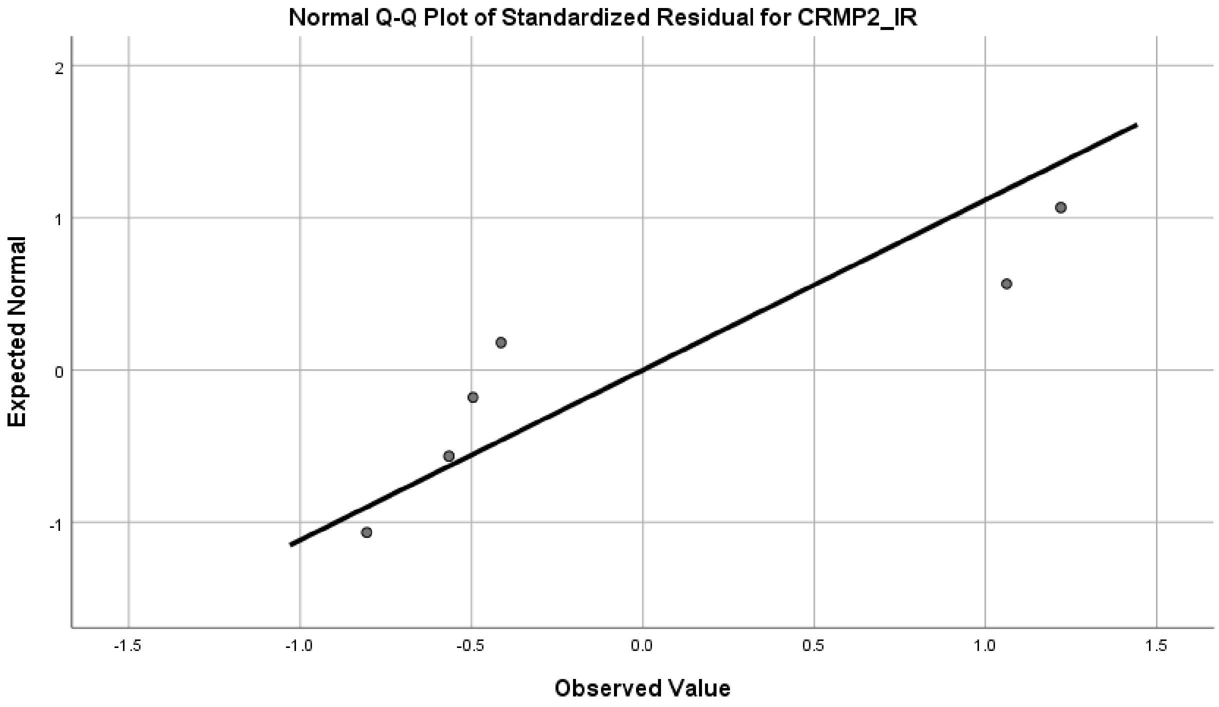

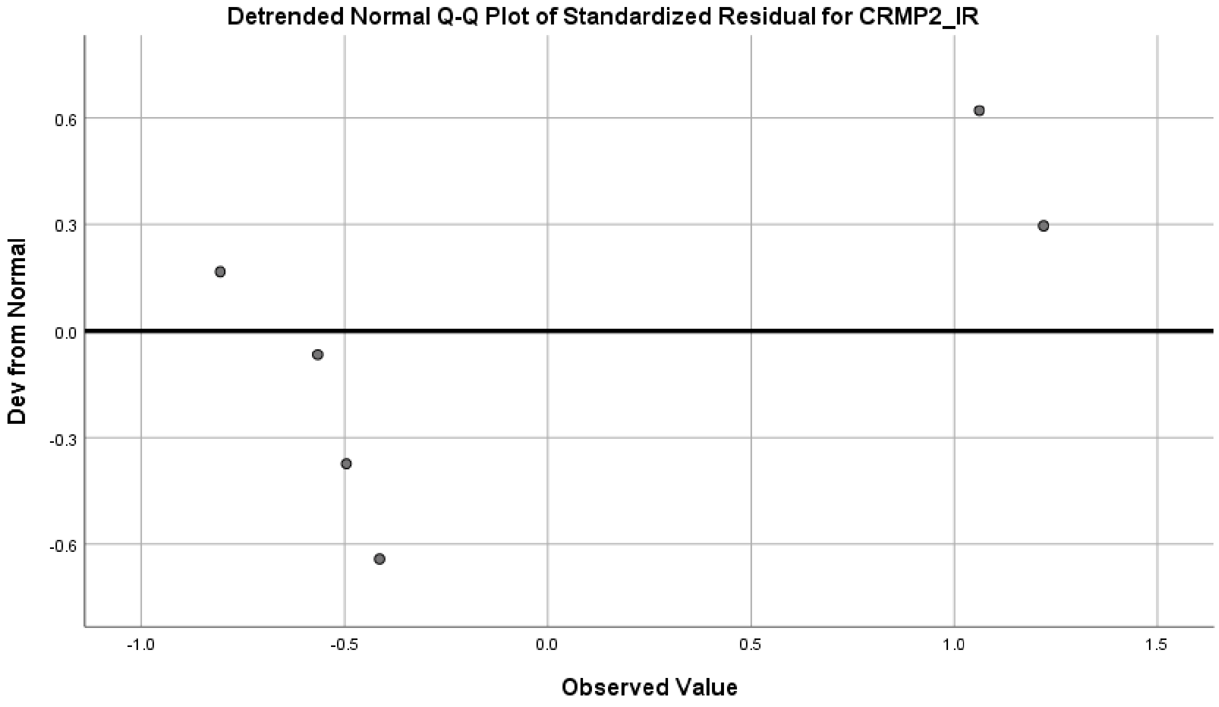


```
UNIANOVA Panx1_IR BY Treatment
  /METHOD=SSTYPE(3)
```

```
/INTERCEPT=INCLUDE
/SAVE=ZRESID
/PRINT DESCRIPTIVE HOMOGENEITY
/CRITERIA=ALPHA(.05)
/DESIGN=Treatment.
```

Univariate Analysis of Variance

Notes

Output Created		13-MAR-2018 18:46:48
Comments		
Input	Active Dataset	DataSet3
	Filter	<none>
	Weight	<none>
	Split File	<none>
	N of Rows in Working Data File	6
Missing Value Handling	Definition of Missing	User-defined missing values are treated as missing.
	Cases Used	Statistics are based on all cases with valid data for all variables in the model.
Syntax		UNIANOVA Panx1_IR BY Treatment /METHOD=SSTYPE(3) /INTERCEPT=INCLUDE /SAVE=ZRESID /PRINT DESCRIPTIVE HOMOGENEITY /CRITERIA=ALPHA(.05) /DESIGN=Treatment.
Resources	Processor Time	00:00:00.00
	Elapsed Time	00:00:00.01
Variables Created or Modified	ZRE_2	Standardized Residual for Panx1_IR

Between-Subjects Factors

		Value Label	N
Treatment	1.00	Vehicle	3
	2.00	Probenecid	3

Descriptive Statistics

Dependent Variable: Panx1_IR

Treatment	Mean	Std. Deviation	N
Vehicle	100.0000	27.73057	3
Probenecid	81.8343	33.27576	3
Total	90.9172	29.14622	6

Levene's Test of Equality of Error Variances[a,b]

		Levene Statistic	df1	df2	Sig.
Panx1_IR	Based on Mean	.178	1	4	.695
	Based on Median	.046	1	4	.840
	Based on Median and with adjusted df	.046	1	3.999	.840
	Based on trimmed mean	.158	1	4	.711

Tests the null hypothesis that the error variance of the dependent variable is equal across groups.[a,b]

a. Dependent variable: Panx1_IR

b. Design: Intercept + Treatment

Tests of Between-Subjects Effects

Dependent Variable: Panx1_IR

Source	Type III Sum of Squares	df	Mean Square	F	Sig.
Corrected Model	494.989[a]	1	494.989	.528	.508
Intercept	49595.585	1	49595.585	52.866	.002
Treatment	494.989	1	494.989	.528	.508
Error	3752.521	4	938.130		

Total	53843.095	6			
Corrected Total	4247.510	5			

a. R Squared = .117 (Adjusted R Squared = -.104)

```
EXAMINE VARIABLES=ZRE_2
  /PLOT HISTOGRAM NPPLOT
  /STATISTICS DESCRIPTIVES
  /CINTERVAL 95
  /MISSING LISTWISE
  /NOTOTAL.
```

Explore

Notes

Output Created		13-MAR-2018 18:47:12
Comments		
Input	Active Dataset	DataSet3
	Filter	<none>
	Weight	<none>
	Split File	<none>
	N of Rows in Working Data File	6
Missing Value Handling	Definition of Missing	User-defined missing values for dependent variables are treated as missing.
	Cases Used	Statistics are based on cases with no missing values for any dependent variable or factor used.

Syntax		EXAMINE VARIABLES=ZRE_2<br>/PLOT HISTOGRAM NPPLOT<br>/STATISTICS DESCRIPTIVES<br>/CINTERVAL 95<br>/MISSING LISTWISE<br>/NOTOTAL.
Resources	Processor Time	00:00:00.31
	Elapsed Time	00:00:00.28

Case Processing Summary

	Cases					
	Valid		Missing		Total	
	N	Percent	N	Percent	N	Percent
Standardized Residual for Panx1_IR	6	100.0%	0	0.0%	6	100.0%

Descriptives

			Statistic	Std. Error
Standardized Residual for Panx1_IR	Mean		.0000	.36515
	95% Confidence Interval for Mean	Lower Bound	-.9386	
		Upper Bound	.9386	
	5% Trimmed Mean		.0104	
	Median		-.0395	
	Variance		.800	
	Std. Deviation		.89443	
	Minimum		-1.23	
	Maximum		1.04	
	Range		2.28	
	Interquartile Range		1.60	
	Skewness		-.163	.845
	Kurtosis		-1.733	1.741

Tests of Normality

	Kolmogorov-Smirnov[a]			Shapiro-Wilk		
	Statistic	df	Sig.	Statistic	df	Sig.
Standardized Residual for Panx1_IR	.207	6	.200*	.931	6	.590

*. This is a lower bound of the true significance.

a. Lilliefors Significance Correction

Standardized Residual for Panx1_IR

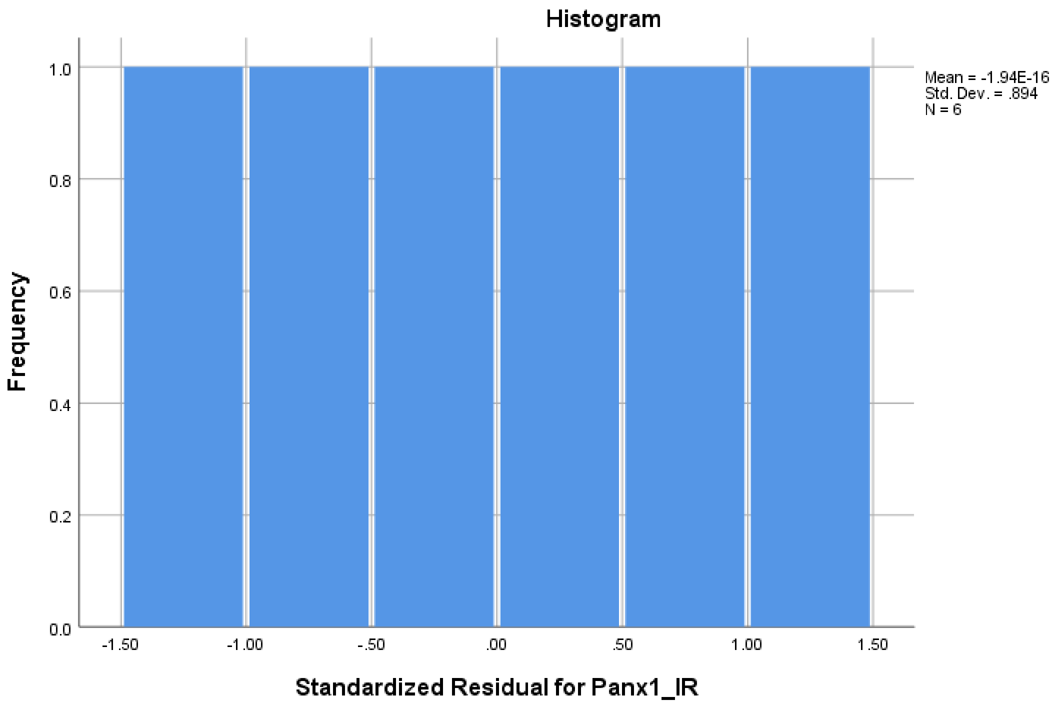

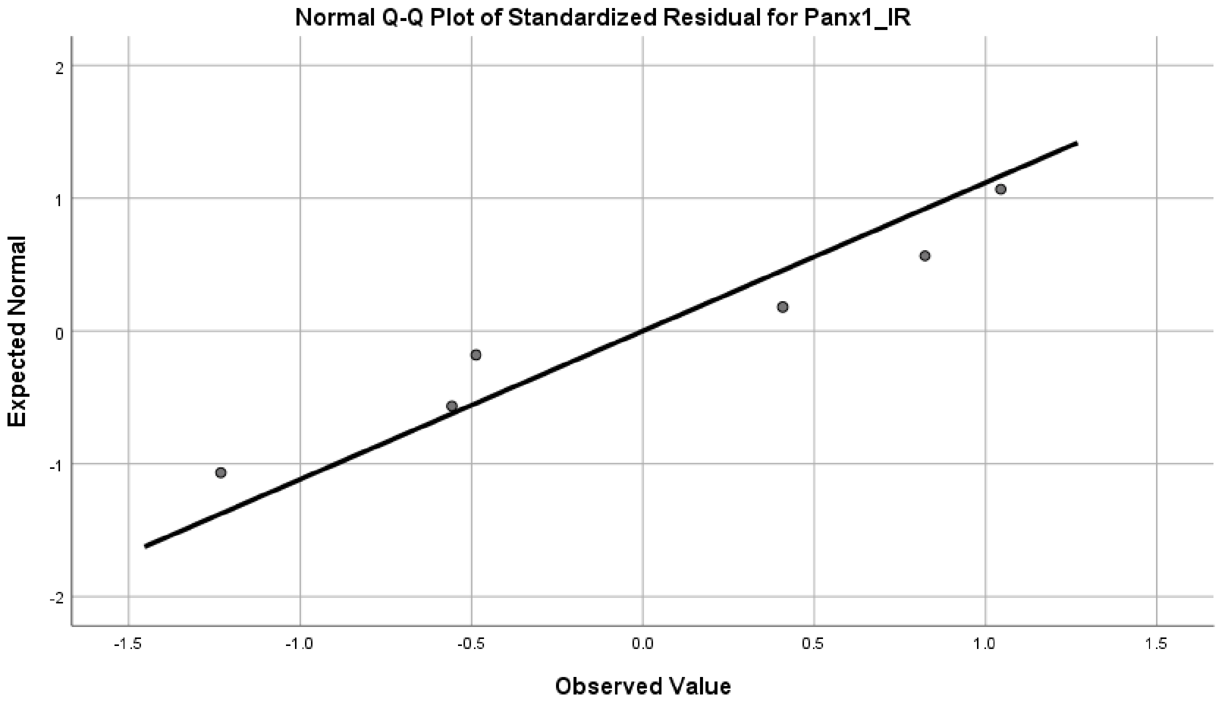
Normal Q-Q Plot of Standardized Residual for Panx1_IR
Expected Normal
Observed Value
2
1
0
-1
-2
-1.5
-1.0
-0.5
0.0
0.5
1.0
1.5

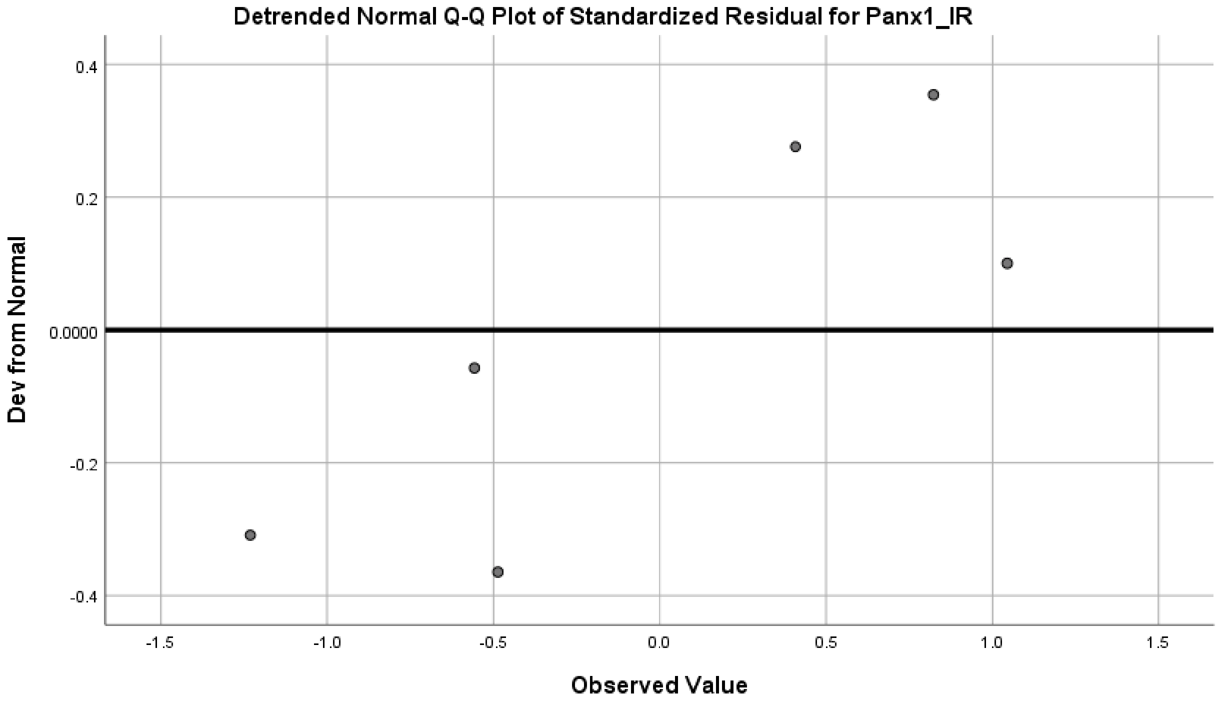
Detrended Normal Q-Q Plot of Standardized Residual for Panx1_IR
Dev from Normal
Observed Value
0.4
0.2
0.0000
-0.2
-0.4
-1.5
-1.0
-0.5
0.0
0.5
1.0
1.5

```
GET DATA
```

```
  /TYPE=XLSX
  /FILE='Z:\Swayne Lab\Manuscripts\2018 Frontiers Panx1-Crmp2
paper(dropbox)\Deep statistical analysis\CRMP2 and Panx1 WB x
treatment\CRMP2_Panx1_Expression_by_treatment.xlsx'
  /SHEET=name 'CRMP2_Panx1_Expression_by_treat'
  /CELLRANGE=FULL
  /READNAMES=ON
  /DATATYPEMIN PERCENTAGE=95.0
  /HIDDEN IGNORE=YES.
EXECUTE.
DATASET NAME DataSet1 WINDOW=FRONT.
*Nonparametric Tests: Independent Samples.
NPTESTS
  /INDEPENDENT TEST (CRMP2_IR) GROUP (Treatment)
  /MISSING SCOPE=ANALYSIS USERMISSING=EXCLUDE
  /CRITERIA ALPHA=0.05  CILEVEL=95.
```

Nonparametric Tests

Notes

Output Created		13-MAR-2018 20:41:49
Comments		
Input	Active Dataset	DataSet1
	Filter	<none>
	Weight	<none>
	Split File	<none>
	N of Rows in Working Data File	6
Syntax		NPTESTS /INDEPENDENT TEST (CRMP2_IR) GROUP (Treatment) /MISSING SCOPE=ANALYSIS USERMISSING=EXCLUDE /CRITERIA ALPHA=0.05 CILEVEL=95.
Resources	Processor Time	00:00:00.92
	Elapsed Time	00:00:01.09

[DataSet1]

null : null

Hypothesis Test Summary

	Null Hypothesis	Test	Sig.	Decision
1	The distribution of CRMP2_IR is the same across categories of Treatment.	Independent-Samples Mann-Whitney U Test	.200[1]	Retain the null hypothesis.

Asymptotic significances are displayed. The significance level is .05.

[1] Exact significance is displayed for this test.

Proximity ligation Assay Experiments

```
EXAMINE VARIABLES=PLA_F BY Treatment
  /PLOT HISTOGRAM NPPLOT
  /STATISTICS DESCRIPTIVES
  /CINTERVAL 95
  /MISSING LISTWISE
  /NOTOTAL.
```

Explore

Notes

Output Created		13-APR-2018 12:11:15
Comments		
Input	Active Dataset	DataSet0
	Filter	<none>
	Weight	<none>
	Split File	<none>
	N of Rows in Working Data File	147
Missing Value Handling	Definition of Missing	User-defined missing values for dependent variables are treated as missing.
	Cases Used	Statistics are based on cases with no missing values for any dependent variable or factor used.
Syntax		EXAMINE VARIABLES=PLA_F BY Treatment<br>/PLOT HISTOGRAM NPPLOT<br>/STATISTICS DESCRIPTIVES<br>/CINTERVAL 95<br>/MISSING LISTWISE<br>/NOTOTAL.

Resources	Processor Time	00:00:01.19
	Elapsed Time	00:00:01.04

Treatment

Case Processing Summary

		Cases					
		Valid		Missing		Total	
	Treatment	N	Percent	N	Percent	N	Percent
PLA_F	Vehicle	77	100.0%	0	0.0%	77	100.0%
	Probenecid	70	100.0%	0	0.0%	70	100.0%

Descriptives

	Treatment			Statistic	Std. Error
PLA_F	Vehicle	Mean		25.9746	2.69207
		95% Confidence Interval for Mean	Lower Bound	20.6129	
			Upper Bound	31.3363	
		5% Trimmed Mean		23.5773	
		Median		14.6393	
		Variance		558.038	
		Std. Deviation		23.62284	
		Minimum		3.23	
		Maximum		108.47	
		Range		105.24	
		Interquartile Range		30.84	
		Skewness		1.439	.274
		Kurtosis		1.655	.541
	Probenecid	Mean		17.1189	2.04607
		95% Confidence Interval for Mean	Lower Bound	13.0371	
			Upper Bound	21.2007	
		5% Trimmed Mean		15.0845	
		Median		9.8518	

	Variance	293.048	
	Std. Deviation	17.11864	
	Minimum	3.47	
	Maximum	89.45	
	Range	85.98	
	Interquartile Range	14.83	
	Skewness	2.067	.287
	Kurtosis	4.447	.566

Tests of Normality

		Kolmogorov-Smirnov[a]			Shapiro-Wilk		
	Treatment	Statistic	df	Sig.	Statistic	df	Sig.
PLA_F	Vehicle	.234	77	.000	.818	77	.000
	Probenecid	.258	70	.000	.727	70	.000

a. Lilliefors Significance Correction

PLA_F

Histograms

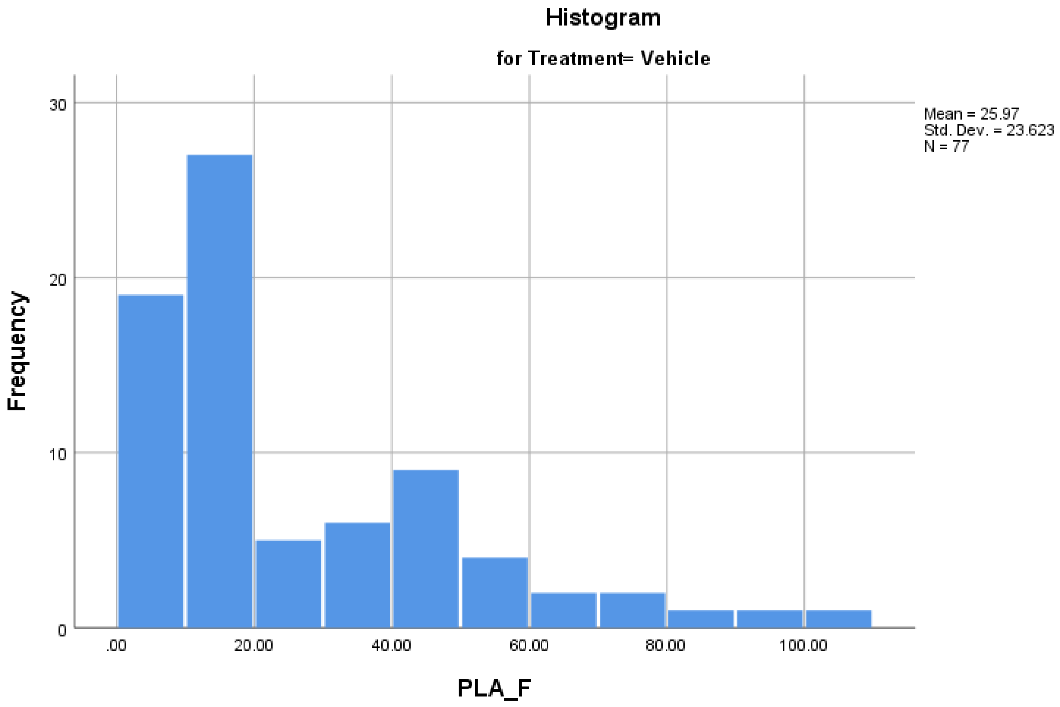
Histogram
for Treatment= Vehicle
Mean = 25.97
Std. Dev. = 23.623
N = 77
Frequency
30
20
10
0
.00
20.00
40.00
60.00
80.00
100.00
PLA_F

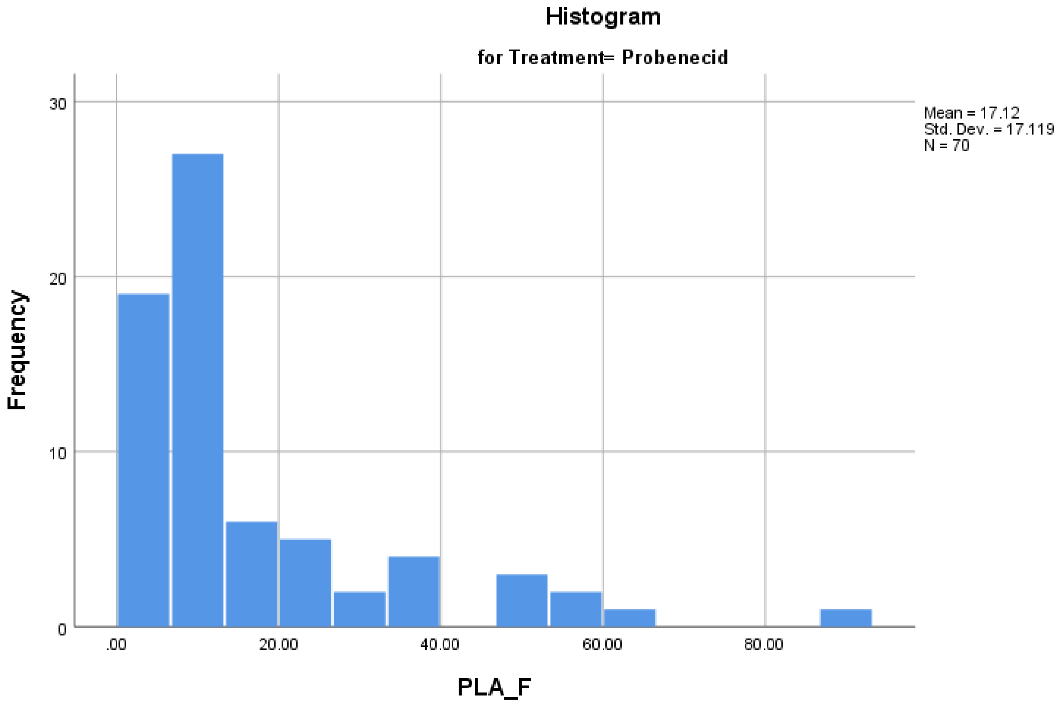
Histogram
for Treatment= Probenecid
Mean = 17.12
Std. Dev. = 17.119
N = 70
Frequency
30
20
10
0
.00
20.00
40.00
60.00
80.00
PLA_F

Normal Q-Q Plots

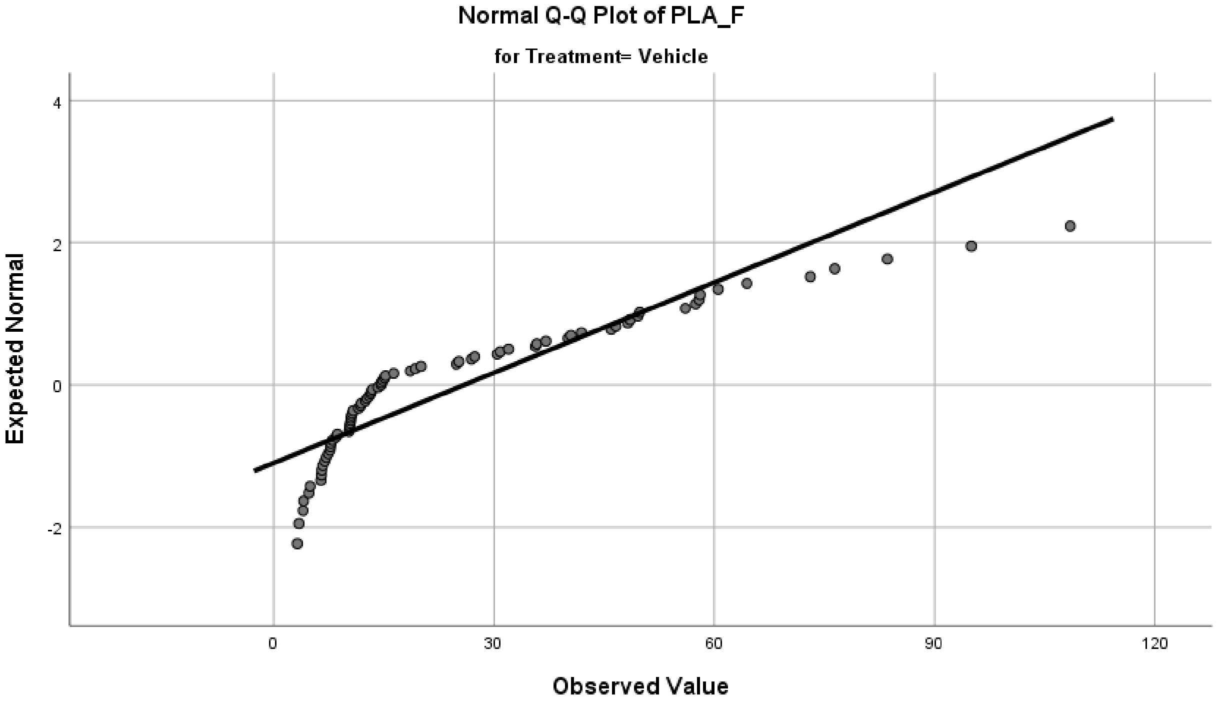

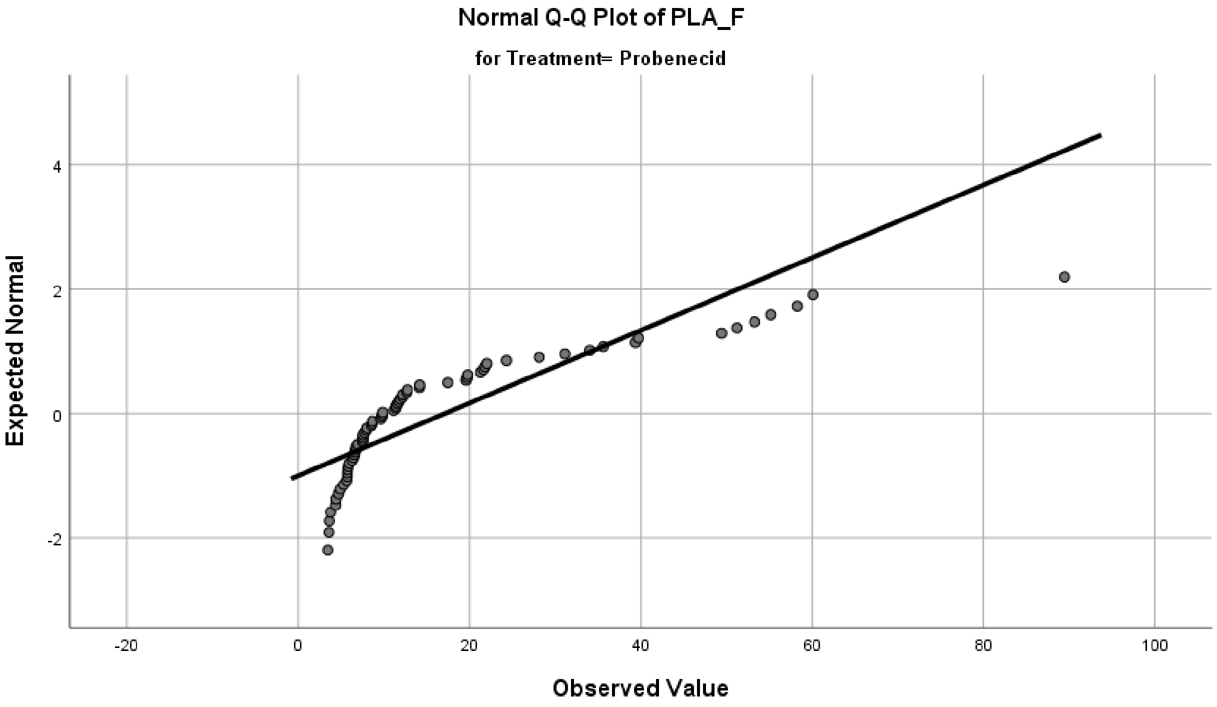

Detrended Normal Q-Q Plots

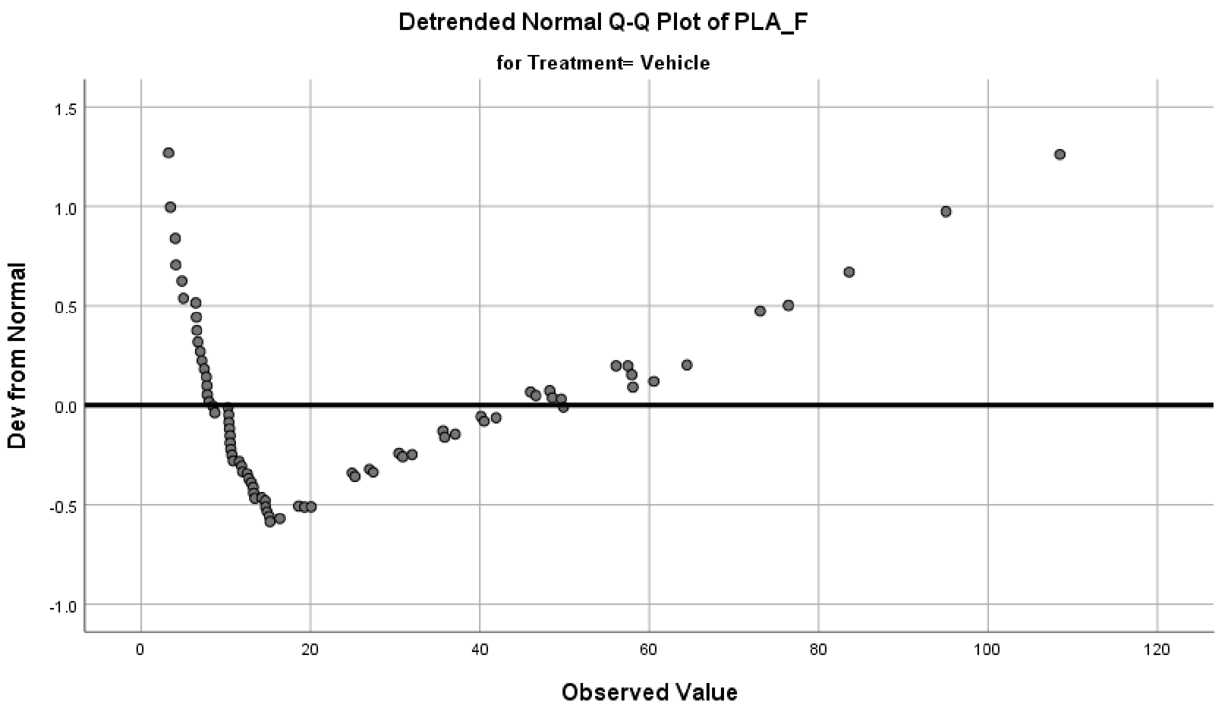

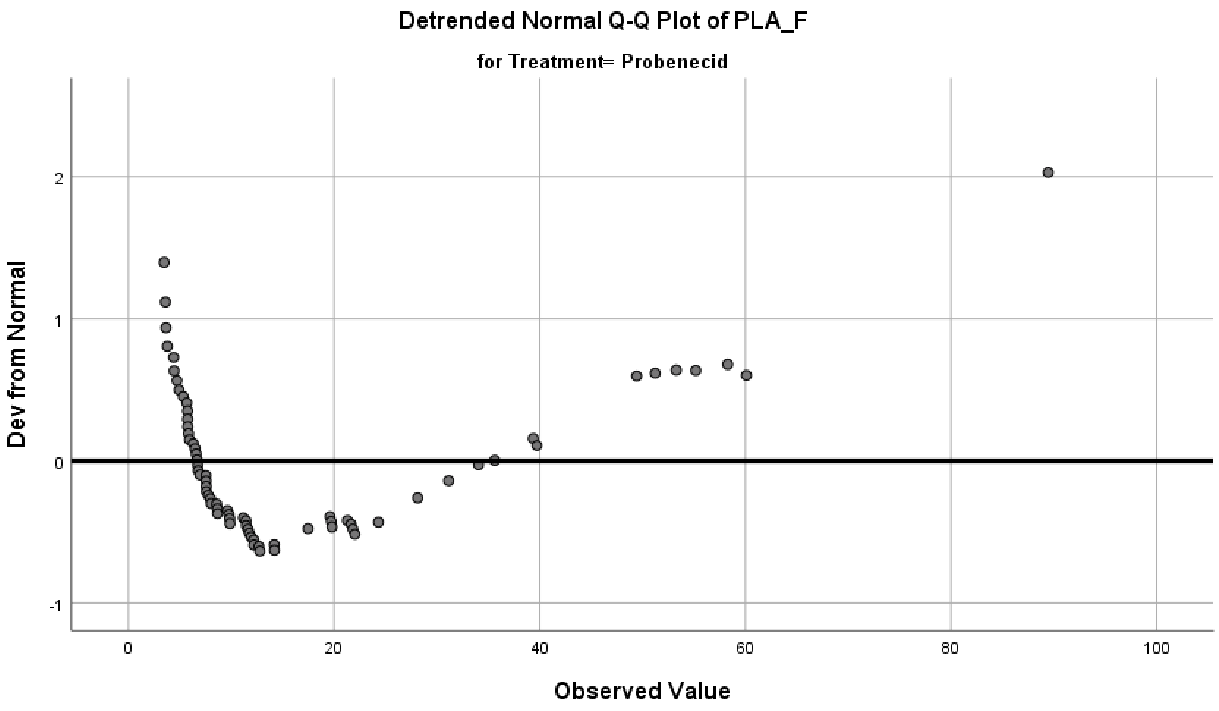


```
UNIANOVA PLA_F BY Treatment
```

```
/METHOD=SSTYPE(3)
/INTERCEPT=INCLUDE
/SAVE=ZRESID
/PRINT HOMOGENEITY
/CRITERIA=ALPHA(.05)
/DESIGN=Treatment.
```

Univariate Analysis of Variance

Notes

Output Created		13-APR-2018 12:14:15
Comments		
Input	Active Dataset	DataSet0
	Filter	<none>
	Weight	<none>
	Split File	<none>
	N of Rows in Working Data File	147
Missing Value Handling	Definition of Missing	User-defined missing values are treated as missing.
	Cases Used	Statistics are based on all cases with valid data for all variables in the model.
Syntax		UNIANOVA PLA_F BY Treatment /METHOD=SSTYPE(3) /INTERCEPT=INCLUDE /SAVE=ZRESID /PRINT HOMOGENEITY /CRITERIA=ALPHA(.05) /DESIGN=Treatment.
Resources	Processor Time	00:00:00.00
	Elapsed Time	00:00:00.03
Variables Created or Modified	ZRE_1	Standardized Residual for PLA_F

Between-Subjects Factors

		Value Label	N
Treatment	1	Vehicle	77
	2	Probenecid	70

Levene's Test of Equality of Error Variances[a,b]

		Levene Statistic	df1	df2	Sig.
PLA_F	Based on Mean	9.460	1	145	.003
	Based on Median	4.530	1	145	.035
	Based on Median and with adjusted df	4.530	1	135.818	.035
	Based on trimmed mean	8.660	1	145	.004

Tests the null hypothesis that the error variance of the dependent variable is equal across groups.

a. Dependent variable: PLA_F

b. Design: Intercept + Treatment

Tests of Between-Subjects Effects

Dependent Variable: PLA_F

Source	Type III Sum of Squares	df	Mean Square	F	Sig.
Corrected Model	2875.487[a]	1	2875.487	6.657	.011
Intercept	68091.871	1	68091.871	157.642	.000
Treatment	2875.487	1	2875.487	6.657	.011
Error	62631.217	145	431.939		
Total	135095.537	147			
Corrected Total	65506.704	146			

a. R Squared = .044 (Adjusted R Squared = .037)

```
EXAMINE VARIABLES=ZRE_1 BY Treatment
  /PLOT HISTOGRAM NPPLOT
  /STATISTICS DESCRIPTIVES
  /CINTERVAL 95
  /MISSING LISTWISE
  /NOTOTAL.
```

Explore

Notes

Output Created		13-APR-2018 12:14:33
Comments		
Input	Active Dataset	DataSet0
	Filter	<none>
	Weight	<none>
	Split File	<none>
	N of Rows in Working Data File	147
Missing Value Handling	Definition of Missing	User-defined missing values for dependent variables are treated as missing.
	Cases Used	Statistics are based on cases with no missing values for any dependent variable or factor used.
Syntax		EXAMINE VARIABLES=ZRE_1 BY Treatment<br>/PLOT HISTOGRAM NPPLOT<br>/STATISTICS DESCRIPTIVES<br>/CINTERVAL 95<br>/MISSING LISTWISE<br>/NOTOTAL.
Resources	Processor Time	00:00:01.31
	Elapsed Time	00:00:01.01

Treatment

Case Processing Summary

	Treatment	Cases					
		Valid		Missing		Total	
		N	Percent	N	Percent	N	Percent
Standardized Residual for PLA_F	Vehicle	77	100.0%	0	0.0%	77	100.0%
	Probenecid	70	100.0%	0	0.0%	70	100.0%

Descriptives

	Treatment			Statistic	Std. Error
Standardized Residual for PLA_F	Vehicle	Mean		.0000	.12953
		95% Confidence Interval for Mean	Lower Bound	-.2580	
			Upper Bound	.2580	
		5% Trimmed Mean		-.1153	
		Median		-.5454	
		Variance		1.292	
		Std. Deviation		1.13663	
		Minimum		-1.09	
		Maximum		3.97	
		Range		5.06	
		Interquartile Range		1.48	
		Skewness		1.439	.274
		Kurtosis		1.655	.541
	Probenecid	Mean		.0000	.09845
		95% Confidence Interval for Mean	Lower Bound	-.1964	
			Upper Bound	.1964	
		5% Trimmed Mean		-.0979	
		Median		-.3497	
		Variance		.678	
		Std. Deviation		.82368	
		Minimum		-.66	
		Maximum		3.48	
		Range		4.14	
		Interquartile Range		.71	
		Skewness		2.067	.287
		Kurtosis		4.447	.566

Tests of Normality

	Treatment	Kolmogorov-Smirnov[a] Statistic	df	Sig.	Shapiro-Wilk Statistic	df	Sig.
Standardized Residual for PLA_F	Vehicle	.234	77	.000	.818	77	.000
	Probenecid	.258	70	.000	.727	70	.000

a. Lilliefors Significance Correction

Standardized Residual for PLA_F

Histograms

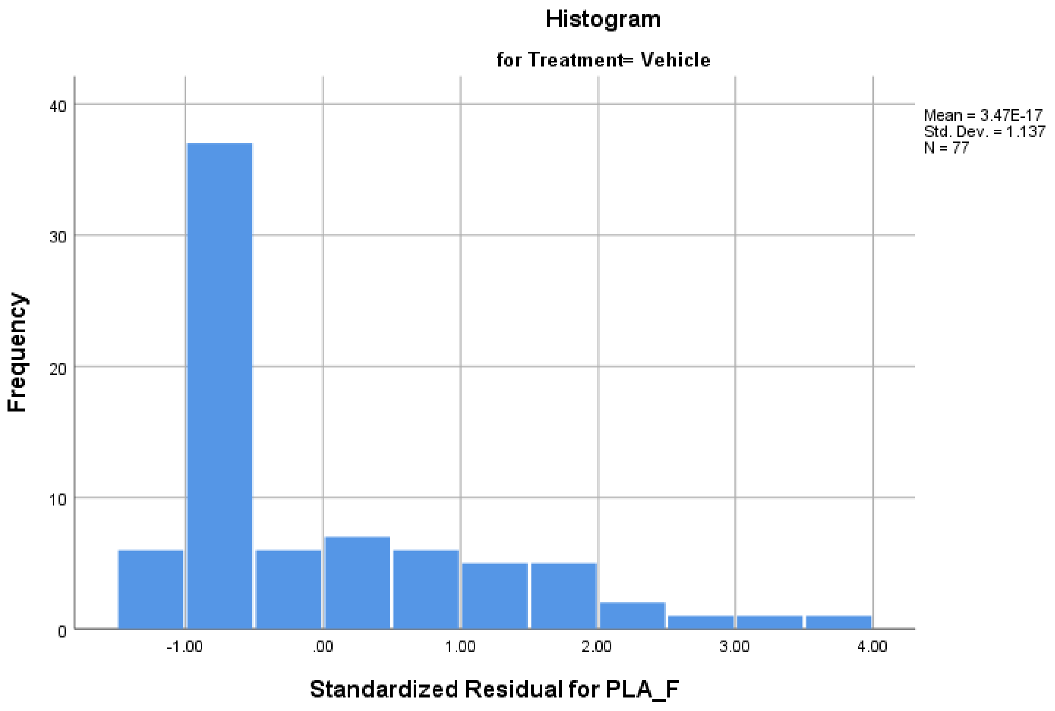

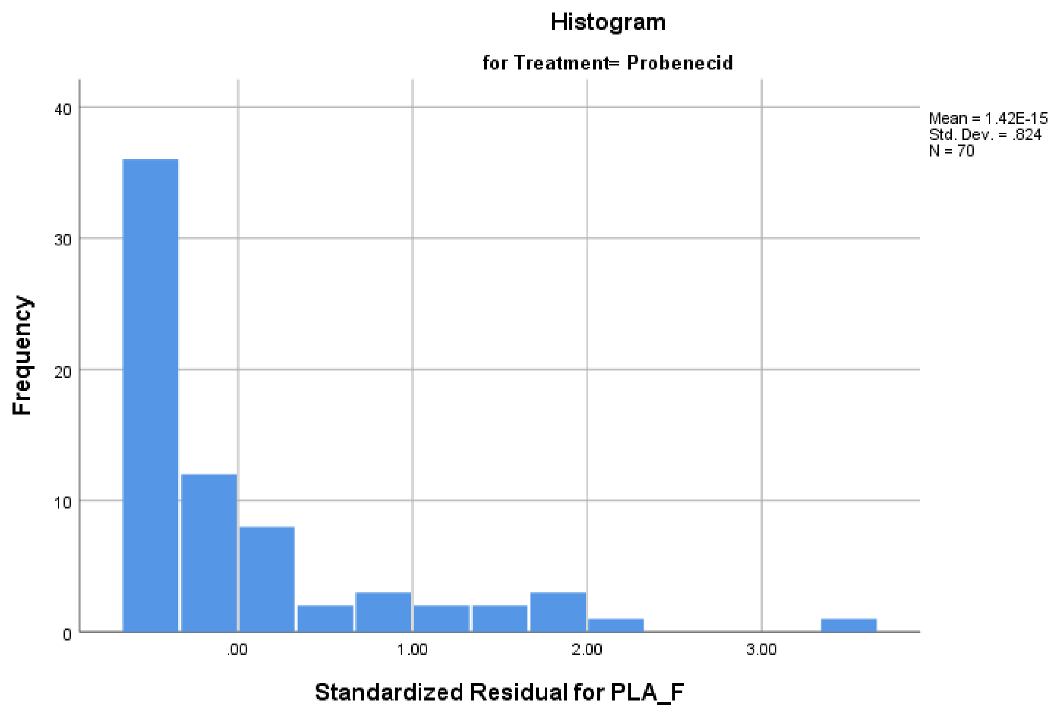

Normal Q-Q Plots

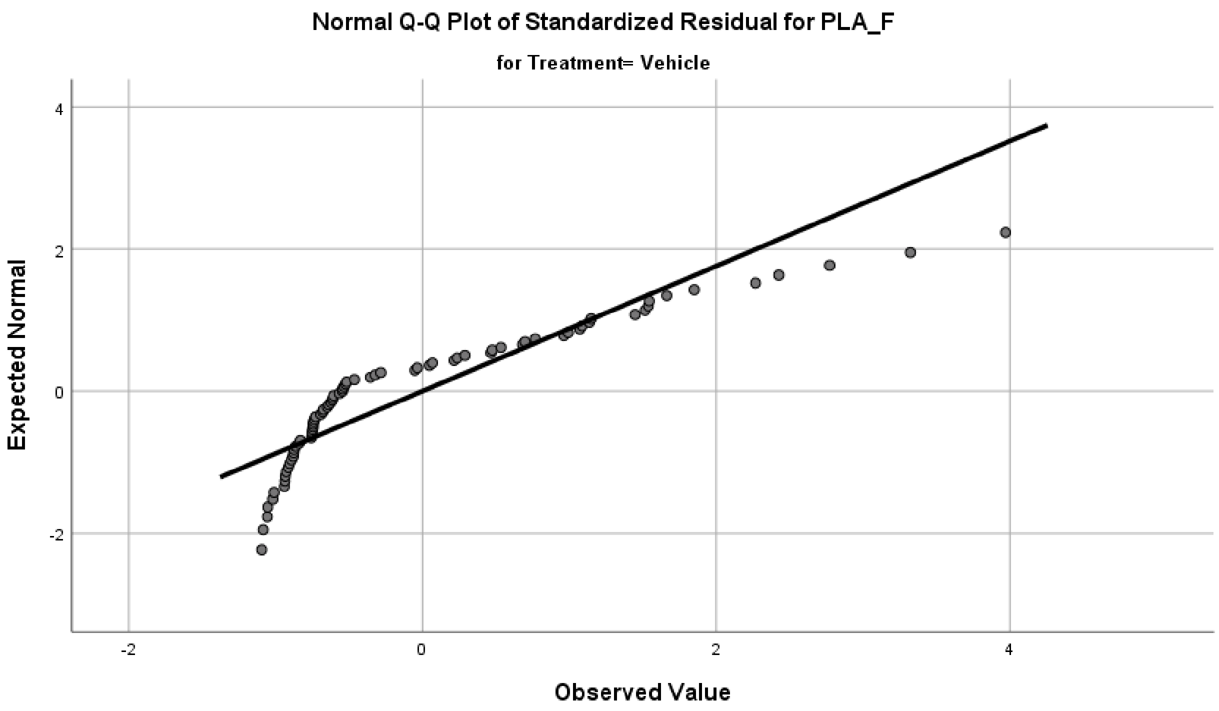
Normal Q-Q Plot of Standardized Residual for PLA_F
for Treatment= Vehicle
Expected Normal
Observed Value
4
2
0
-2
-2
0
2
4

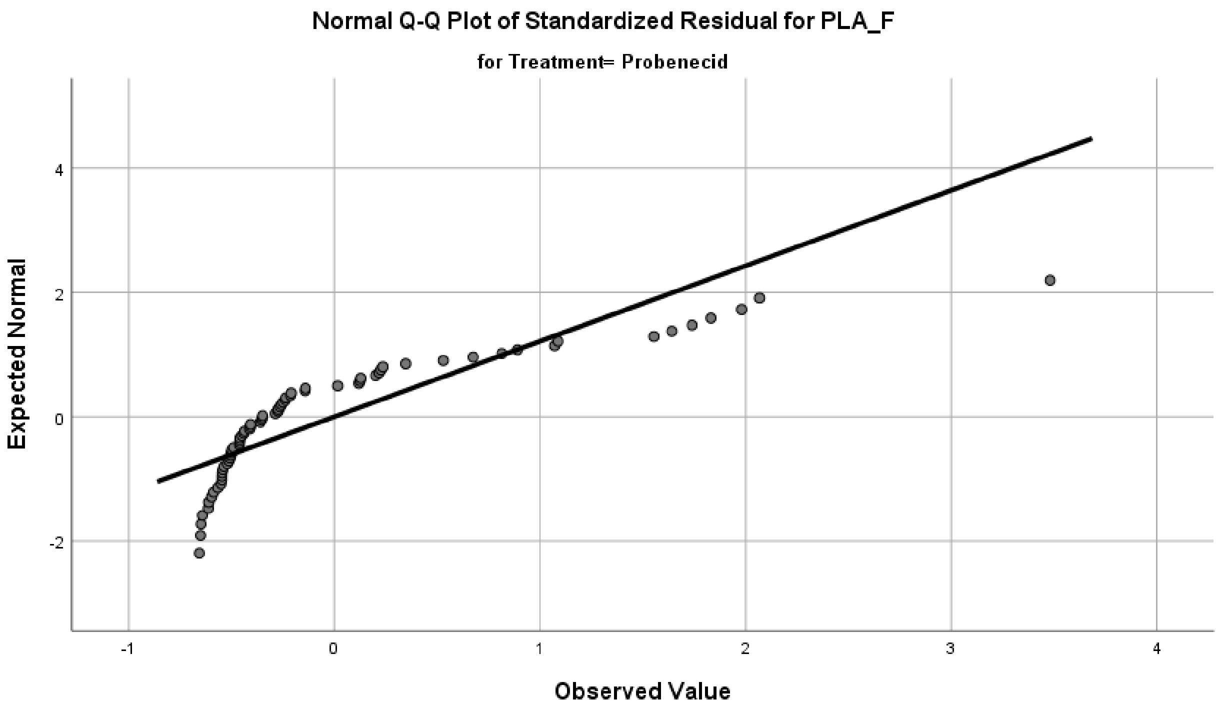
Normal Q-Q Plot of Standardized Residual for PLA_F
for Treatment= Probenecid
Expected Normal
Observed Value
4
2
0
-2
-1
0
1
2
3
4

Detrended Normal Q-Q Plots

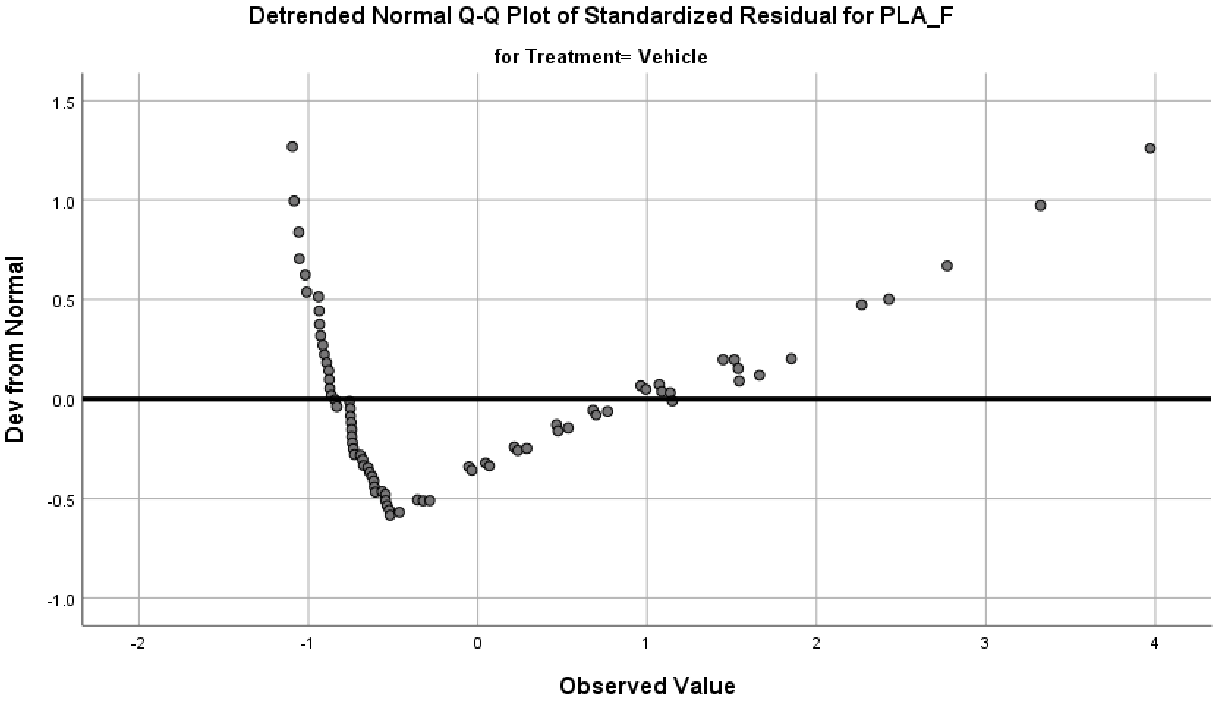

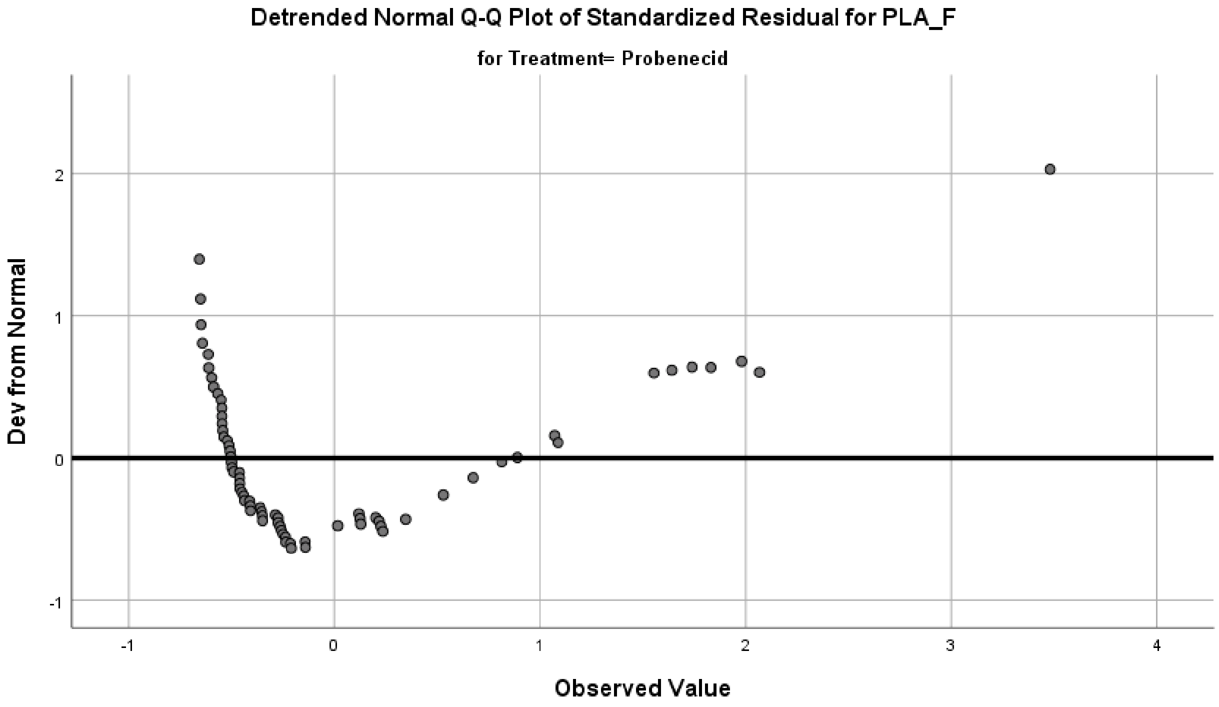


```
*Nonparametric Tests: Independent Samples.
NPTESTS
  /INDEPENDENT TEST (PLA_F) GROUP (Treatment) MANN_WHITNEY
  /MISSING SCOPE=ANALYSIS USERMISSING=EXCLUDE
  /CRITERIA ALPHA=0.05  CILEVEL=95.
```

Nonparametric Tests

Notes

Output Created		13-APR-2018 12:16:54
Comments		
Input	Active Dataset	DataSet0
	Filter	<none>
	Weight	<none>

	Split File	<none>
	N of Rows in Working Data File	147
Syntax		NPTESTS<br>/INDEPENDENT TEST<br>(PLA_F) GROUP (Treatment)<br>MANN_WHITNEY<br>/MISSING<br>SCOPE=ANALYSIS<br>USERMISSING=EXCLUDE<br>/CRITERIA ALPHA=0.05<br>CILEVEL=95.
Resources	Processor Time	00:00:00.19
	Elapsed Time	00:00:00.14

null : null

Hypothesis Test Summary

	Null Hypothesis	Test	Sig.	Decision
1	The distribution of PLA_F is the same across categories of Treatment.	Independent-Samples Mann-Whitney U Test	.004	Reject the null hypothesis.

Asymptotic significances are displayed. The significance level is .05.

Crmp2 Immunofluorescence in neurites

```
EXAMINE VARIABLES=CRMP2_IR
  /PLOT HISTOGRAM NPPLOT
  /STATISTICS DESCRIPTIVES
  /CINTERVAL 95
  /MISSING LISTWISE
  /NOTOTAL.
```

Explore

Notes

Output Created		14-MAR-2018 08:46:36
Comments		
Input	Active Dataset	DataSet0
	Filter	<none>

	Weight	<none>
	Split File	<none>
	N of Rows in Working Data File	116
Missing Value Handling	Definition of Missing	User-defined missing values for dependent variables are treated as missing.
	Cases Used	Statistics are based on cases with no missing values for any dependent variable or factor used.
Syntax		EXAMINE VARIABLES=CRMP2_IR /PLOT HISTOGRAM NPPLOT /STATISTICS DESCRIPTIVES /CINTERVAL 95 /MISSING LISTWISE /NOTOTAL.
Resources	Processor Time	00:00:01.78
	Elapsed Time	00:00:01.22

```
[DataSet0]
```

Case Processing Summary

	Cases					
	Valid		Missing		Total	
	N	Percent	N	Percent	N	Percent
CRMP2_IR	116	100.0%	0	0.0%	116	100.0%

Descriptives

			Statistic	Std. Error
CRMP2_IR	Mean		1.947251	.1553927
	95% Confidence Interval for	Lower Bound	1.639448	

Mean	Upper Bound	2.255054	
5% Trimmed Mean		1.720945	
Median		1.515193	
Variance		2.801	
Std. Deviation		1.6736309	
Minimum		.4730	
Maximum		15.2975	
Range		14.8245	
Interquartile Range		1.0466	
Skewness		5.045	.225
Kurtosis		35.665	.446

Tests of Normality

	Kolmogorov-Smirnov[a]			Shapiro-Wilk		
	Statistic	df	Sig.	Statistic	df	Sig.
CRMP2_IR	.213	116	.000	.571	116	.000

a. Lilliefors Significance Correction

CRMP2_IR

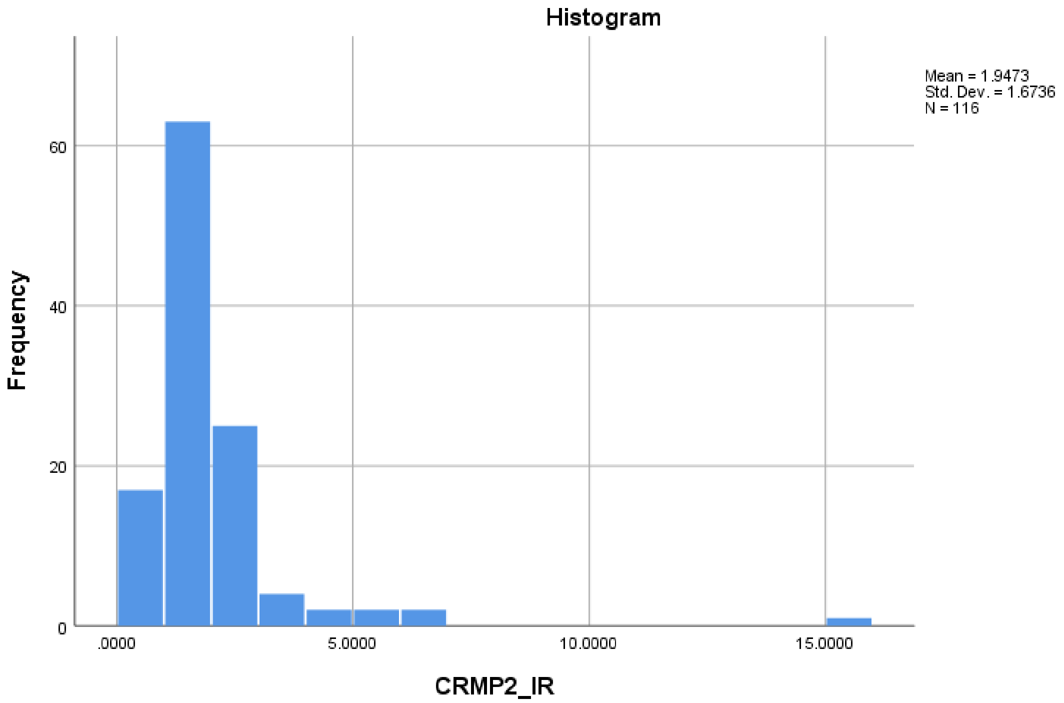
Histogram
Mean = 1.9473
Std. Dev. = 1.6736
N = 116
Frequency
60
40
20
0
.0000
5.0000
10.0000
15.0000
CRMP2_IR

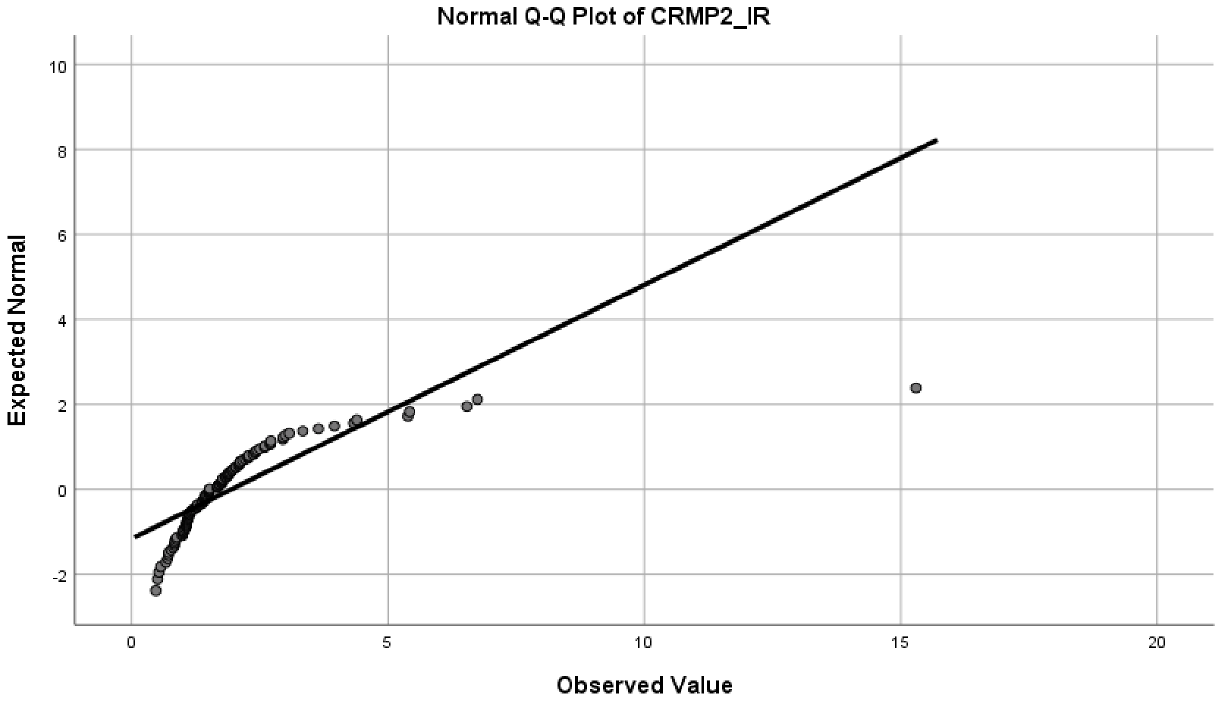
Normal Q-Q Plot of CRMP2_IR
Expected Normal
10
8
6
4
2
0
-2
0
5
10
15
20
Observed Value

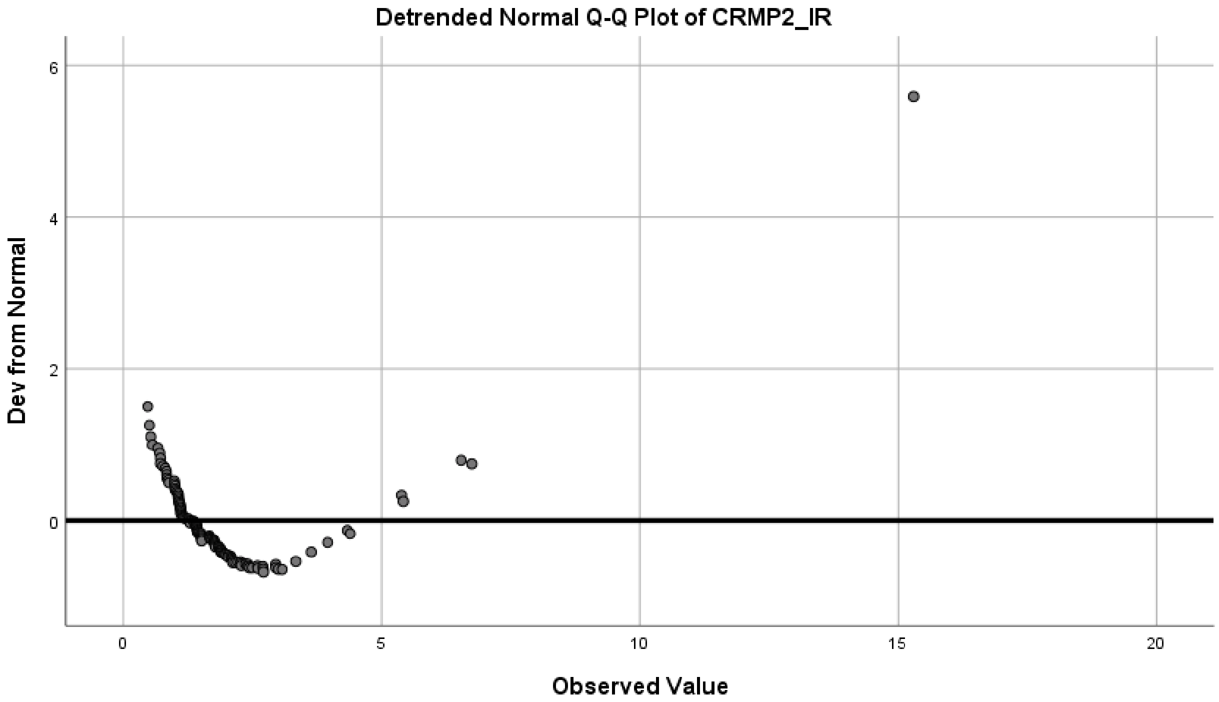


```
UNIANOVA CRMP2_IR BY Neurite_length
  /METHOD=SSTYPE(3)
  /INTERCEPT=INCLUDE
  /SAVE=ZRESID
  /PRINT DESCRIPTIVE HOMOGENEITY
  /CRITERIA=ALPHA(.05)
  /DESIGN=Neurite_length.
```

Univariate Analysis of Variance

Notes

Output Created		14-MAR-2018 08:48:57
Comments		
Input	Active Dataset	DataSet0
	Filter	<none>
	Weight	<none>
	Split File	<none>

	N of Rows in Working Data File	116
Missing Value Handling	Definition of Missing	User-defined missing values are treated as missing.
	Cases Used	Statistics are based on all cases with valid data for all variables in the model.
Syntax		UNIANOVA CRMP2_IR BY Neurite_length<br>/METHOD=SSTYPE(3)<br>/INTERCEPT=INCLUDE<br>/SAVE=ZRESID<br>/PRINT DESCRIPTIVE HOMOGENEITY<br>/CRITERIA=ALPHA(.05)<br>/DESIGN=Neurite_length.
Resources	Processor Time	00:00:00.02
	Elapsed Time	00:00:00.11
Variables Created or Modified	ZRE_1	Standardized Residual for CRMP2_IR

Between-Subjects Factors

		Value Label	N
Neurite_length	1.00	10 µm to 30 µm	38
	2.00	30 µm - 50 µm	40
	3.00	> 50 µm	38

Descriptive Statistics

Dependent Variable: CRMP2_IR

Neurite_length	Mean	Std. Deviation	N
10 µm to 30 µm	2.680620	2.5505132	38
30 µm - 50 µm	1.585712	.9996162	40
> 50 µm	1.594450	.5710230	38
Total	1.947251	1.6736309	116

Levene's Test of Equality of Error Variances[a,b]

		Levene Statistic	df1	df2	Sig.
CRMP2_IR	Based on Mean	6.861	2	113	.002
	Based on Median	3.976	2	113	.021
	Based on Median and with adjusted df	3.976	2	47.715	.025
	Based on trimmed mean	4.684	2	113	.011

Tests the null hypothesis that the error variance of the dependent variable is equal across groups.[a,b]

a. Dependent variable: CRMP2_IR

b. Design: Intercept + Neurite_length

Tests of Between-Subjects Effects

Dependent Variable: CRMP2_IR

Source	Type III Sum of Squares	df	Mean Square	F	Sig.
Corrected Model	30.396[a]	2	15.198	5.887	.004
Intercept	442.459	1	442.459	171.388	.000
Neurite_length	30.396	2	15.198	5.887	.004
Error	291.724	113	2.582		
Total	761.967	116			
Corrected Total	322.120	115			

a. R Squared = .094 (Adjusted R Squared = .078)

```
EXAMINE VARIABLES=ZRE_1
  /PLOT HISTOGRAM NPPLOT
  /STATISTICS DESCRIPTIVES
  /CINTERVAL 95
  /MISSING LISTWISE
  /NOTOTAL.
```

Explore

Notes

Output Created		14-MAR-2018 08:49:38
Comments		
Input	Active Dataset	DataSet0
	Filter	<none>
	Weight	<none>
	Split File	<none>
	N of Rows in Working Data File	116
Missing Value Handling	Definition of Missing	User-defined missing values for dependent variables are treated as missing.
	Cases Used	Statistics are based on cases with no missing values for any dependent variable or factor used.
Syntax		EXAMINE VARIABLES=ZRE_1<br>/PLOT HISTOGRAM NPPLOT<br>/STATISTICS DESCRIPTIVES<br>/CINTERVAL 95<br>/MISSING LISTWISE<br>/NOTOTAL.
Resources	Processor Time	00:00:00.53
	Elapsed Time	00:00:00.32

Case Processing Summary

	Cases					
	Valid		Missing		Total	
	N	Percent	N	Percent	N	Percent
Standardized Residual for CRMP2_IR	116	100.0%	0	0.0%	116	100.0%

Descriptives

			Statistic	Std. Error
Standardized Residual for CRMP2_IR	Mean		.0000	.09204
	95% Confidence Interval for Mean	Lower Bound	-.1823	
		Upper Bound	.1823	
	5% Trimmed Mean		-.1147	
	Median		-.1651	
	Variance		.983	
	Std. Deviation		.99127	
	Minimum		-1.22	
	Maximum		7.85	
	Range		9.07	
	Interquartile Range		.65	
	Skewness		4.790	.225
	Kurtosis		34.340	.446

Tests of Normality

	Kolmogorov-Smirnov[a]			Shapiro-Wilk		
	Statistic	df	Sig.	Statistic	df	Sig.
Standardized Residual for CRMP2_IR	.196	116	.000	.626	116	.000

a. Lilliefors Significance Correction

Standardized Residual for CRMP2_IR

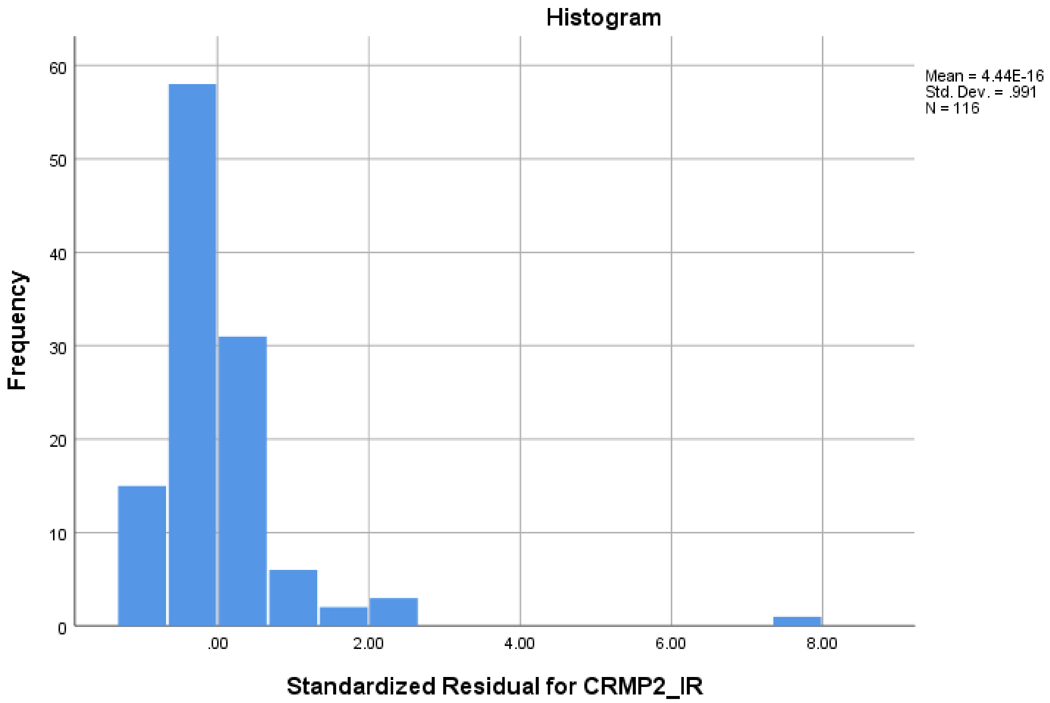
Histogram
Mean = 4.44E-16
Std. Dev. = .991
N = 116
Frequency
60
50
40
30
20
10
0
.00
2.00
4.00
6.00
8.00
Standardized Residual for CRMP2_IR

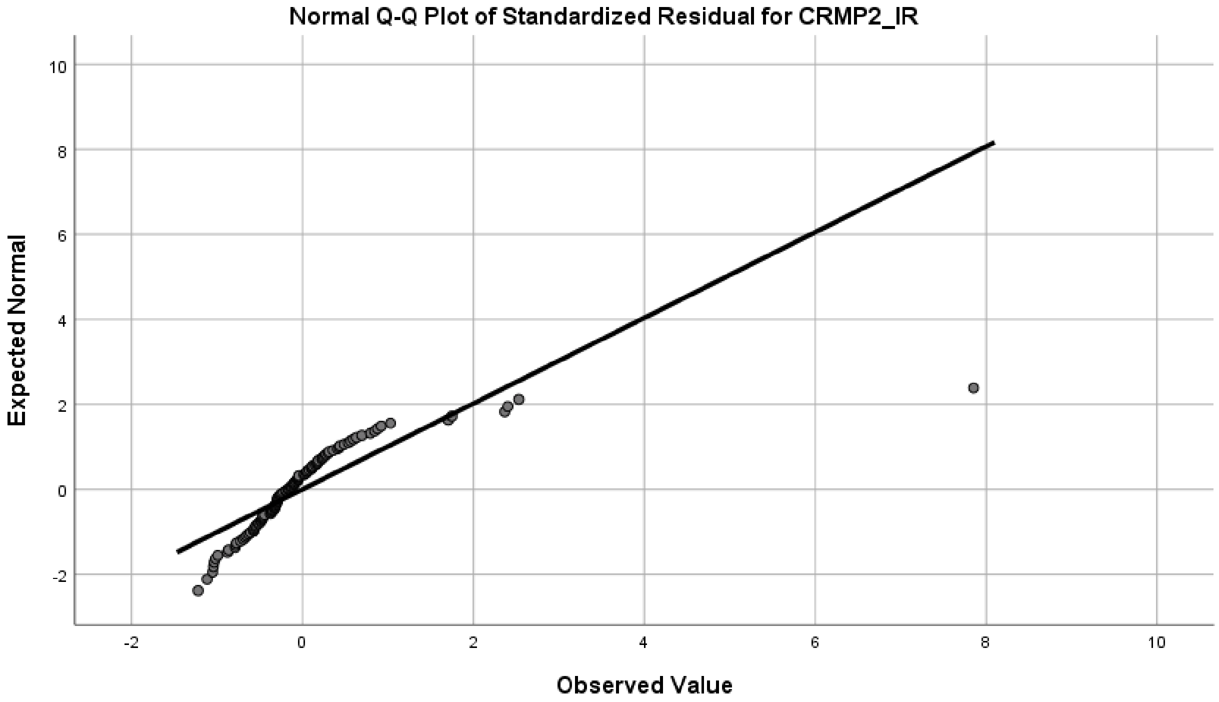
Normal Q-Q Plot of Standardized Residual for CRMP2_IR
Expected Normal
10
8
6
4
2
0
-2
-2
0
2
4
6
8
10
Observed Value

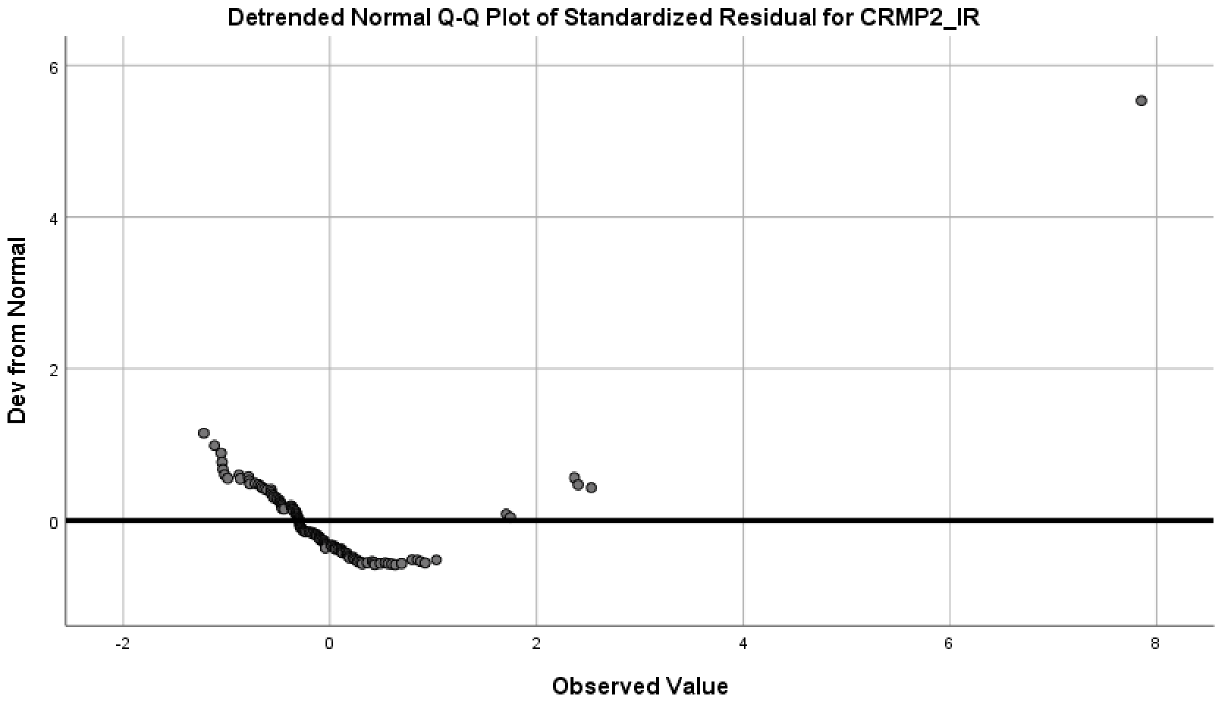


```
*Nonparametric Tests: One Sample.
NPTESTS
  /ONESAMPLE TEST (Neurite_length ZRE_1) WILCOXON(TESTVALUE=1)
  /MISSING SCOPE=ANALYSIS USERMISSING=EXCLUDE
  /CRITERIA ALPHA=0.05 CILEVEL=95.
```

Nonparametric Tests

Notes

Output Created		14-MAR-2018 09:23:40
Comments		
Input	Active Dataset	DataSet0
	Filter	<none>
	Weight	<none>
	Split File	<none>

	N of Rows in Working Data File	116
Syntax		NPTESTS<br>/ONESAMPLE TEST (Neurite_length ZRE_1) WILCOXON(TESTVALUE=1)<br>/MISSING SCOPE=ANALYSIS USERMISSING=EXCLUDE<br>/CRITERIA ALPHA=0.05 CILEVEL=95.
Resources	Processor Time	00:00:01.22
	Elapsed Time	00:00:01.02

null : null

Hypothesis Test Summary

	Null Hypothesis	Test	Sig.	Decision
1	The median of Neurite_length equals 1.00.	One-Sample Wilcoxon Signed Rank Test	.000	Reject the null hypothesis.
2	The median of Standardized Residual for CRMP2_IR equals 1.00.	One-Sample Wilcoxon Signed Rank Test	.000	Reject the null hypothesis.

Asymptotic significances are displayed. The significance level is .05.

Notes

Output Created		14-MAR-2018 09:24:18
Comments		
Input	Active Dataset	DataSet0
	Filter	<none>
	Weight	<none>
	Split File	<none>
	N of Rows in Working Data File	116
Syntax		NPTESTS /ONESAMPLE TEST (ZRE_1) WILCOXON(TESTVALUE=1) /MISSING SCOPE=ANALYSIS USERMISSING=EXCLUDE /CRITERIA ALPHA=0.05 CILEVEL=95.
Resources	Processor Time	00:00:00.09
	Elapsed Time	00:00:00.30

```
*Nonparametric Tests: One Sample.
NPTESTS
  /ONESAMPLE TEST (CRMP2_IR Neurite_length) WILCOXON(TESTVALUE=1)
  /MISSING SCOPE=ANALYSIS USERMISSING=EXCLUDE
  /CRITERIA ALPHA=0.05 CILEVEL=95.
```

Nonparametric Tests

Notes

Output Created		14-MAR-2018 09:25:00
Comments		
Input	Active Dataset	DataSet0
	Filter	<none>
	Weight	<none>
	Split File	<none>
	N of Rows in Working Data File	116
Syntax		NPTESTS /ONESAMPLE TEST (CRMP2_IR Neurite_length) WILCOXON(TESTVALUE=1) /MISSING SCOPE=ANALYSIS USERMISSING=EXCLUDE /CRITERIA ALPHA=0.05 CILEVEL=95.
Resources	Processor Time	00:00:00.08
	Elapsed Time	00:00:00.13

null : null

Hypothesis Test Summary

	Null Hypothesis	Test	Sig.	Decision
1	The median of CRMP2_IR equals 1.0000.	One-Sample Wilcoxon Signed Rank Test	.000	Reject the null hypothesis.
2	The median of Neurite_length equals 1.00.	One-Sample Wilcoxon Signed Rank Test	.000	Reject the null hypothesis.

Asymptotic significances are displayed. The significance level is .05.

Tubulin polymerization assay

```
NEW FILE.
DATASET NAME DataSet2 WINDOW=FRONT.
DATASET ACTIVATE DataSet2.
DATASET CLOSE DataSet1.
EXAMINE VARIABLES=Tub_polymerization
  /PLOT BOXPLOT HISTOGRAM NPPLOT
  /COMPARE GROUPS
  /STATISTICS DESCRIPTIVES
  /CINTERVAL 95
  /MISSING LISTWISE
  /NOTOTAL.
```

Explore

Notes

Output Created		13-MAR-2018 17:59:47
Comments		
Input	Active Dataset	DataSet2
	Filter	<none>
	Weight	<none>
	Split File	<none>
	N of Rows in Working Data File	14
Missing Value Handling	Definition of Missing	User-defined missing values for dependent variables are treated as missing.
	Cases Used	Statistics are based on cases with no missing values for any dependent variable or factor used.

Syntax		EXAMINE VARIABLES=Tub_polymerization<br>/PLOT BOXPLOT HISTOGRAM NPPLOT<br>/COMPARE GROUPS<br>/STATISTICS DESCRIPTIVES<br>/CINTERVAL 95<br>/MISSING LISTWISE<br>/NOTOTAL.
Resources	Processor Time	00:00:00.39
	Elapsed Time	00:00:00.46

Case Processing Summary

	Cases					
	Valid		Missing		Total	
	N	Percent	N	Percent	N	Percent
Tub_polymerization	14	100.0%	0	0.0%	14	100.0%

Descriptives

			Statistic	Std. Error
Tub_polymerization	Mean		39.6659	4.11068
	95% Confidence Interval for Mean	Lower Bound	30.7853	
		Upper Bound	48.5465	
	5% Trimmed Mean		39.0953	
	Median		39.4121	
	Variance		236.567	
	Std. Deviation		15.38074	
	Minimum		18.74	
	Maximum		70.86	
	Range		52.11	
	Interquartile Range		27.81	
	Skewness		.506	.597
	Kurtosis		-.312	1.154

Tests of Normality

	Kolmogorov-Smirnov[a]			Shapiro-Wilk		
	Statistic	df	Sig.	Statistic	df	Sig.
Tub_polymerization	.145	14	.200*	.951	14	.569

*. This is a lower bound of the true significance.

a. Lilliefors Significance Correction

Tub_polymerization

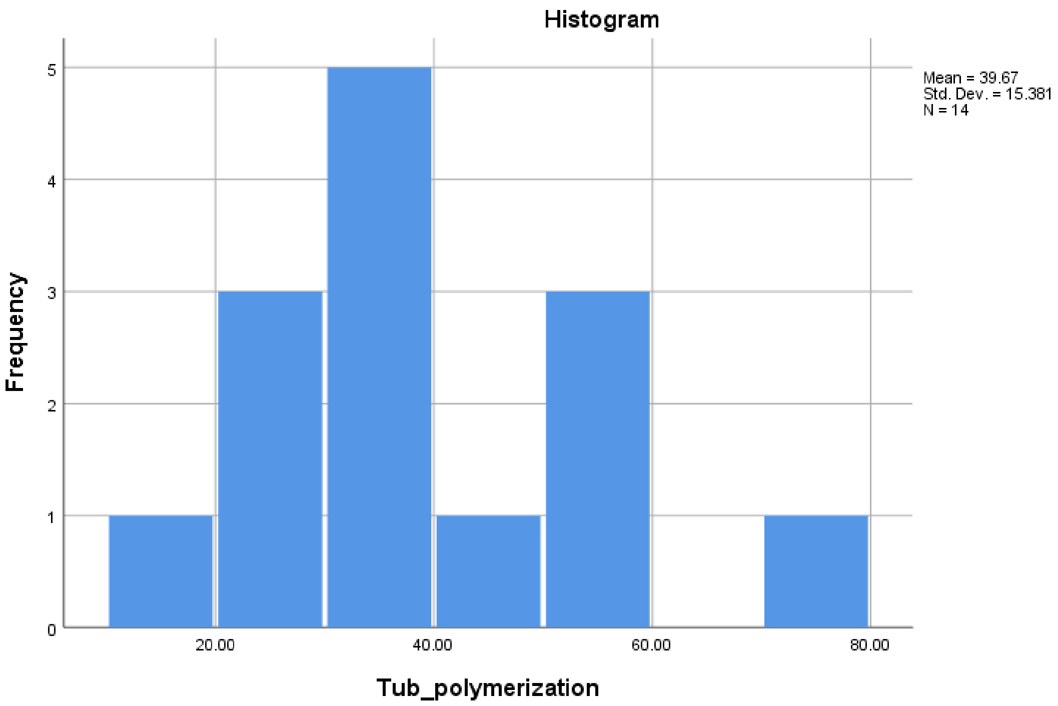

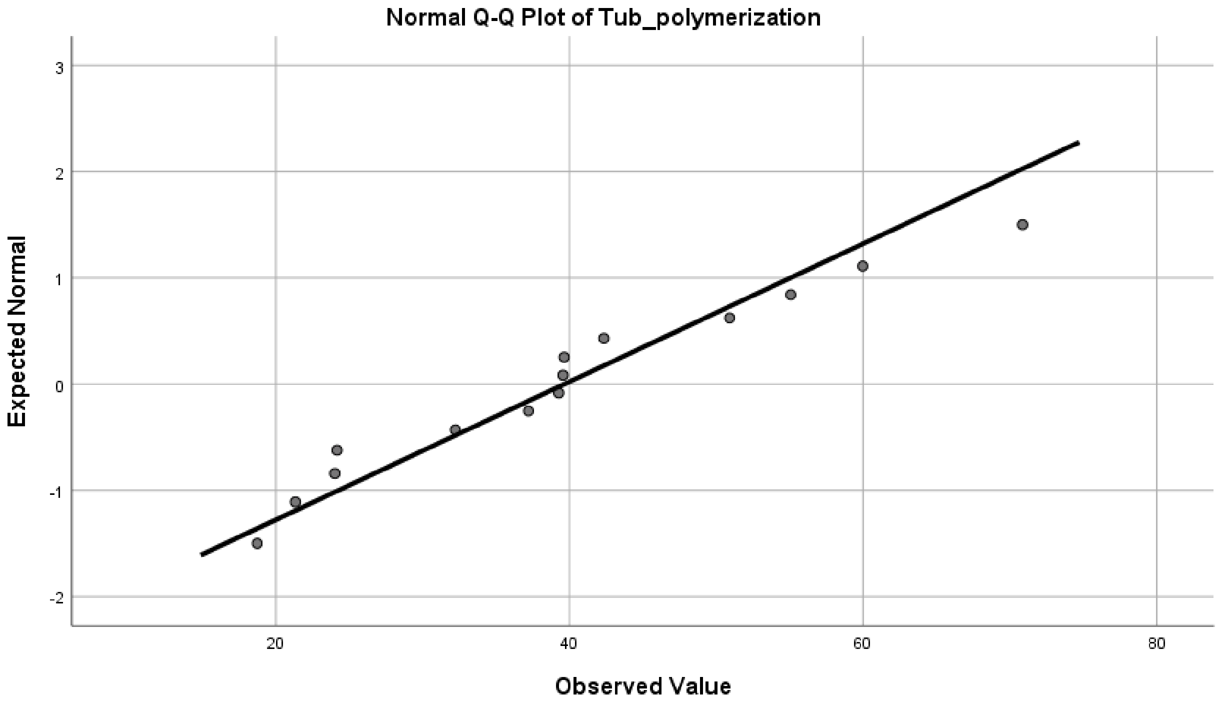

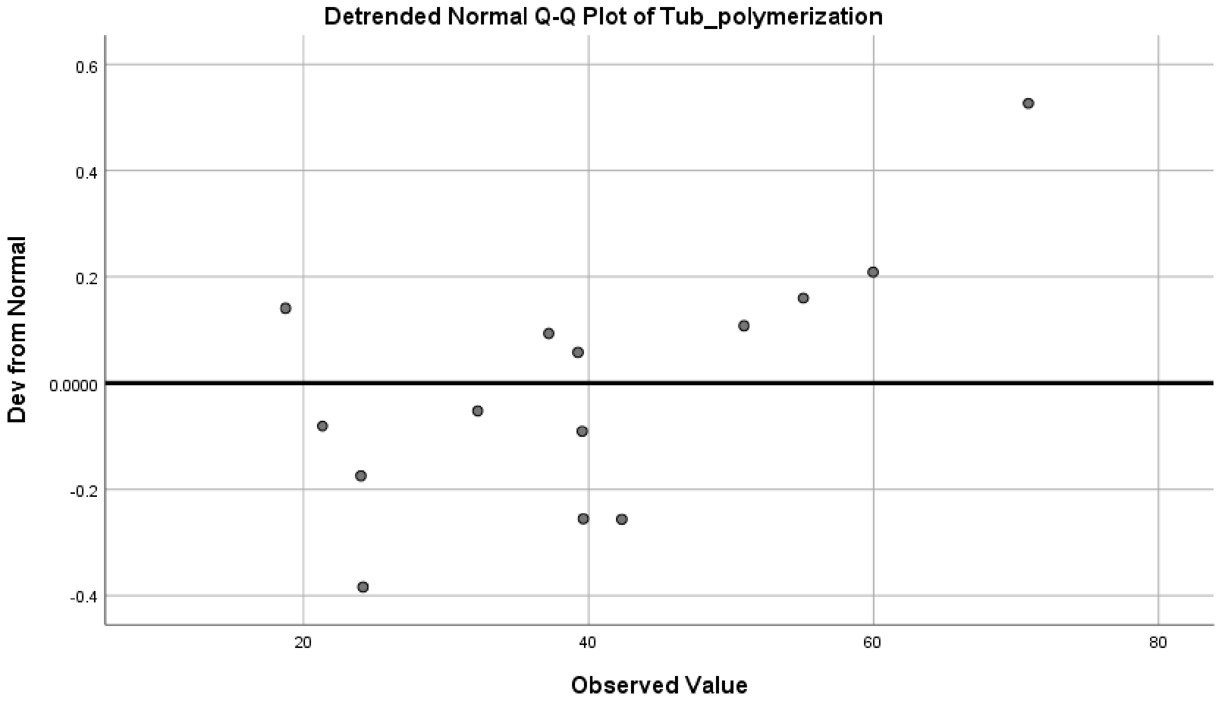


```
T-TEST GROUPS=Treatment(1 2)
  /MISSING=ANALYSIS
```

```
/VARIABLES=Tub_polymerization
/CRITERIA=CI(.95).
```

T-Test

Notes

Output Created		13-MAR-2018 18:00:12
Comments		
Input	Active Dataset	DataSet2
	Filter	<none>
	Weight	<none>
	Split File	<none>
	N of Rows in Working Data File	14
Missing Value Handling	Definition of Missing	User defined missing values are treated as missing.
	Cases Used	Statistics for each analysis are based on the cases with no missing or out-of-range data for any variable in the analysis.
Syntax		T-TEST GROUPS=Treatment(1 2) /MISSING=ANALYSIS /VARIABLES=Tub_polymerization /CRITERIA=CI(.95).
Resources	Processor Time	00:00:00.02
	Elapsed Time	00:00:00.00

Group Statistics

	Treatment	N	Mean	Std. Deviation	Std. Error Mean

Tub_polymerization	Vehicle	7	29.9327	10.01872	3.78672
	Probenecid	7	49.3991	13.82533	5.22548

Independent Samples Test

		Levene's Test for Equality of Variances		t-test for Equality of Means	
		F	Sig.	t	df
Tub_polymerization	Equal variances assumed	.675	.427	-3.017	12
	Equal variances not assumed			-3.017	10.939

Independent Samples Test

		t-test for Equality of Means		
		Sig. (2-tailed)	Mean Difference	Std. Error Difference
Tub_polymerization	Equal variances assumed	.011	-19.46635	6.45329
	Equal variances not assumed	.012	-19.46635	6.45329

Independent Samples Test

		t-test for Equality of Means 95% Confidence Interval of the Difference	
		Lower	Upper
Tub_polymerization	Equal variances assumed	-33.52686	-5.40585
	Equal variances not assumed	-33.67953	-5.25317

```
UNIANOVA Tub_polymerization BY Treatment
  /METHOD=SSTYPE(3)
  /INTERCEPT=INCLUDE
  /SAVE=ZRESID
  /PRINT DESCRIPTIVE HOMOGENEITY
  /CRITERIA=ALPHA(.05)
  /DESIGN=Treatment.
```

Univariate Analysis of Variance

Notes

Output Created		13-MAR-2018 18:01:18
Comments		
Input	Active Dataset	DataSet2
	Filter	<none>
	Weight	<none>
	Split File	<none>
	N of Rows in Working Data File	14
Missing Value Handling	Definition of Missing	User-defined missing values are treated as missing.
	Cases Used	Statistics are based on all cases with valid data for all variables in the model.
Syntax		UNIANOVA Tub_polymerization BY Treatment<br>/METHOD=SSTYPE(3)<br>/INTERCEPT=INCLUDE<br>/SAVE=ZRESID<br>/PRINT DESCRIPTIVE HOMOGENEITY<br>/CRITERIA=ALPHA(.05)<br>/DESIGN=Treatment.
Resources	Processor Time	00:00:00.00
	Elapsed Time	00:00:00.01
Variables Created or Modified	ZRE_1	Standardized Residual for Tub_polymerization

Between-Subjects Factors

		Value Label	N
Treatment	1.00	Vehicle	7
	2.00	Probenecid	7

Descriptive Statistics

Dependent Variable: Tub_polymerization

Treatment	Mean	Std. Deviation	N
Vehicle	29.9327	10.01872	7
Probenecid	49.3991	13.82533	7
Total	39.6659	15.38074	14

Levene's Test of Equality of Error Variances[a,b]

		Levene Statistic	df1	df2	Sig.
Tub_polymerization	Based on Mean	.675	1	12	.427
	Based on Median	.488	1	12	.498
	Based on Median and with adjusted df	.488	1	11.936	.498
	Based on trimmed mean	.710	1	12	.416

Tests the null hypothesis that the error variance of the dependent variable is equal across groups.[a,b]

a. Dependent variable: Tub_polymerization

b. Design: Intercept + Treatment

Tests of Between-Subjects Effects

Dependent Variable: Tub_polymerization

Source	Type III Sum of Squares	df	Mean Square	F	Sig.
Corrected Model	1326.286[a]	1	1326.286	9.099	.011
Intercept	22027.348	1	22027.348	151.124	.000
Treatment	1326.286	1	1326.286	9.099	.011
Error	1749.087	12	145.757		
Total	25102.721	14			
Corrected Total	3075.373	13			

a. R Squared = .431 (Adjusted R Squared = .384)

```
EXAMINE VARIABLES=ZRE_1
  /PLOT HISTOGRAM NPPLOT
  /STATISTICS DESCRIPTIVES
  /CINTERVAL 95
  /MISSING LISTWISE
  /NOTOTAL.
```

Explore

Notes

Output Created		13-MAR-2018 18:01:42
Comments		
Input	Active Dataset	DataSet2
	Filter	<none>
	Weight	<none>
	Split File	<none>
	N of Rows in Working Data File	14
Missing Value Handling	Definition of Missing	User-defined missing values for dependent variables are treated as missing.
	Cases Used	Statistics are based on cases with no missing values for any dependent variable or factor used.
Syntax		EXAMINE VARIABLES=ZRE_1 /PLOT HISTOGRAM NPPLOT /STATISTICS DESCRIPTIVES /CINTERVAL 95 /MISSING LISTWISE /NOTOTAL.
Resources	Processor Time	00:00:00.33
	Elapsed Time	00:00:00.30

Case Processing Summary

Cases

	Valid		Missing		Total	
	N	Percent	N	Percent	N	Percent
Standardized Residual for Tub_polymerization	14	100.0%	0	0.0%	14	100.0%

Descriptives

			Statistic	Std. Error
Standardized Residual for Tub_polymerization	Mean		.0000	.25678
	95% Confidence Interval for Mean	Lower Bound	-.5547	
		Upper Bound	.5547	
	5% Trimmed Mean		-.0197	
	Median		-.1761	
	Variance		.923	
	Std. Deviation		.96077	
	Minimum		-1.42	
	Maximum		1.78	
	Range		3.20	
	Interquartile Range		1.66	
	Skewness		.266	.597
	Kurtosis		-1.086	1.154

Tests of Normality

	Kolmogorov-Smirnov[a]			Shapiro-Wilk		
	Statistic	df	Sig.	Statistic	df	Sig.
Standardized Residual for Tub_polymerization	.190	14	.182	.940	14	.424

a. Lilliefors Significance Correction

Standardized Residual for Tub_polymerization

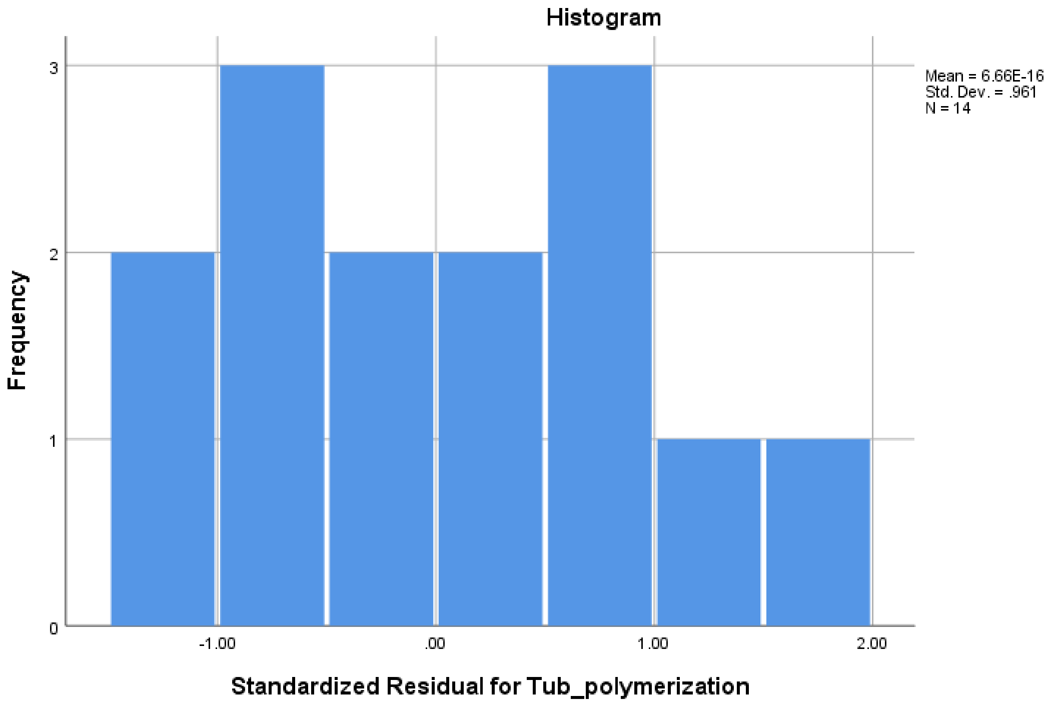
Histogram
Mean = 6.66E-16
Std. Dev. = .961
N = 14
Frequency
3
2
1
0
-1.00
.00
1.00
2.00
Standardized Residual for Tub_polymerization

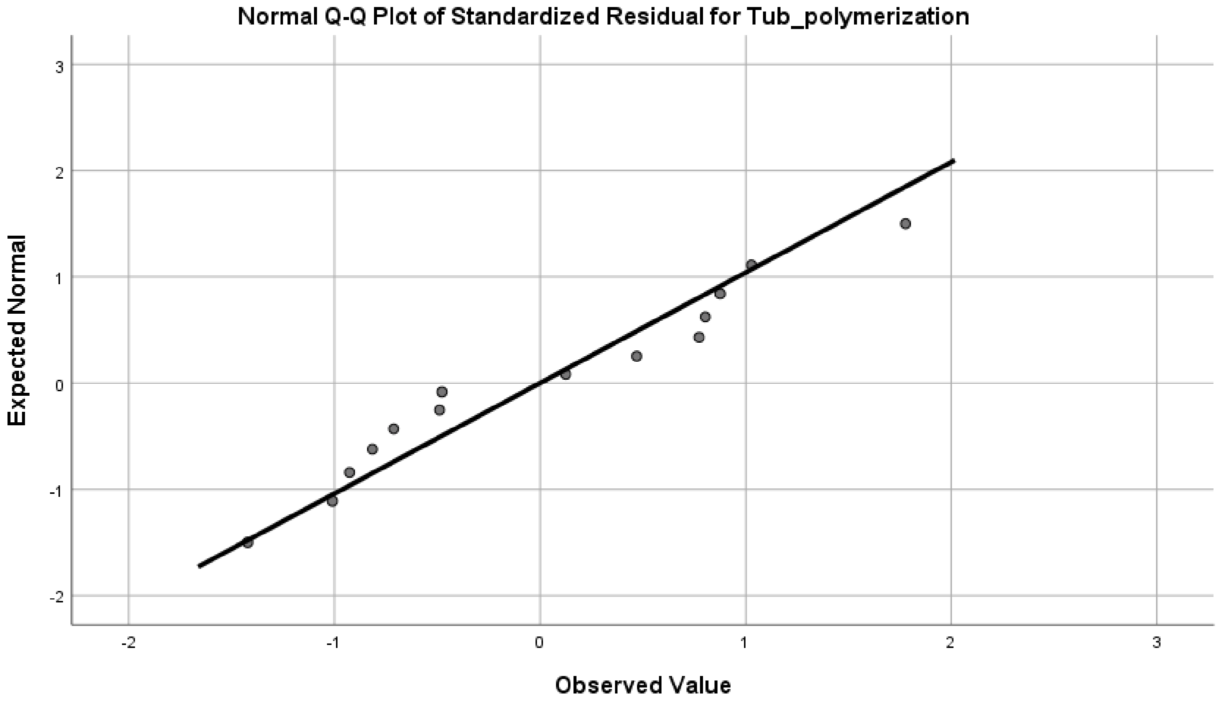
Normal Q-Q Plot of Standardized Residual for Tub_polymerization
Expected Normal
3
2
1
0
-1
-2
-2
-1
0
1
2
3
Observed Value

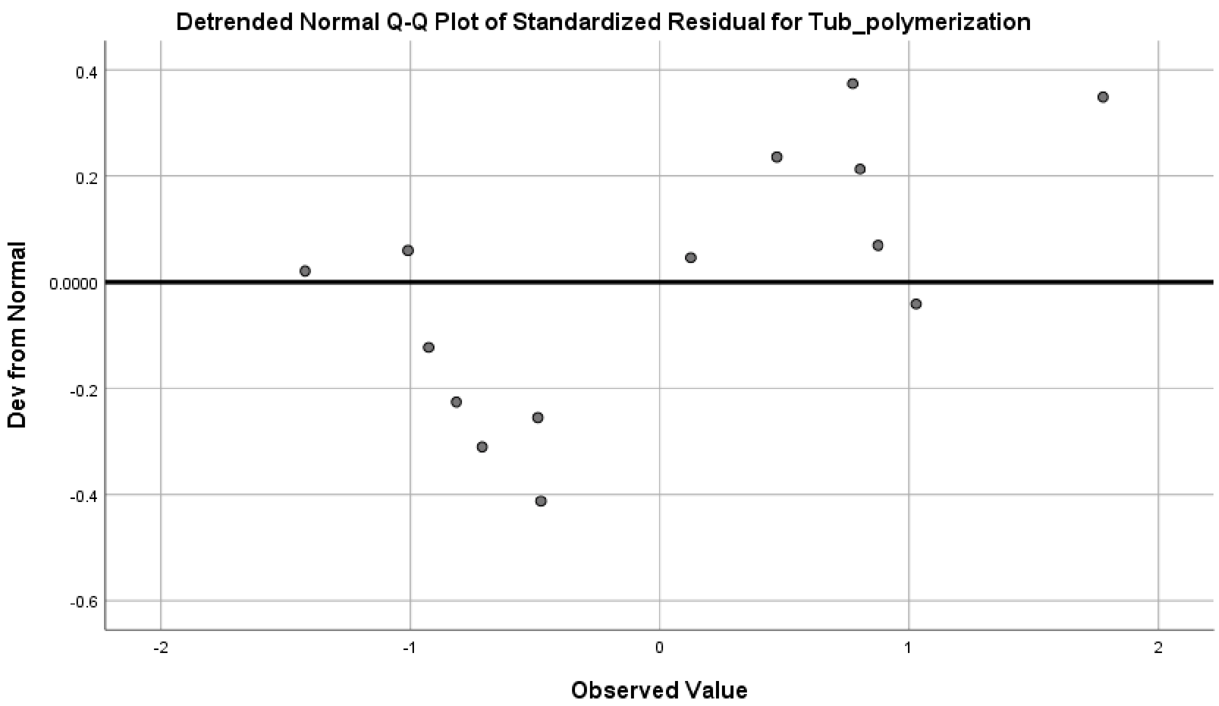
Detrended Normal Q-Q Plot of Standardized Residual for Tub_polymerization
Dev from Normal
Observed Value
0.4
0.2
0.0000
-0.2
-0.4
-0.6
-2
-1
0
1
2

Tubulin post-translational modifications experiments

```
EXAMINE VARIABLES=Ace_tub Tyr_tub
  /PLOT HISTOGRAM NPPLOT
  /STATISTICS DESCRIPTIVES
  /CINTERVAL 95
  /MISSING LISTWISE
  /NOTOTAL.
```

Explore

Notes

Output Created		13-MAR-2018 17:38:23
Comments		
Input	Active Dataset	DataSet1
	Filter	<none>
	Weight	<none>
	Split File	<none>
	N of Rows in Working Data File	18
Missing Value Handling	Definition of Missing	User-defined missing values for dependent variables are treated as missing.
	Cases Used	Statistics are based on cases with no missing values for any dependent variable or factor used.

Syntax		EXAMINE VARIABLES=Ace_tub Tyr_tub<br>/PLOT HISTOGRAM NPPLOT<br>/STATISTICS DESCRIPTIVES<br>/CINTERVAL 95<br>/MISSING LISTWISE<br>/NOTOTAL.
Resources	Processor Time	00:00:00.66
	Elapsed Time	00:00:00.55

Case Processing Summary

	Cases					
	Valid		Missing		Total	
	N	Percent	N	Percent	N	Percent
Ace_tub	18	100.0%	0	0.0%	18	100.0%
Tyr_tub	18	100.0%	0	0.0%	18	100.0%

Descriptives

			Statistic	Std. Error
Ace_tub	Mean		116.1467	6.12219
	95% Confidence Interval for Mean	Lower Bound	103.2300	
		Upper Bound	129.0634	
	5% Trimmed Mean		115.6655	
	Median		114.4930	
	Variance		674.662	
	Std. Deviation		25.97425	
	Minimum		71.30	
	Maximum		169.65	
	Range		98.35	
	Interquartile Range		37.88	
	Skewness		.199	.536
	Kurtosis		-.249	1.038
Tyr_tub	Mean		86.6378	6.57484

			Statistic	Std. Error
	95% Confidence Interval for Mean	Lower Bound	72.7661	
		Upper Bound	100.5095	
	5% Trimmed Mean		84.4857	
	Median		81.8655	
	Variance		778.112	
	Std. Deviation		27.89467	
	Minimum		44.58	
	Maximum		167.43	
	Range		122.86	
	Interquartile Range		34.95	
	Skewness		1.288	.536
	Kurtosis		3.172	1.038

Tests of Normality

	Kolmogorov-Smirnov[a]			Shapiro-Wilk		
	Statistic	df	Sig.	Statistic	df	Sig.
Ace_tub	.091	18	.200*	.984	18	.984
Tyr_tub	.138	18	.200*	.914	18	.100

*. This is a lower bound of the true significance.

a. Lilliefors Significance Correction

Ace_tub

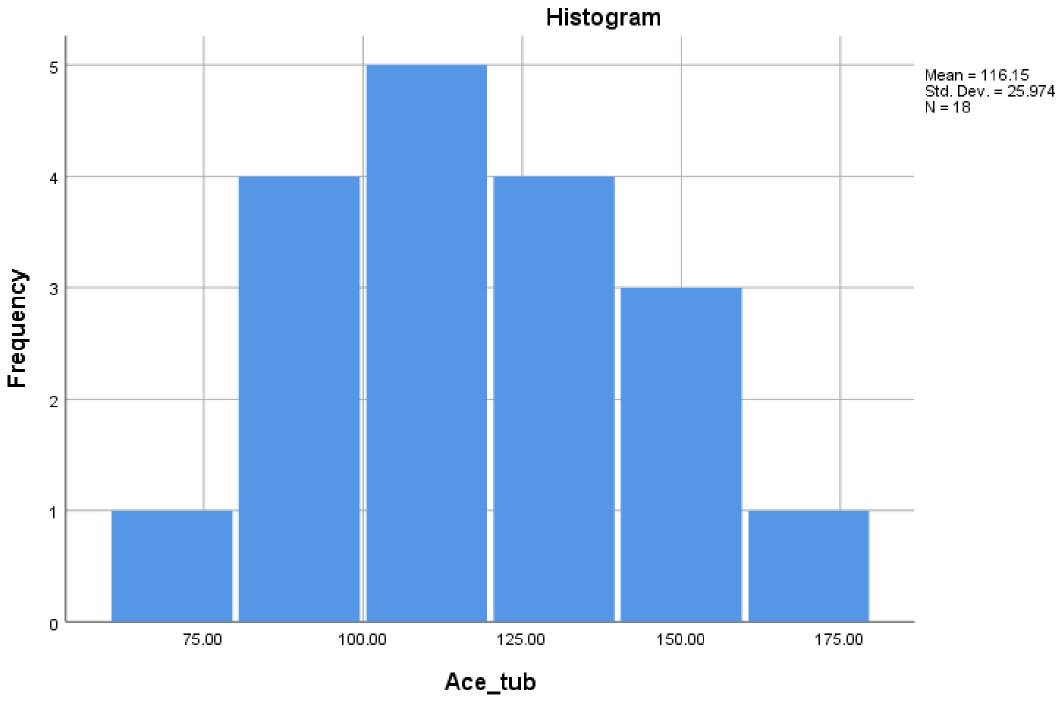
Histogram
Mean = 116.15
Std. Dev. = 25.974
N = 18
Frequency
5
4
3
2
1
0
75.00
100.00
125.00
150.00
175.00
Ace_tub

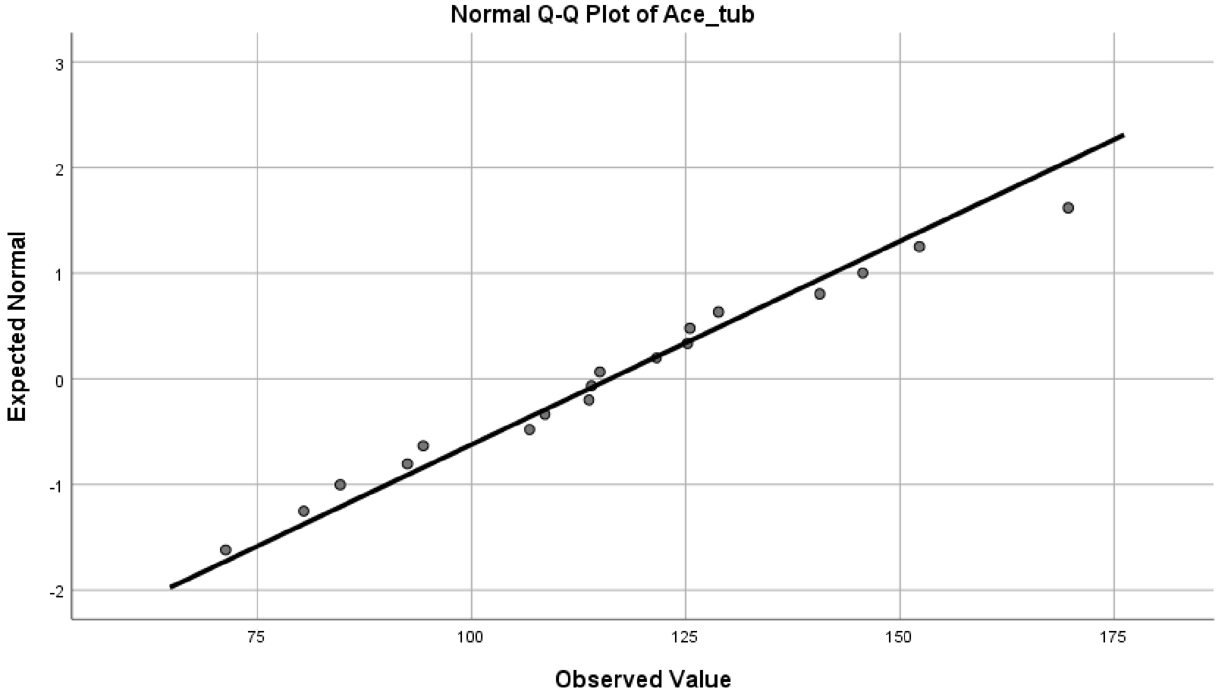
Normal Q-Q Plot of Ace_tub
Expected Normal
3
2
1
0
-1
-2
75
100
125
150
175
Observed Value

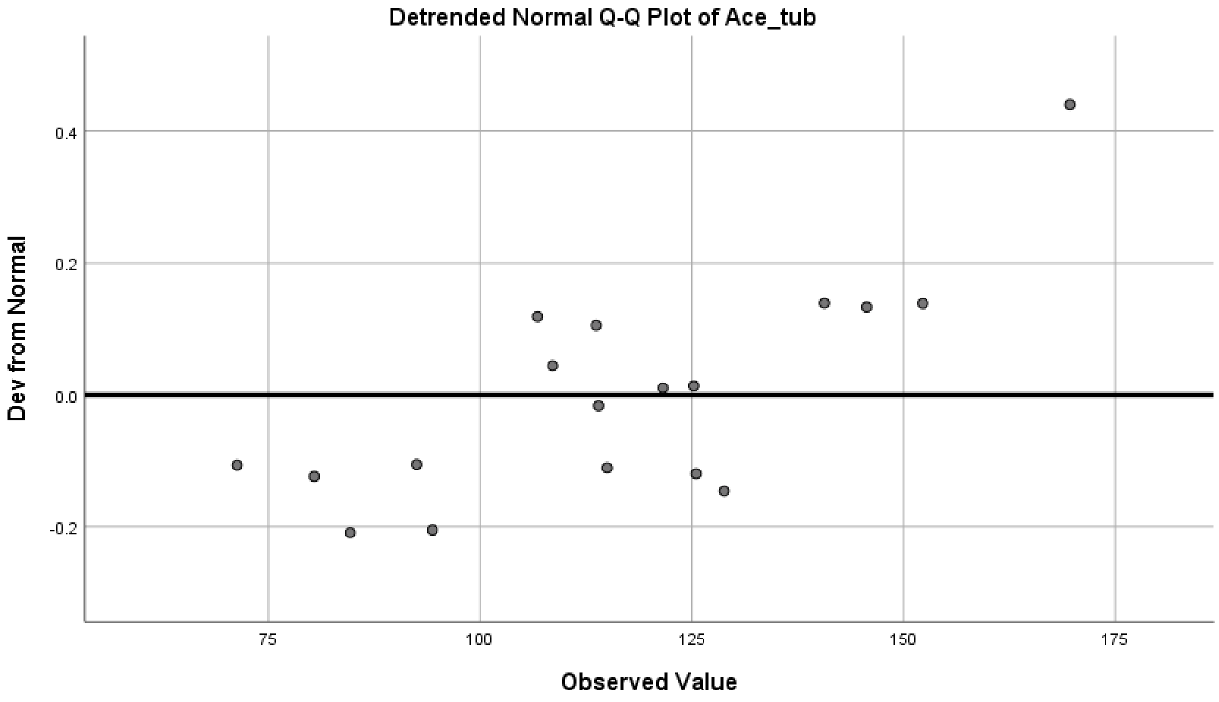
Detrended Normal Q-Q Plot of Ace_tub
Dev from Normal
0.4
0.2
0.0
-0.2
75
100
125
150
175
Observed Value

Tyr_tub

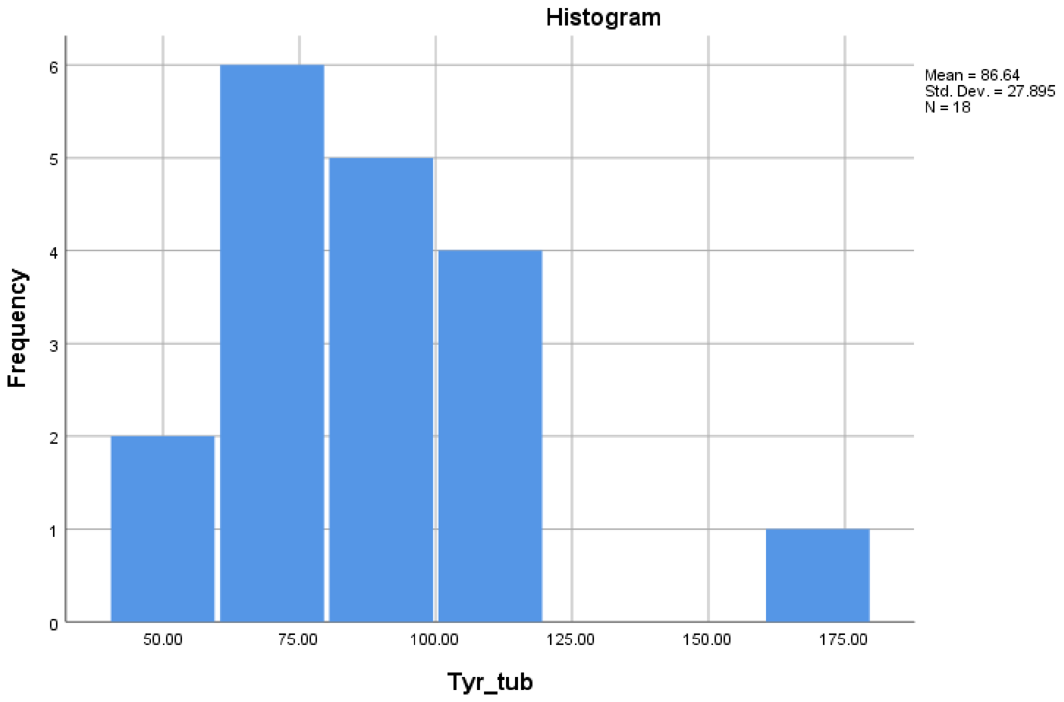
Histogram
Mean = 86.64
Std. Dev. = 27.895
N = 18
Frequency
0
1
2
3
4
5
6
50.00
75.00
100.00
125.00
150.00
175.00
Tyr_tub

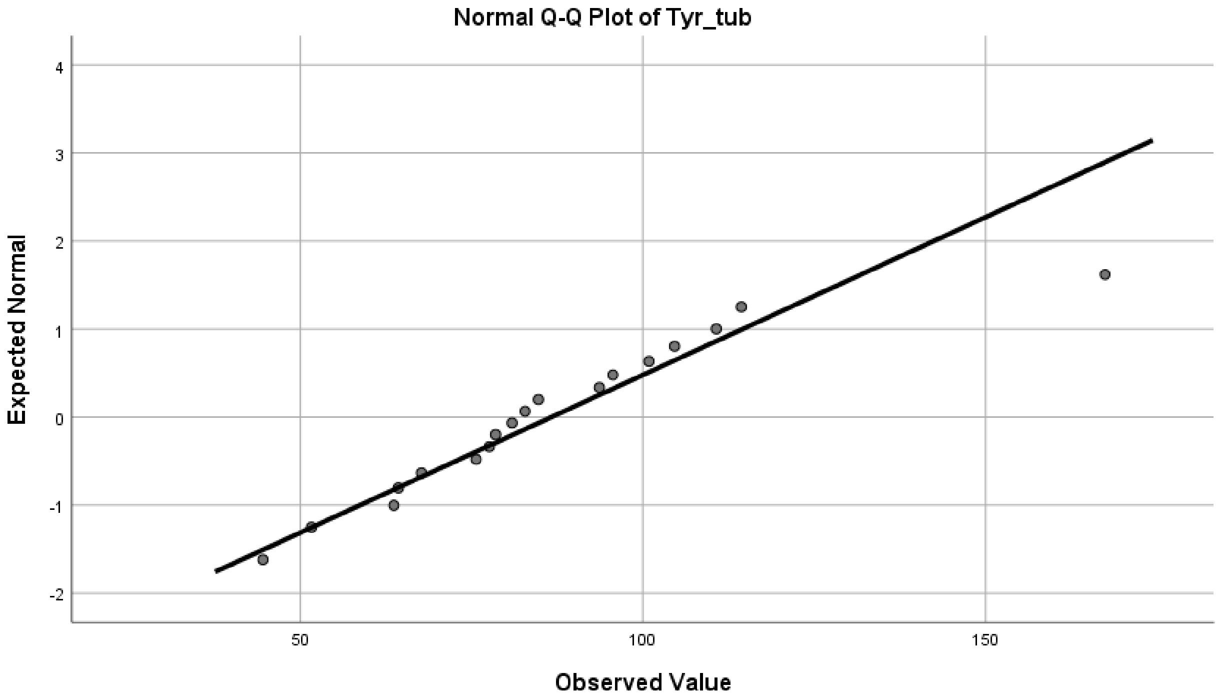
Normal Q-Q Plot of Tyr_tub
Expected Normal
4
3
2
1
0
-1
-2
50
100
150
Observed Value

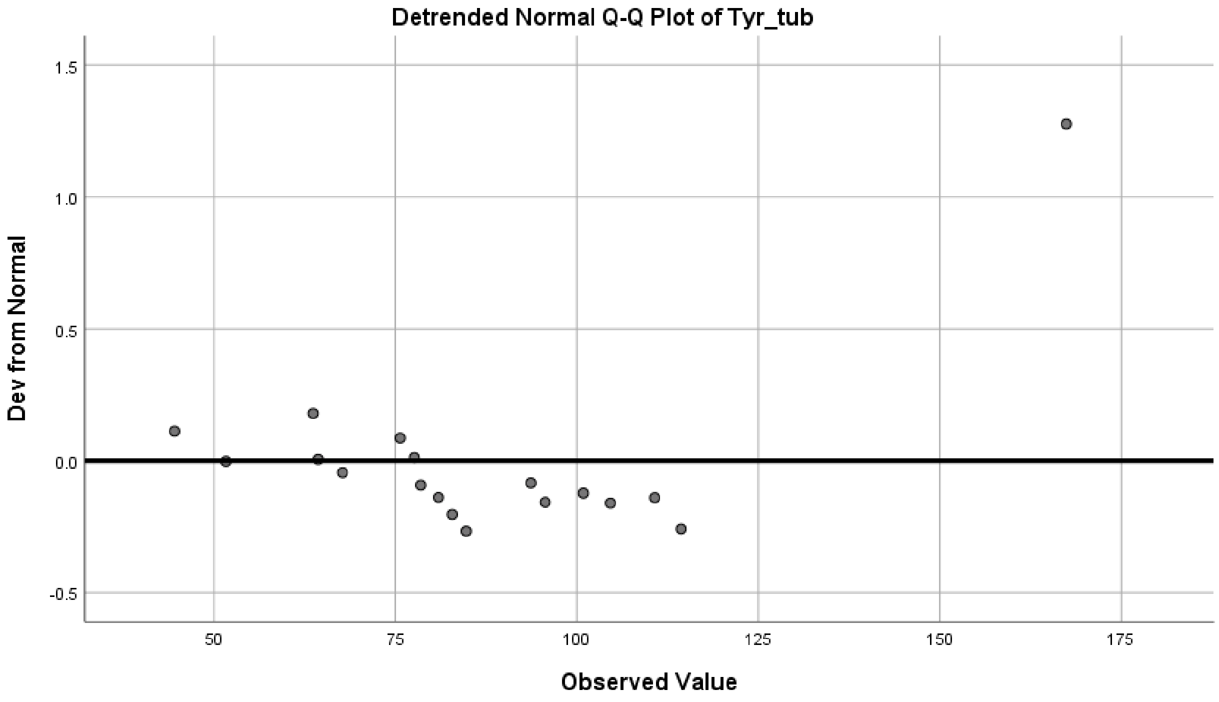


```
T-TEST GROUPS=Treatment(1 2)
  /MISSING=ANALYSIS
  /VARIABLES=Ace_tub
  /CRITERIA=CI(.95).
```

T-Test

Notes

Output Created		13-MAR-2018 17:39:06
Comments		
Input	Active Dataset	DataSet1
	Filter	<none>
	Weight	<none>
	Split File	<none>
	N of Rows in Working Data File	18

Missing Value Handling	Definition of Missing	User defined missing values are treated as missing.
	Cases Used	Statistics for each analysis are based on the cases with no missing or out-of-range data for any variable in the analysis.
Syntax		T-TEST GROUPS=Treatment(1 2)<br>/MISSING=ANALYSIS<br>/VARIABLES=Ace_tub<br>/CRITERIA=CI(.95).
Resources	Processor Time	00:00:00.00
	Elapsed Time	00:00:00.00

Group Statistics

	Treatment	N	Mean	Std. Deviation	Std. Error Mean
Ace_tub	Vehicle	9	100.0000	18.17438	6.05813
	Probenecid	9	132.2935	22.73180	7.57727

Independent Samples Test

		Levene's Test for Equality of Variances		t-test for Equality of Means	
		F	Sig.	t	df
Ace_tub	Equal variances assumed	.183	.675	-3.329	16
	Equal variances not assumed			-3.329	15.261

Independent Samples Test

		t-test for Equality of Means			
		Sig. (2-tailed)	Mean Difference	Std. Error Difference	95% Confidence Interval of the Difference Lower
Ace_tub	Equal variances assumed	.004	-32.29347	9.70134	-52.85938
	Equal variances not assumed	.004	-32.29347	9.70134	-52.94064

Independent Samples Test

		t-test for Equality of Means
		95% Confidence Interval of the Difference
		Upper
Ace_tub	Equal variances assumed	-11.72755
	Equal variances not assumed	-11.64629

```
T-TEST GROUPS=Treatment(1 2)
  /MISSING=ANALYSIS
  /VARIABLES=Tyr_tub
  /CRITERIA=CI(.95).
```

T-Test

Notes

Output Created		13-MAR-2018 17:39:18
Comments		
Input	Active Dataset	DataSet1
	Filter	<none>
	Weight	<none>
	Split File	<none>
	N of Rows in Working Data File	18
Missing Value Handling	Definition of Missing	User defined missing values are treated as missing.
	Cases Used	Statistics for each analysis are based on the cases with no missing or out-of-range data for any variable in the analysis.

Syntax		T-TEST<br>GROUPS=Treatment(1 2)<br>/MISSING=ANALYSIS<br>/VARIABLES=Tyr_tub<br>/CRITERIA=CI(.95).
Resources	Processor Time	00:00:00.02
	Elapsed Time	00:00:00.00

Group Statistics

	Treatment	N	Mean	Std. Deviation	Std. Error Mean
Tyr_tub	Vehicle	9	100.0000	31.35090	10.45030
	Probenecid	9	73.2756	16.39744	5.46581

Independent Samples Test

		Levene's Test for Equality of Variances		t-test for Equality of Means	
		F	Sig.	t	df
Tyr_tub	Equal variances assumed	1.195	.290	2.266	16
	Equal variances not assumed			2.266	12.072

Independent Samples Test

		t-test for Equality of Means			
					95% Confidence Interval of the Difference
		Sig. (2-tailed)	Mean Difference	Std. Error Difference	Lower
Tyr_tub	Equal variances assumed	.038	26.72440	11.79338	1.72354
	Equal variances not assumed	.043	26.72440	11.79338	1.04586

Independent Samples Test

		t-test for Equality of Means
		95% Confidence Interval of the Difference
		Upper
Tyr_tub	Equal variances assumed	51.72525

Equal variances not assumed	52.40294

```
UNIANOVA Ace_tub BY Treatment
  /METHOD=SSTYPE(3)
  /INTERCEPT=INCLUDE
  /SAVE=ZRESID
  /PRINT DESCRIPTIVE HOMOGENEITY
  /CRITERIA=ALPHA(.05)
  /DESIGN=Treatment.
```

Univariate Analysis of Variance

Notes

Output Created		13-MAR-2018 17:40:09
Comments		
Input	Active Dataset	DataSet1
	Filter	<none>
	Weight	<none>
	Split File	<none>
	N of Rows in Working Data File	18
Missing Value Handling	Definition of Missing	User-defined missing values are treated as missing.
	Cases Used	Statistics are based on all cases with valid data for all variables in the model.
Syntax		UNIANOVA Ace_tub BY Treatment<br>/METHOD=SSTYPE(3)<br>/INTERCEPT=INCLUDE<br>/SAVE=ZRESID<br>/PRINT DESCRIPTIVE HOMOGENEITY<br>/CRITERIA=ALPHA(.05)<br>/DESIGN=Treatment.

Resources	Processor Time	00:00:00.02
	Elapsed Time	00:00:00.01
Variables Created or Modified	ZRE_1	Standardized Residual for Ace_tub

Between-Subjects Factors

		Value Label	N
Treatment	1	Vehicle	9
	2	Probenecid	9

Descriptive Statistics

Dependent Variable: Ace_tub

Treatment	Mean	Std. Deviation	N
Vehicle	100.0000	18.17438	9
Probenecid	132.2935	22.73180	9
Total	116.1467	25.97425	18

Levene's Test of Equality of Error Variances[a,b]

		Levene Statistic	df1	df2	Sig.
Ace_tub	Based on Mean	.183	1	16	.675
	Based on Median	.172	1	16	.684
	Based on Median and with adjusted df	.172	1	15.555	.684
	Based on trimmed mean	.189	1	16	.670

Tests the null hypothesis that the error variance of the dependent variable is equal across groups.[a,b]

a. Dependent variable: Ace_tub

b. Design: Intercept + Treatment

Tests of Between-Subjects Effects

Dependent Variable: Ace_tub

Source	Type III Sum of Squares	df	Mean Square	F	Sig.
Corrected Model	4692.906[a]	1	4692.906	11.081	.004
Intercept	242821.139	1	242821.139	573.338	.000
Treatment	4692.906	1	4692.906	11.081	.004
Error	6776.345	16	423.522		
Total	254290.390	18			
Corrected Total	11469.251	17			

a. R Squared = .409 (Adjusted R Squared = .372)

```
EXAMINE VARIABLES=ZRE_1
  /PLOT HISTOGRAM NPPLOT
  /STATISTICS DESCRIPTIVES
  /CINTERVAL 95
  /MISSING LISTWISE
  /NOTOTAL.
```

Explore

Notes

Output Created		13-MAR-2018 17:44:44
Comments		
Input	Active Dataset	DataSet1
	Filter	<none>
	Weight	<none>
	Split File	<none>
	N of Rows in Working Data File	18
Missing Value Handling	Definition of Missing	User-defined missing values for dependent variables are treated as missing.

	Cases Used	Statistics are based on cases with no missing values for any dependent variable or factor used.
Syntax		EXAMINE VARIABLES=ZRE_1<br>/PLOT HISTOGRAM NPPLOT<br>/STATISTICS DESCRIPTIVES<br>/CINTERVAL 95<br>/MISSING LISTWISE<br>/NOTOTAL.
Resources	Processor Time	00:00:00.34
	Elapsed Time	00:00:00.28

Case Processing Summary

	Cases					
	Valid		Missing		Total	
	N	Percent	N	Percent	N	Percent
Standardized Residual for Ace_tub	18	100.0%	0	0.0%	18	100.0%

Descriptives

			Statistic	Std. Error
Standardized Residual for Ace_tub	Mean		.0000	.22866
	95% Confidence Interval for Mean	Lower Bound	-.4824	
		Upper Bound	.4824	
	5% Trimmed Mean		.0066	
	Median		.0801	
	Variance		.941	
	Std. Deviation		.97014	
	Minimum		-1.93	
	Maximum		1.82	
	Range		3.75	
	Interquartile Range		1.46	

	Skewness	-.163	.536
	Kurtosis	-.364	1.038

Tests of Normality

	Kolmogorov-Smirnov[a]			Shapiro-Wilk		
	Statistic	df	Sig.	Statistic	df	Sig.
Standardized Residual for Ace_tub	.133	18	.200*	.986	18	.989

*. This is a lower bound of the true significance.

a. Lilliefors Significance Correction

Standardized Residual for Ace_tub

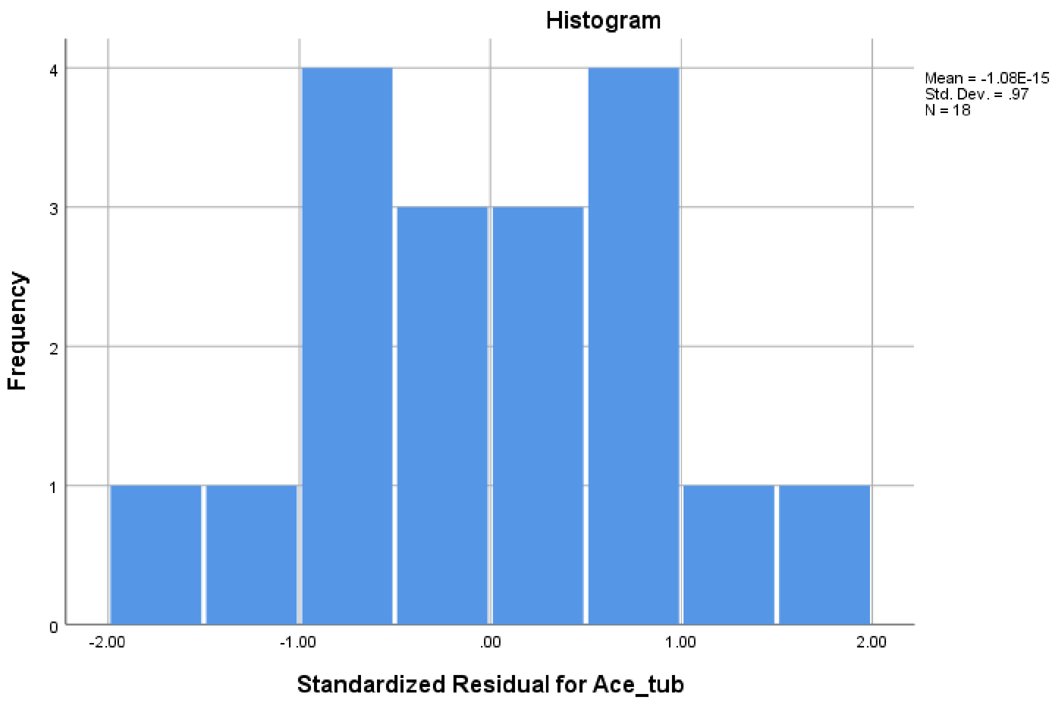

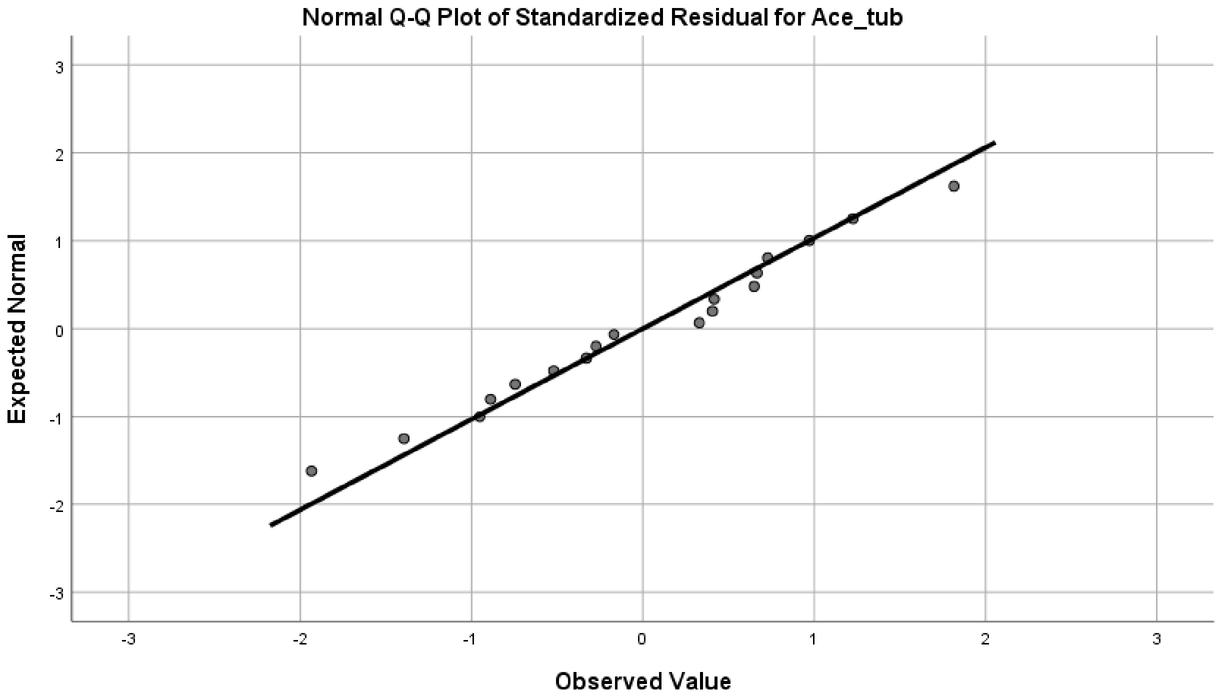

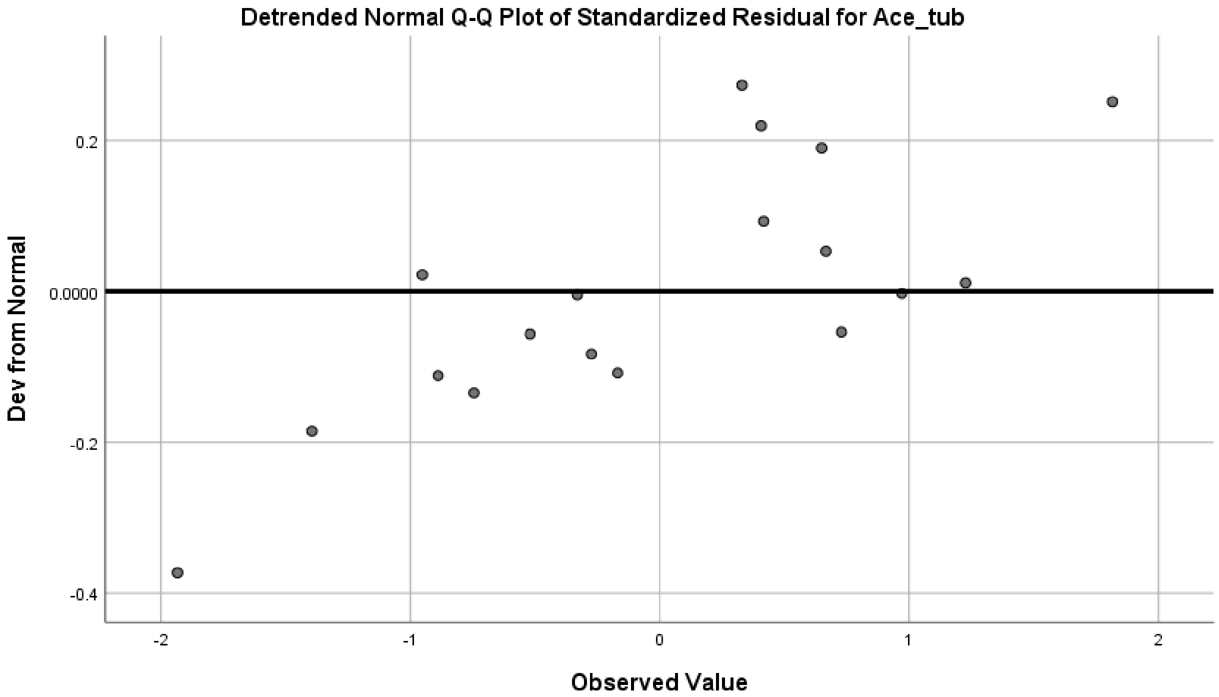


```
UNIANOVA Tyr_tub BY Treatment
  /METHOD=SSTYPE(3)
```

```
/INTERCEPT=INCLUDE
/SAVE=ZRESID
/PRINT DESCRIPTIVE HOMOGENEITY
/CRITERIA=ALPHA(.05)
/DESIGN=Treatment.
```

Univariate Analysis of Variance

Notes

Output Created		13-MAR-2018 17:45:15
Comments		
Input	Active Dataset	DataSet1
	Filter	<none>
	Weight	<none>
	Split File	<none>
	N of Rows in Working Data File	18
Missing Value Handling	Definition of Missing	User-defined missing values are treated as missing.
	Cases Used	Statistics are based on all cases with valid data for all variables in the model.
Syntax		UNIANOVA Tyr_tub BY Treatment /METHOD=SSTYPE(3) /INTERCEPT=INCLUDE /SAVE=ZRESID /PRINT DESCRIPTIVE HOMOGENEITY /CRITERIA=ALPHA(.05) /DESIGN=Treatment.
Resources	Processor Time	00:00:00.02
	Elapsed Time	00:00:00.01
Variables Created or Modified	ZRE_2	Standardized Residual for Tyr_tub

Between-Subjects Factors

		Value Label	N
Treatment	1	Vehicle	9
	2	Probenecid	9

Descriptive Statistics

Dependent Variable: Tyr_tub

Treatment	Mean	Std. Deviation	N
Vehicle	100.0000	31.35090	9
Probenecid	73.2756	16.39744	9
Total	86.6378	27.89467	18

Levene's Test of Equality of Error Variances[a,b]

		Levene Statistic	df1	df2	Sig.
Tyr_tub	Based on Mean	1.195	1	16	.290
	Based on Median	1.040	1	16	.323
	Based on Median and with adjusted df	1.040	1	11.560	.329
	Based on trimmed mean	1.148	1	16	.300

Tests the null hypothesis that the error variance of the dependent variable is equal across groups.[a,b]

a. Dependent variable: Tyr_tub

b. Design: Intercept + Treatment

Tests of Between-Subjects Effects

Dependent Variable: Tyr_tub

Source	Type III Sum of Squares	df	Mean Square	F	Sig.
Corrected Model	3213.871[a]	1	3213.871	5.135	.038
Intercept	135109.960	1	135109.960	215.873	.000
Treatment	3213.871	1	3213.871	5.135	.038

Error	10014.040	16	625.877		
Total	148337.870	18			
Corrected Total	13227.911	17			

a. R Squared = .243 (Adjusted R Squared = .196)

```
EXAMINE VARIABLES=ZRE_2
  /PLOT HISTOGRAM NPPLOT
  /STATISTICS DESCRIPTIVES
  /CINTERVAL 95
  /MISSING LISTWISE
  /NOTOTAL.
```

Explore

Notes

Output Created		13-MAR-2018 17:46:33
Comments		
Input	Active Dataset	DataSet1
	Filter	<none>
	Weight	<none>
	Split File	<none>
	N of Rows in Working Data File	18
Missing Value Handling	Definition of Missing	User-defined missing values for dependent variables are treated as missing.
	Cases Used	Statistics are based on cases with no missing values for any dependent variable or factor used.

Syntax		EXAMINE VARIABLES=ZRE_2<br>/PLOT HISTOGRAM NPPLOT<br>/STATISTICS DESCRIPTIVES<br>/CINTERVAL 95<br>/MISSING LISTWISE<br>/NOTOTAL.
Resources	Processor Time	00:00:00.39
	Elapsed Time	00:00:00.27

Case Processing Summary

	Cases					
	Valid		Missing		Total	
	N	Percent	N	Percent	N	Percent
Standardized Residual for Tyr_tub	18	100.0%	0	0.0%	18	100.0%

Descriptives

			Statistic	Std. Error
Standardized Residual for Tyr_tub	Mean		.0000	.22866
	95% Confidence Interval for Mean	Lower Bound	-.4824	
		Upper Bound	.4824	
	5% Trimmed Mean		-.0424	
	Median		-.0697	
	Variance		.941	
	Std. Deviation		.97014	
	Minimum		-1.93	
	Maximum		2.70	
	Range		4.63	
	Interquartile Range		.83	
	Skewness		.895	.536
	Kurtosis		3.166	1.038

Tests of Normality

	Kolmogorov-Smirnov[a]			Shapiro-Wilk		
	Statistic	df	Sig.	Statistic	df	Sig.
Standardized Residual for Tyr_tub	.166	18	.200*	.920	18	.132

*. This is a lower bound of the true significance.

a. Lilliefors Significance Correction

Standardized Residual for Tyr_tub

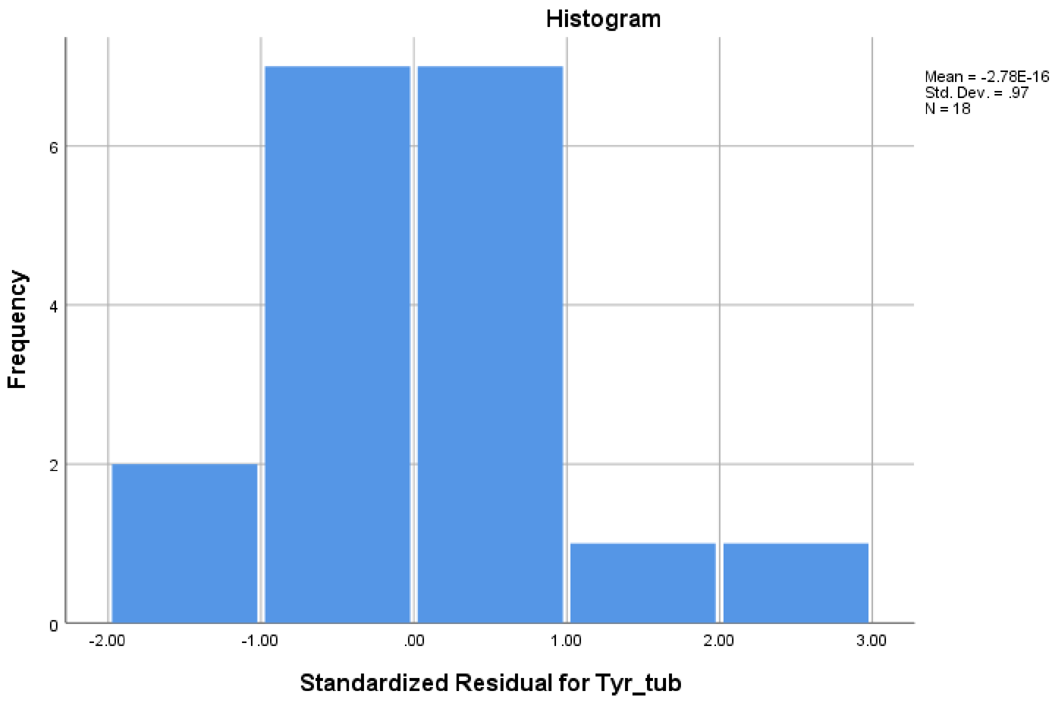

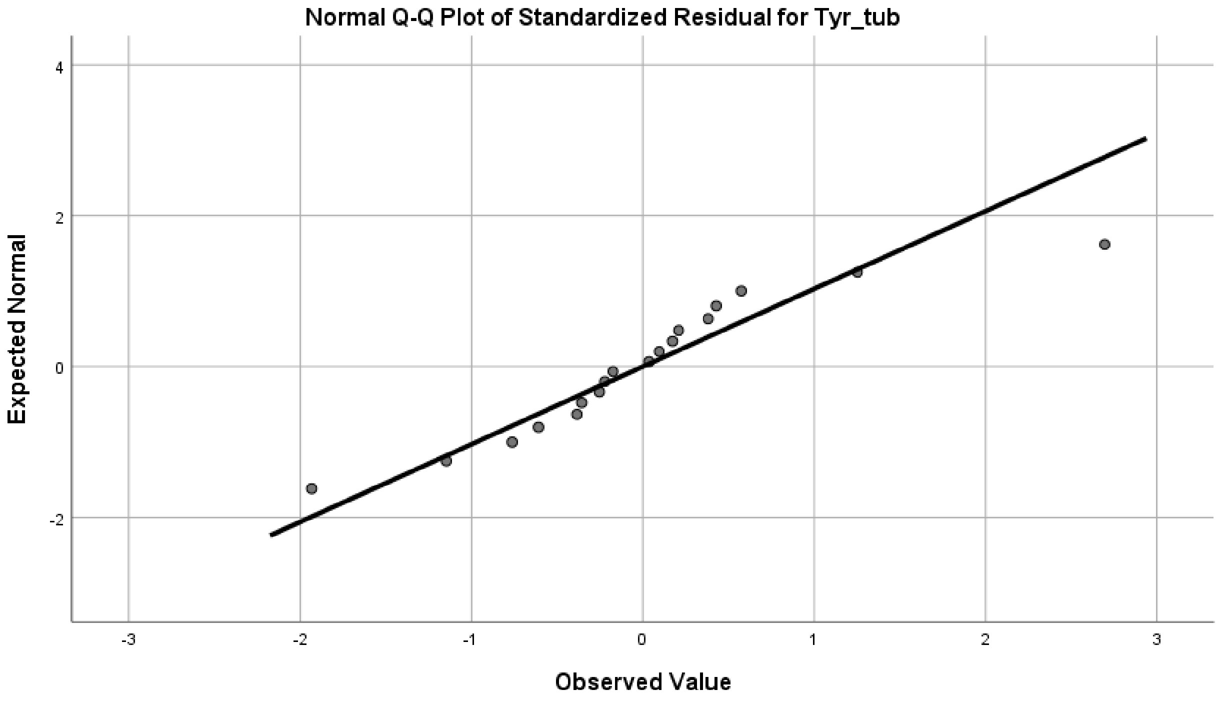
Normal Q-Q Plot of Standardized Residual for Tyr_tub
Expected Normal
Observed Value
4
2
0
-2
-3
-2
-1
0
1
2
3

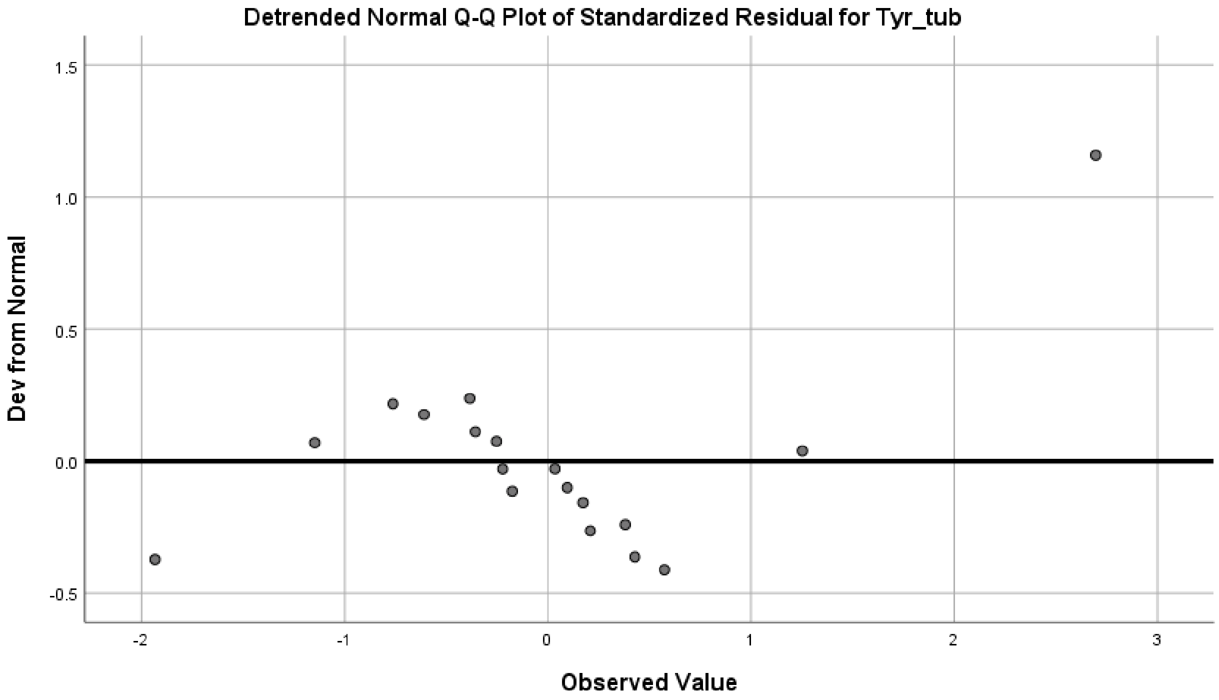
Detrended Normal Q-Q Plot of Standardized Residual for Tyr_tub
Dev from Normal
Observed Value
1.5
1.0
0.5
0.0
-0.5
-2
-1
0
1
2
3

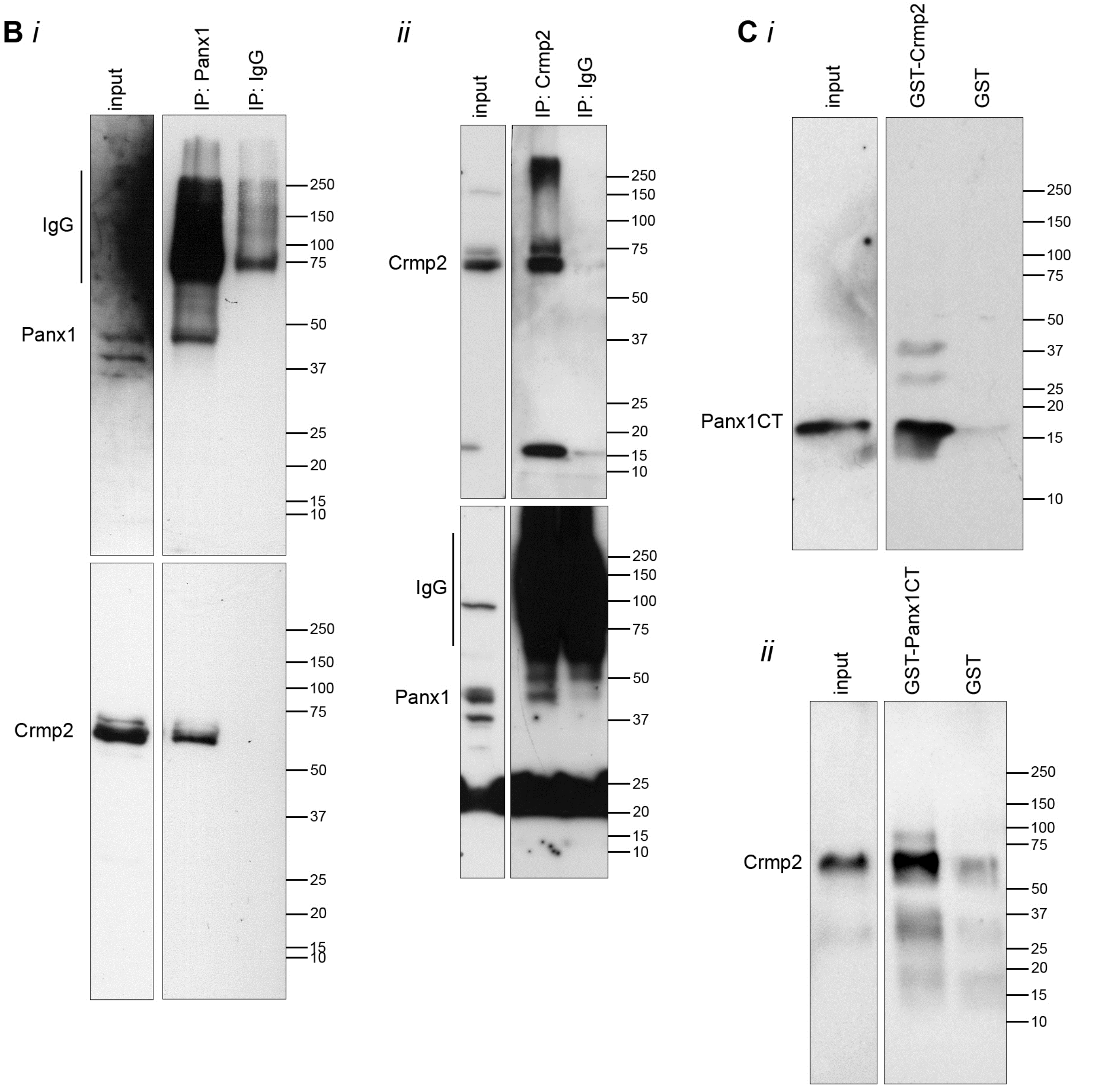
B i
input
IP: Panx1
IP: IgG
IgG
Panx1
Crmp2
250
150
100
75
50
37
25
20
15
10
ii
input
IP: Crmp2
IP: IgG
Crmp2
IgG
Panx1
C i
input
GST-Crmp2
GST
Panx1CT
ii
input
GST-Panx1CT
GST
Crmp2

Supplement for Figure 2

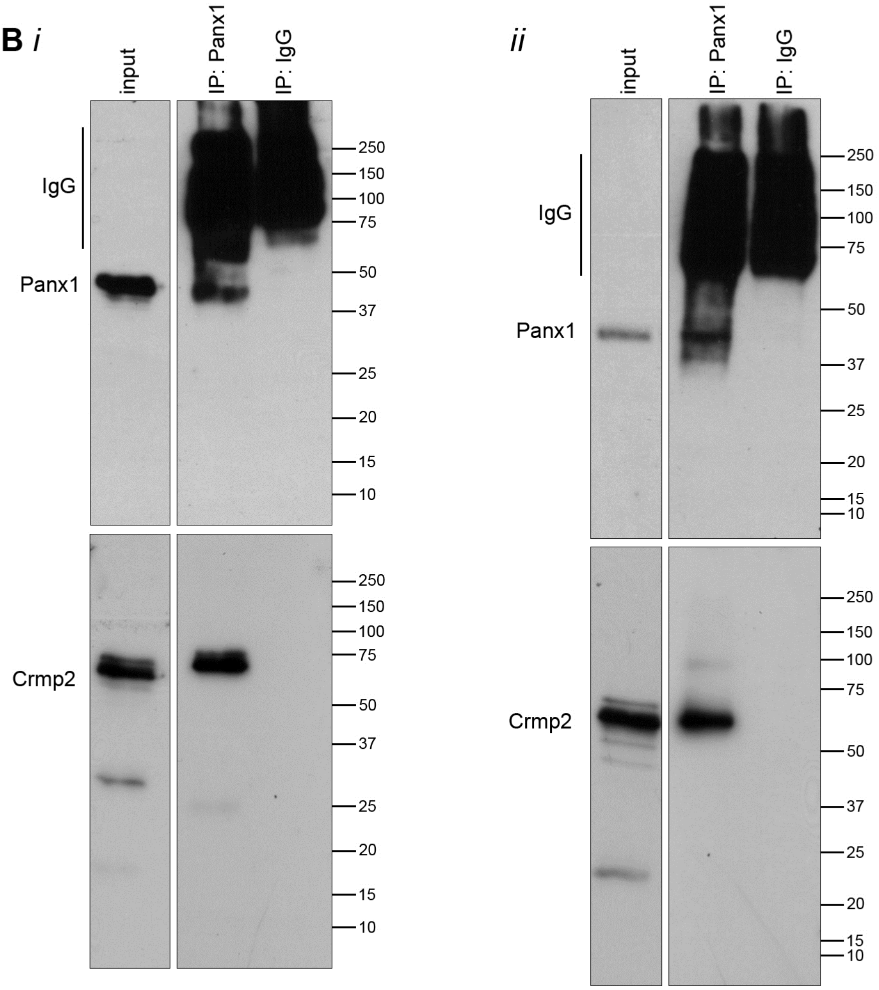

Supplement: Supplementary file 1 [file Presentation_1.PDF]
